# Natural Products from Actinobacteria as a Potential Source of New Therapies Against Colorectal Cancer: A Review

**DOI:** 10.3389/fphar.2022.929161

**Published:** 2022-07-11

**Authors:** Yadollah Bahrami, Sasan Bouk, Elham Kakaei, Mohammad Taheri

**Affiliations:** ^1^ Department of Medical Biotechnology, School of Medicine, Kermanshah University of Medical Sciences, Kermanshah, Iran; ^2^ Pharmaceutical Research Center, Kermanshah University of Medical Sciences, Kermanshah, Iran; ^3^ Department of Medical Biotechnology, School of Medicine, College of Medicine and Public Health, Flinders University, Adelaide, SA, Australia; ^4^ Institute of Human Genetics, University Hospital Jena, Jena, Germany; ^5^ Urology and Nephrology Research Center, Shahid Beheshti University of Medical Sciences, Tehran, Iran

**Keywords:** colorectal cancer, natural products, actinobacteria, secondary metabolites, anti-cancer effects, marine actinobacteria, endophytic actinobacteria, bioactive compounds

## Abstract

Colorectal cancer (CRC) is a common, and deadly disease. Despite the improved knowledge on CRC heterogeneity and advances in the medical sciences, there is still an urgent need to cope with the challenges and side effects of common treatments for the disease. Natural products (NPs) have always been of interest for the development of new medicines. Actinobacteria are known to be prolific producers of a wide range of bioactive NPs, and scientific evidence highlights their important protective role against CRC. This review is a holistic picture on actinobacter-derived cytotoxic compounds against CRC that provides a good perspective for drug development and design in near future. This review also describes the chemical structure of 232 NPs presenting anti-CRC activity with the being majority of quinones, lactones, alkaloids, peptides, and glycosides. The study reveals that most of these NPs are derived from marine actinobacteria followed by terrestrial and endophytic actinobacteria, respectively. They are predominantly produced by *Streptomyces*, *Micromonospors*, *Saliniospors* and *Actinomadura*, respectively, in which *Streptomyces,* as the predominant contributor generating over 76% of compounds exclusively. Besides it provides a valuable snapshot of the chemical structure-activity relationship of compounds, highlighting the presence or absence of some specific atoms and chemical units in the structure of compounds can greatly influence their biological activities. To the best of our knowledge, this is the first comprehensive review on natural actinobacterial compounds affecting different types of CRC. Our study reveals that the high diversity of actinobacterial strains and their NPs derivatives, described here provides a new perspective and direction for the production of new anti-CRC drugs and paves the way to innovation for drugs discovery in the future. The knowledge obtain from this review can help us to understand the pivotal application of actinobacteria in future drugs development.

## 1 Introduction

Colorectal cancer (CRC) is the third most common cancer globally with an annual rate of 10% of new cancer cases. Following lung cancer which is the deadliest cancer worldwide, CRC is the second deadliest cancer in the world, accounting for 9.4% of cancer deaths (Sung et al., 2021). The global burden, prevalence, and deaths from the disease are increasing and expects to reach around 60% by 2030 (Arnold et al., 2017). Environmental factors and genetic factors are involved in the occurrence of this complication. Heredity is a major cause of susceptibility to cancers, and in a case of CRC, it is estimated that 12%–35% of the risk is related to genetic factors. Based on the origin of genetic mutations, CRC is divided into three categories including sporadic (70%), inherited (5%) and familial (25%) (Peters et al., 2015; Mármol et al., 2017). Sporadics are heterogeneous, and different genes undergo point mutations. A specific sequence of mutations triggers with the APC gene following with mutations in the KRAS, TP53, and DCC genes are observed in approximately 70% of CRC cases (Fearon and Vogelstein, 1990). The hereditary group includes two categories namely polyposis and non-polyposis CRC (HNPCC). Two well-known examples for polyposis and HNPCC are familial adenomatous polyposis (FAP) and Lynch syndrome, respectively, in which occurs as a result of mutations in DNA repair genes (Lynch and de la Chapelle, 2003). Familial CRC, caused by hereditary mutations, is the type of cancer that neither its molecular mechanism is precisely known nor cannot be classified as a specific hereditary cancer (Stoffel and Kastrinos, 2014). The most common method of diagnosing, and determining the status of CRC in the clinic is the use of Tumor Node Metastasis (TNM) classification. However, today, due to the heterogeneity of cancer and in order to achieve personalized and targeted therapy, molecular classifications of cancer have been proposed which are indicators of genetic tumor heterogeneity (Turashvili and Brogi, 2017). Microsatellite instability (MSI), chromosomal instability (CIN) and epigenetic instability (CIMP) are the three major causes of genetic instability resulting CRC (Wang W. et al., 2019). Recently, based on such changes and other genetic factors and molecular pathways, another classification called Consensus Molecular Subtypes (CMSs) has been developed, which divides CRCs into four categories namely CMS 1–4 (Guinney et al., 2015). Currently, the most common treatments are surgery and chemotherapy. One of the first chemotherapeutic compounds used to control CRC was 5-fluorouracil (5-FU). This compound, along with leucoverin, due to its modulating effect, became the standard for the management of metastatic CRC (Piawah and Venook, 2019). Oxaliplatin, capsaicin, and topoisomerase inhibitors such as irinotecan are other compounds used in this field (de Gramont et al., 2000). Obvious adverse effects such as gastrointestinal and hematological toxicity as well as neurotoxicity when taking irinotecan and oxaliplatin, respectively, are not unexpected (Bailly, 2019; Guillaumot et al., 2019). Due to the existence of such side effects as well as the challenges associated with cancer metastasis, the use of targeted therapy has been considered. These therapies target the exact molecular pathway involved in causing cancer to inhibit the growth of cancer cells and limit their spread (Baudino, 2015). In this type of treatment, rather using conventional chemical therapies that have less noticeable effects and also have off-targets of healthy cells such as bone marrow and epithelial proliferating cells, compounds such as bevacizumb and cytoximb used to target Vascular endothelial growth factor (VEGF) and EGFR receptors, respectively. However, in such relatively new therapies, in addition to their side effects, even if confirmed in clinical trials, a hypothetical compound can be used and responsive only for a small proportion of patients with this complication due to the heterogeneity of CRC mutations (Piawah and Venook, 2019). Using combination therapy, combining conventional first-line therapies, ie chemotherapy, with natural compounds or targeted therapies such as immunotherapy, can be a good way to improve treatment methods and reduce unwanted adverse effects (Rejhová et al., 2018).

Natural products (NPs) are bioactive substances found in plants as well as fungi, bacteria and marine organisms. These compounds and their derivatives have been increasingly considered in the design of drugs including antibiotics and anti-cancer drugs owing to the variety of structural features and biological activities, as well as low toxicity and side effects. Natural compounds and their derivatives account for about one-third of FDA-approved drugs in the last 20 years (Rejhová et al., 2018; Thomford et al., 2018; Deng et al., 2020). These compounds are chemically included alkaloids, polyphenols, polysaccharides, terpenoids, carotenoids, unsaturated fatty acids and nitrogenous compounds. Natural compounds can affect biological pathways such as cell proliferation and cycle, cancer cell migration and invasion, apoptosis, autophagy and angiogenesis used to treat and alleviate cancer conditions (Rejhová et al., 2018; Huang et al., 2019).

Actinobacteria are prolific producers of a wide range of natural bioactive compounds due to their environmental diversity and consequently, their metabolic potential. They are generally gram-positive bacteria and one of the most diverse groups of microorganisms in nature that are scattered throughout the aquatic and terrestrial environments (Bahrami et al., 2022). In addition to the fact that about two-thirds of the known antibiotics used in clinical medicine are of actinobacterial origin, natural compounds of this origin are also used in the production of a range of anti-cancer, immunosuppressive, anti-parasitic and anti-viral agents (Barka et al., 2016; Van Bergeijk et al., 2020). By knowing that most biosynthetic gene clusters in *Streptomyces*, the largest genus of actinobacteria, are still silent under normal culture conditions, further studies on these microorganisms can be promising to explore more effective biological compounds in which indicates the unknown high potential of these bacteria in the production of secondary metabolites (Onaka, 2017). On the other hand, the presence of actinobacteria such as *bifidobacterium* in the intestinal microbiome has been shown to have a protective effect on CRC, which might be due to producing anticancer metabolites (Toumazi and Constantinou, 2020).

In this article, considering the current situation in the treatment of CRC and the needs to search and discover new effective compounds toward CRC, a comprehensive review of NPs affecting CRC cell lines isolated from actinobacteria has been conducted. The paper also describes the structure of over 230 compounds isolated and identified from actinobacteria in the past decades having anticancer properties based on their ecosystem and biological activity. To the best of our knowledge, this is the first comprehensive review on potential anti-CRC actinobacterial compounds, which can be an excellent guide for designing new drugs toward this disease, and introduce the unique biological potential of this extraordinary resource to the scientific community.

## 2 The Ecological Diversity of Actinobacteria and Their NPs Against Colorectal cancer

Actinobacteria, the vast and diverse branch of microorganisms, in which the genus *Streptomyces* alone, accounts for about 5% of all known bacterial species, are present in diverse ecosystems all over the world. Generally, they are placed in two categories, free and dependent on the host. The free species are widespread in both terrestrial and marine environments. The latter coexist in a wide variety of eukaryotes such as plants, insects, marine organisms, and even mammalian and human tissues such as skin, lungs and intestine (Lewin et al., 2016). Their natural bioactive compounds have various biological activities including antibacterial and anti-cancer, the well-known antitumor compound, doxorubicin, from a soil-derived *Streptomyces peucetius*, or the antitumor and antibacterial compound marinomycin, from a marine actinobacter species *Marinispora* spp. are examples (Arcamone et al., 1969; Kwon et al., 2006).

**CHART 1:**
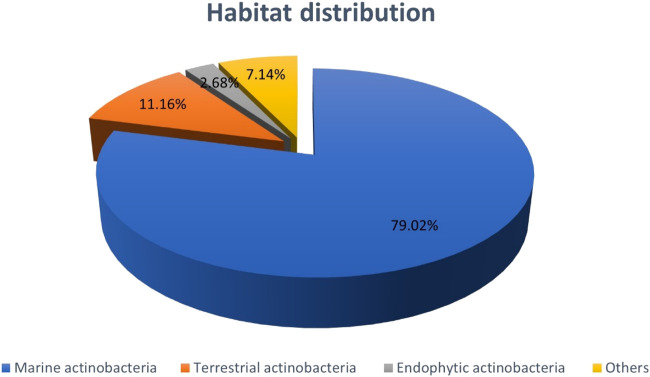
Distribution of NPs with anti-CRC properties produced by actinobacteria based on Bacterial habitat. The section entitled “Others” includes items such as Mutant actinobacteria, actinobacteria isolated from animals, salt ponds, sea sponge, and synthetics.

The proportion of natural bioactive compounds with anti-CRC properties derived from actinobacteria collected off different environments are presented in Chart 1. As the figure indicates the majority of compounds are produced by marine actinobacteria accounting for 79.02%. The reason, however, might be owing to the massive diversity of microorganisms in aquatic habitats which attracts more attentions of researchers into the marine ecosystem recently. This high volume of data without doubt highlights the great importance of marine actinobacteria in the production of anti-CRC compounds.

### 2.1 Natural Products From Marine Actinobacteria With Anti-CRC Activity

The significant diversity of NPs produced by marine actinobacteria is increasing day by day. Apart from the commercial aspect of compounds possessing medicinal properties, marine NPs have also gained special importance in the fields of biocatalysts and biological or microbial pesticides. It seems that the discovery rate of new chemical and bioactive compounds of marine-derived actinobacteria has been higher than its soil types for many years. These actinobacteria, which are often unable to grow *in vitro*, are found in marine habitats such as sea surface microlayer (SML), which contains 1 mm of sea surface, also seabed habitats containing marine sediments and a suitable place for microorganisms to coexist with microphones and macrofauna, fish surface, beach, coastal waters and even on the seabed (Bull et al., 2005; Maldonado et al., 2005; Ward and Bora, 2006). Among the different marine-derived microorganisms, actinomycetes are the largest producers of natural compounds, demonstrating their unparalleled potential. *Streptomyces* and *Micromonosporas* have a high potential for research on antitumor and growth inhibitors compounds (Khalifa et al., 2019). The following section refer to compounds isolated from actinobacteria having anti-CRC properties. The structure of NPs with anti-CRC properties from marine actinobacteria and their mechanism of actions and origins are summarized in Supplementary Table S1.

Salinosporamide A **(1)** (Marizomib, NPI-0052), having a fused γ-lactam β-lactone ring, and also its derivatives, salinosporamides D-J, have shown significant antitumor effects with inhibitory properties on the 20s proteosome (Feling et al., 2003; Jensen et al., 2007; Udwary et al., 2007). The inhibitory effect of these compounds was reported to be through the activation of NF-κB, inhibition of invasion and induction of apoptosis (Ahn et al., 2007). Salinosporamide A **(1)** has been isolated from marine actinobacteria *Salinospora tropica* CNB-392 (Feling et al., 2003; Jensen et al., 2007; Udwary et al., 2007). The anticancer effects of this compound on human colon carcinoma cell lines, namely HCT-116 (IC_50_ = 11 ng/ml) and HCC-2998, as well as a wide range of other tumors such as myeloma, non-hodgkin’s lymphoma, CLL and pancreatic carcinoma have been studied (Gulder and Moore, 2010; Sobolevskaia and Kuznetsova, 2010). This compound exhibits a high, stable and tolerable inhibitory effect on proteosome 20 on the xenograft model of colon cancer (Cusack et al., 2006). A combination therapy of compound (**1**) with common chemotherapy agents including 5FU, leucovorin, and oxaliplatin, was also reported to increase their anticancer effects significantly (Cusack et al., 2006). In 2003, few bioactive compounds with antibacterial and antitumor properties, were isolated from the marine derived *actinomadura* sp. M048 (Maskey et al., 2003). The compounds namely questiomycin A **(2)**, N-acetylquestiomycin A **(3)** and chandrananimycin A **(4)**, B **(5)** showed antitumor activity against several cell lines, including human colon carcinoma cell line, HT-29.

Three compounds of 3,6-disubstituted indoles isolated from the marine derived *Streptomyces strain* BL-49-58-005 were studied to evaluate their cytotoxicity effects against cancer cell lines. Indole number two (aldoxime) **(6)** showed activity against various cell lines, including LOVO and LOVO-DOX colon adenocarcinoma cell lines (Sánchez López et al., 2003). Arenamides A **(7)** and B **(8)** are two compounds with moderate anti-cancer activity against the HCT-116 cell line, isolated from the marine derived *Salinispora arenicola* CNT-088 (Asolkar et al., 2009). In addition, study on HEK cells, transfected by NF-κB-Luc/293 exhibited anti-inflammatory and chemoprevention effects of these compounds by blocking TNF induction activation and NF-κB inhibition.

In 2007, three macrolide polyketides compounds were isolated from the marine actinomycetes *Salinispora arenicola strain* CNR-005 (Williams et al., 2007). The compound arenicolide A **(9)** showed moderate cytotoxic activity against HCT-116 cell line with an IC_50_ value of 30 μg/ml. In 2004, a polyketide called aureoverticillactam **(10)** was isolated from the marine derived *S. aureo-verticillatus* NPS001583 (Mitchell et al., 2004). Study on the biological properties of this natural compound showed growth inhibitory properties on HT-29, Jurket Leukemia and B16F10 cell lines. In 2005, several natural chlorinated dihydroquinones compounds, in the form of a terpenoid/polyketide mix, were isolated from the marine derived *actinomycete strain* CNQ-525 collected from ocean sediments near La Jolla, California (Soria-Mercado et al., 2005). Dihydroquinones 1 **(11)**, 2 **(12)** and 4 **(13)**, showed cytotoxic effects against the HCT-116 cell line with the IC_50_ values of 2.4, 0.97, and 1.84 μg/ml, respectively. Cyanosporasides A **(14)** and B, structurally related to sporolides, were isolated from *S. pacifica strain* CNS103. The former one had poor toxicity effects against HCT-116 cell line, with an IC_50_ value of 30 μg/ml (Oh et al., 2006). Polyketides, from the family of manumycin compounds, called daryamides, were reported from *Streptomyces strain* CNQ-085. Their anti-cancer effects against HCT-116 cell line showed good results, especially for type A **(15)** with an IC_50_ value of 3.15 μg/ml (Asolkar et al., 2006).

Compounds isolated from *Streptomyces* sp. isolate B8652 and its derivatives such as trioxacarcins and gutingimycin have been shown to have antitumor effects against a variety of cancer cell lines, including HT-29. Four compounds called trioxacarcin A-D (**16–19**) were reported to have potent antitumor effects against various cell lines, including HT-29. On average, trioxacarcins A **(16)** had the highest effect among the rest, while gutingimycin **(20)** had the lowest toxicity (Maskey et al., 2004a; Maskey et al., 2004b).

The marine derived *Actinomadura* sp. BL-42-PO13-046, collected off the north coast of Spain, produces compound IB-00208 **(21)**, associated with angucyclines and other aromatic polyketides. The compound exhibits cytotoxic effects against human cancer cell lines, HT-29 (colon), SK-MEL-28 (melanoma) and A-549 (lung), as well as P-388 mouse leukemia (Malet-Cascón et al., 2003; Rodríguez et al., 2003). A macrolactone compound called IB-96212 **(22)**, was isolated from the actinobacterium *Micromonospora* sp. L-25-ES25-008 and its anti-cancer effects examined against various cell lines, including HT-29. However, these antitumor effects were reported to be four times more potent in P-388 mouse leukemia (Cañedo et al., 2000; Fernández-Chimeno et al., 2000). The antitumor effects of non-ribosomal peptides, lucentamycins, produced by *Nocardiopsis lucentensis* strain CNR-712, were tested against HCT-116 colon cancer cell line (Cho et al., 2007). Types A **(23)** and B **(24)** showed significant anti-cancer effects against the cell line with IC_50_ values of 0.20 and 11 µM, respectively.

**FIGURE 1:**
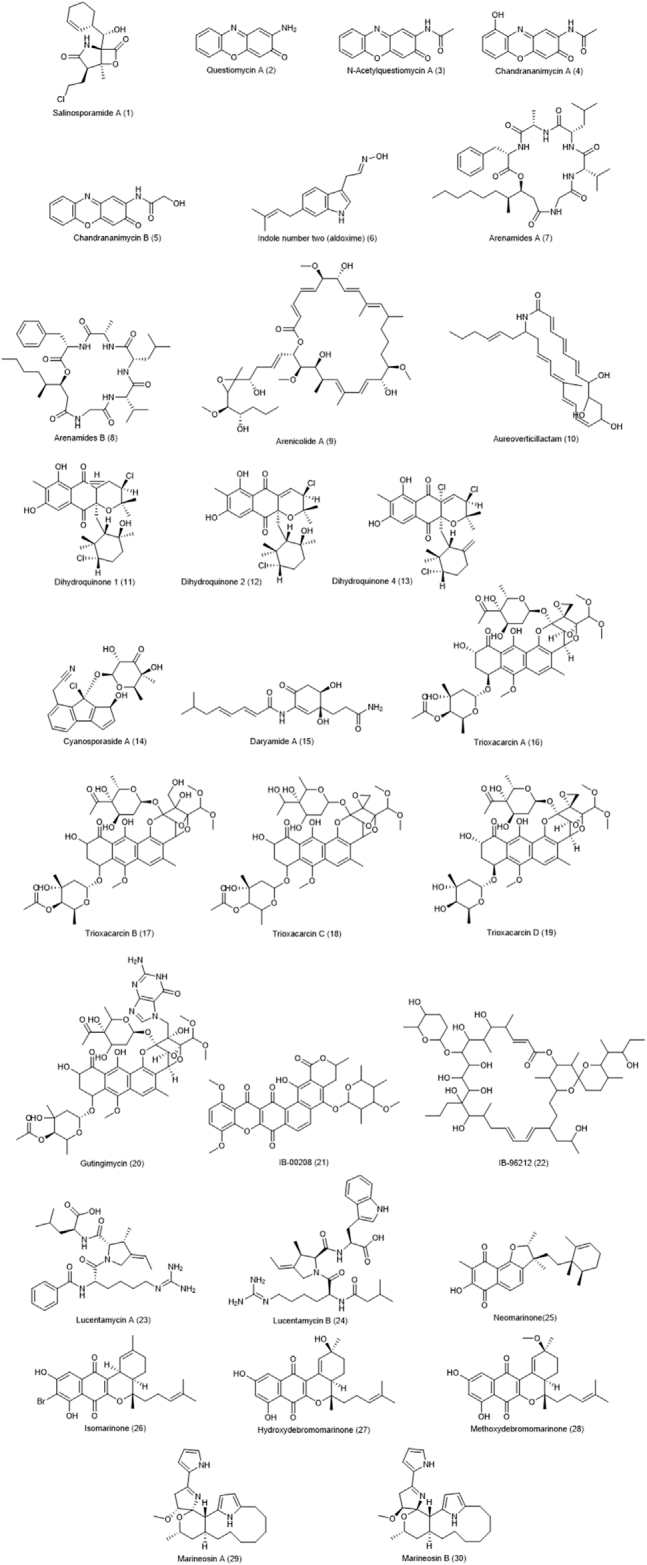
Chemical structures of NPs 1–30.

Marinones, including neomarinone **(25)**, isomarinone **(26)**, hydroxydebromomarinone **(27)**, and methoxydebromomarinone **(28)**, are compounds of a polyketide-terpenoid mix nature produced by *actinomycete isolate* CNH-099. These compounds exhibited moderate cytotoxicity against HCT-116 cell line with an IC_50_ value of 8 μg/ml (Hardt et al., 2000; Kalaitzis et al., 2003). *Streptomyces strain* CNQ-617, collected from marine sediments in La Jolla, California, was recognized as the producer of natural marineosins (Boonlarppradab et al., 2008). These compounds showed potential effects on different cancer cell lines, especially on HCT-116, with an IC_50_ value of 0.5 µM for type A **(29)** and 46 μM for type B **(30)**. The chemical structure of compounds 1–30 are presented in Figure 1.

Marmycins A **(31)** and B **(32)**, two bioactive natural compounds isolated from the marine actinobacterium *Streptomyces strain* CNH990, have potent anticancer effects on a variety of cancer cell lines (Martin et al., 2007). In the case of the HCT-116 cell line, marmycin A showed far more toxicity (18 folds) than the marmycin B. An average IC_50_ value of 0.022 μM was recorded for marmycin A against 12 human tumor cell lines, including colon cancer. However, the same studies showed weaker effects (mean 3.5 μM) for Marmycin B.

Piericidins were isolated from *Streptomyces* sp. YM14-060 in 2007 (Hayakawa et al., 2007a). Another study in the same year showed their antitumor effects on mouse glial cells transfected with the E1A adenovirus (RG-E1A-7) gene and Neuro-2a mouse neuroblastoma (Hayakawa et al., 2007b). Piericidins C7 and C8 lead to selective cytotoxicity on RG-E1A-7 cells and non-lethal growth inhibition on Neuro-2a cells. The cytotoxicity of various compounds was investigated on etoposide-resistant colon carcinoma cell line HT-29. The results showed that only piericidin A **(33)** among those compounds induces cytotoxicity in the cancer cell line via suppressing the accumulation of GRP78 protein (a drug resistance factor) (Hwang et al., 2008). *Streptomyces* sp. *strain* CNQ 593 was stated to produce non-cyclic and non-ribosomal chlorinated hexadepsipeptide compounds called piperazimycins (Miller et al., 2007). All of these compounds, including piperazimycin A **(34)**, show antitumor activity against colon (HCT-116), melanoma, prostate, and central nervous system cancer cell lines. The toxicity property of piperazimycin A against solid cancer lines was stronger three times those of piperazimycins B **(35)** and C **(36)**.

Staurosporines and their derivatives, are isolated from different species such as komodoquinones-producer *Streptomyces* sp. KS3 and *S. staurosporeus* AM-2282. Staurosporine, produced by *Micromonospora* sp. L-31-CLCO-002 and its natural analogues 4′-N-methyl-5′-hydroxystaurosporine **(37)** and 5′-hydroxystaurosporine **(38)**, were examined in terms of antitumor activity against various cancer cell lines namely lung, melanoma and HT-29 colon carcinoma. The latter two analog compounds had the highest activity against the HT-29 cell line (Omura et al., 1977; Hernández et al., 2000; Itoh et al., 2003). In a 2019 study, a total of 15 compounds of staurosporine derivatives, including 6 new compounds, was isolated from marine derived *Streptomyces* sp. NB-A13 in which 14 of them **(39–52)** had significant effects against SW-620 colon cancer cell lines (Zhou et al., 2019). Staurosporine derivatives No. 7 **(44)** and No. 14 **(51)** were reported to have the strongest cytotoxic function. In 2018, the structure and biological effects of staurosporine derivatives from *Streptomyces coelicolor* M1146 were investigated (Xiao et al., 2018). The compounds were produced by engineering the genes of the spc cluster, and expressing its heterologous. the toxicity of these derivatives was tested against various cancer cell lines. Among those, staurosporine M1 **(53)** showed activities against all of these cell lines, including HCT-116 (IC50 = 1.0M) and staurosporine M2 **(54)**, against HCT-116 cell lines (IC_50_ = 3.9 µM) and Huh 7.5 (IC_50_ = 2.7 µM).

In a 2007 study, the streptokordin **(55)** produced by *Streptomyces* sp. KORDI-3238 showed significant antitumor effects against HCT-15 colon cancer and other cancer cell lines such as MDA-MB-231, leukemia K-562, lung NCI-H23, etc. (Jeong et al., 2006). The antitumor activity of thiocoraline **(56),** produced by *Micromonospora (marina)* sp. L-13-ACM2-092 isolated from soft coral from the coast of Mozambique, Indian Ocean, has documented against HT-29 cell line. The compound, by stopping the cell cycle in G1 phase and slowing the progression of S phase of the cell cycle to G2/M, has antiproliferative effects on the LOVO and SW620 cell lines of the human colon with IC_50_ values of 15 and 500 nM, respectively. The inhibitory effect of compound is due to the inhibition of DNA polymerase α as well as its bilateral entry into the DNA strand (Romero et al., 1997; Erba et al., 1999). In 2014, *in vivo* studies found that the compound reduced tumorigenesis and proliferation of carcinoid tumor cells by inducing the activation of the notch pathway (Wyche et al., 2014). In 2015, the previously isolated natural compound thiochondrilline C from *Verrucisispora* sp. was synthesized in a special way and the antiproliferative effects of this compound and its derivatives were evaluated on the NCI 60 cancer cell panel (Vippila et al., 2015). Derivative No. 22 of thiochondrilline C **(57)** showed potent antiproliferative effects against CRC cell lines.

**FIGURE 2:**
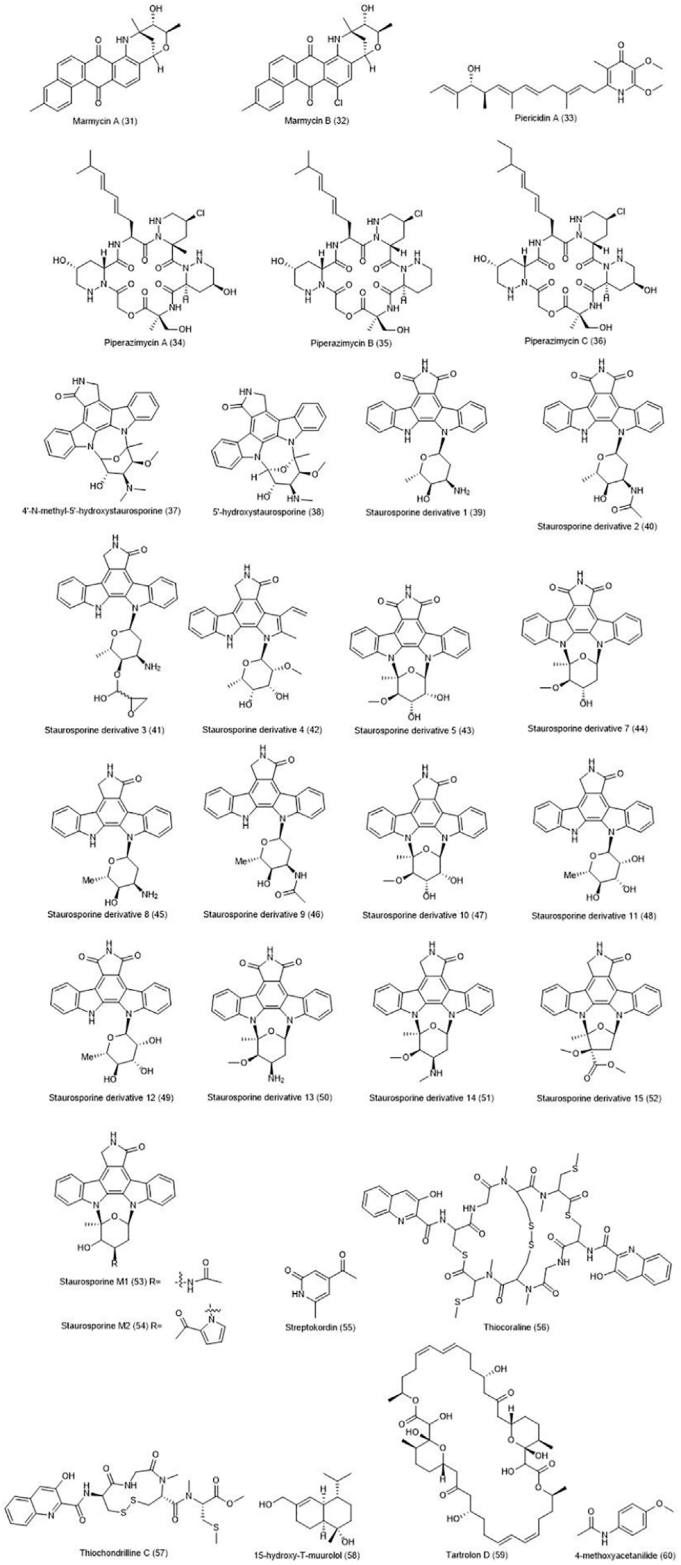
Chemical structures of NPs 31–60.

The eight t-muurolol compounds were isolated from marine actinobacterium *Streptomyces* strain M491 (Ding et al., 2009). Seven of the eight compounds were evaluated for their cytotoxicity, and MTT assay was performed on 37 human tumor cell lines, including CRC cell lines such as HCT-116 and HT-29. Except for the compound 15-hydroxy-T-muurolol **(58)**, with moderate toxicity effects, the rest of them left no noticeable effects on the t tested cell lines. Macrodiolide tartrolon D **(59)**, isolated from *Streptomyces* sp. MDG-04-17-069 has shown strong cytotoxic effects against human cancer cell lines including colon (HT-29), lung (A549) and breast (MDA-MB-231) (Pérez et al., 2009). 4-methoxyacetanilide **(60)** produced by marine *Streptomyces* sp. SCA29, was stated to inhibit α-glucosidase and α-amylase enzymes, and show antibacterial activity and cytotoxicity against various cancers, including HT-29 cell line (Siddharth and Vittal, 2019). The chemical structure of compounds 31–60 are illustrated in Figure 2.

A compound called levantilide C **(61)** has been identified from *Micromonospora* strain FIM07-0019 collected off the shallow coastal waters of Chile (Fei et al., 2013). The compound was reported to have a good antiproliferative activity against human colon cancer cell line SW620 (IC_50_ = 16.4 µM).

Cyanogrisides and their related analogues, produced by *Actinoalloteichus cyanogriseus* and its mutant species, can induce cytotoxic effects on cancer cell lines. Cyanogrisides F **(62)** and G **(63)** have shown cytotoxicity against HCT-116 and leukemia HL-60 cell lines (Fu et al., 2014). In 2014, three secondary metabolites amycofuran, amycolactam and amycocyclopiazonic acid, produced by *Amycolatopsis* sp., a marine sponge associated actinomycete, were examined for cytotoxic effects (Kwon et al., 2014). Amycolactam **(64)** had a significant effect on SNU638 gastric cancer and HCT-116 colon cancer cell line. The other two had little toxic effect.

Compounds called microbacterins A and B, produced by marine actinomycete *Microbacterium sediminis* spp. nov. YLB-01 exhibits significant cytotoxic effects against a panel of cancer cell lines. Microbacterin B **(65)** was stated to have cytotoxicity on all of tested cell lines, including HCT-8 human intestinal adenocarcinoma cell line (Liu et al., 2015). The isolated compounds from marine actinomycete *Pseudonocardia* sp. HS7 and its synthetic derivatives exerted a proliferative inhibitory effect on various cell lines (Ye et al., 2016). The anti-cancer effects of these compounds, called curvularin macrolides (1–5), **(66–70)** and their three synthetic acyl derivatives (5a-5c) **(71–73)** were measured using SRB Assay. They had significant effects on two human CRC lines HCT-15 and SW620.

Two polyhydroxyl macrolide lactone compounds, named PM100117 **(74)** and PM100118 **(75)**, isolated from the marine actinobacterium *Streptomyces caniferus* GUA-06-05-006A, lead to necrosis and significant antitumor effect on CRC HT-29, lung NSCLCA549 and breast MDA-MB-23 cancer cell lines, by altering the permeability and integrity of cell membranes (Pérez et al., 2016). Α-pyrone derivatives, called violapyrones B, C, H and I **(76–79)** isolated from marine actinomycetes associated with starfish, *Streptomyces* sp. 112CH148, from a region in Micronesia, have shown the effects of cytotoxicity against ten human cancer cell lines including HCT-15 and HCT-116 the Colon carcinoma cell lines (Shin et al., 2014). In 2012, the N- (2-hydroxyphenyl) -2-phenazinamine (NHP) **(80)** compound, produced by the marine *actinomycete species* BM-17, belonging to the genus *Nocardia dassonvillei*, was investigated (Gao et al., 2012). In addition to antifungal properties, the compound exhibited cytotoxic properties against HCT-116, cell line. In 2005, the compounds glyciapyrroles A, B, C from the marine derived *Streptomyces* sp. (NPS008187), collected off Alaskan marine sediments, were examined towards cancer cell lines (Macherla et al., 2005). Glaciapyrrole A **(81)**, showed good cytotoxic effects against colon cancer cell line HT-29.

**FIGURE 3:**
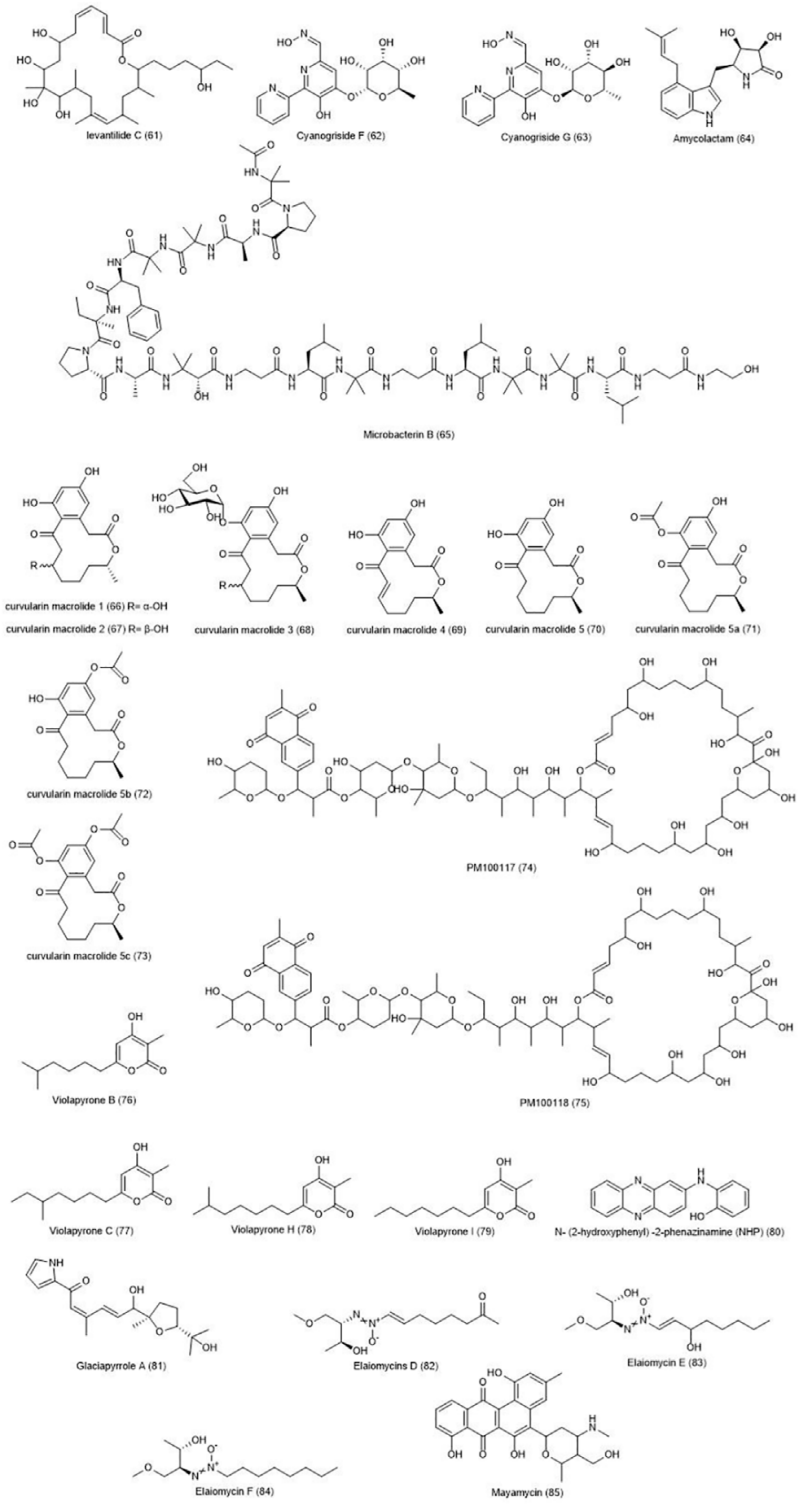
Chemical structures of NPs 61–85.

Elaiomycins D-F **(82–84)** with azoxy properties, were isolated from *Streptomyces* sp. strain HKI0708 (Ding et al., 2012). In addition to antibacterial effects, this compound exerted cytotoxic effects against 12 cell lines, including HT-29. Marine *Streptomyces* sp. strain HB202, isolated from the marine sponge *Halichondria panacea*, was examined as a producer of the mayamycin **(85)** compound (Schneemann et al., 2010). The compound has shown a significant toxicity against several bacteria, including antibiotic-resistant bacteria and eight cancer cell lines, including HT-29. Figure 3 demonstrates the chemical structure of compounds 61–85.

Marinopyrroles produced by the marine *Actinomycetes* CNQ-418, are classified as halometabolites, which contain halogen substituents in their chemical composition and have a variety of biological effects. Several marinopyrroles compounds were isolated from this species that showed antibacterial properties against methicillin-resistant species such as *Staphylococcus aureus* and selective cytotoxic properties against mammalian cancer cell lines namely leukemia such as K562 cells. Marinopyrroles A-F **(86–91)**, were demonstrated toxicity against HCT-116 cell line with IC_50_ values ranging from 1 to 9 μg/ml (Hughes et al., 2008; Doi et al., 2012). Another compound, Lodopyridone **(92)**, produced by *Saccharomonospora* CNQ-490, as a specific alkaloid, left relatively little toxic effect against HCT-116 cell line (IC_50_ = 3.6 µM) (Maloney et al., 2009).

Ammosamide halometabolites isolated from marine actinobacteria have a variety of toxic effects against the HCT-116 cell line. Ammosamides A **(93)** and B **(94)** were isolated from *Streptomyces* CNR-698, which showed a good toxicity with an IC_50_ value of 320 nM (Hughes et al., 2009a). Ammosamide D **(95)** was isolated from another species called *Streptomyces variabilis* SNA-020, which had a weaker cytotoxicity with an IC_50_ value of 3.2–4.9 µM (Pan et al., 2012). These compounds also had selective toxicity against other cancer cell lines. Cellular and molecular studies showed specific targeting of a member of the myosin family, which plays an important role in various mechanisms including the cell cycle (Hughes et al., 2009b).

Five compounds of nitropyrrolins A-E were isolated from the saline culture of the marine bacterium *S. malaysiensis* CNQ-509 (Kwon et al., 2010). They showed relatively poor antibacterial activity against tested organisms, but they showed acceptable cytotoxicity on the HCT-116 cell line. These results report IC_50_ value of 31 μM for type A **(96)** and B **(97)** and IC_50_ value of 5.7 μM for type D **(98)**. In 2013, the cytotoxicity of another halometabolite, chlorizidine **(99),** isolated from the marine *Streptomyces* strain CNH-287 was investigated against HCT-116 cell line, and reported to have a significant cytotoxicity with IC_50_ values of ranging from 3.2 to 4.9 µM (Alvarez-Mico et al., 2013). Terpenoid phenazines containing bromine, called marinocyanins A-F **(100–105)**, have been isolated from marine *actinomycetes MAR4* CNS-284 and CNY-960 (Asolkar et al., 2017). Marinocyanins A and B, in addition to their antifungal effects against amphotericin-resistant *Candida albicans*, along with marinocyanins C-F have shown cytotoxic effects towards HCT-116 with IC_50_ values of 0.049 and 0.029 µM, respectively. Two other halo metabolites including dionemycin **(106)** and 6-OMe-7′,7″-dichorochromopyrrolic acid **(107)** were reported by (Song et al., 2020). These two chlorinated bis-indole alkaloids were isolated from the marine *Streptomyces* sp. SCSIO 11791. Both compounds were exhibited significant cytotoxic effects on HCT-116 cell line with IC_50_ values of 4.3 and 13.1 μM, respectively, in addition to their antibacterial activities.

Napyradiomycins A – F **(108–113)** and B2-B4 **(114–116)**, isolated from *Streptomyces* strain CNQ-329 and CNH-070, were stated to have a weak cytotoxic effect against HCT-116 cell line (IC_50_ = 4.19–16 μM/ml) (Cheng et al., 2013; Farnaes et al., 2014). They also showed a mild to moderate inhibitory effect against methicillin-resistant MRSA *S.aureus*. They have a halogenated structure and a combination of terpene and polyctide, with a napthoquinone ring. Strain *Streptomyces* CNQ-525 produces various types of new compound of napyradiomycins called CNQ525.510B, CNQ525.538, CNQ525.554, and CNQ525.600 **(117–120)** as well as other known compounds including B1, B3, B4, A80915A **(121)**, A80915B **(122)**, A80915C **(123)**, A80915D **(124)**, CNQ525.512 **(125)**, and SF2415B3 **(126)**. In the study of the toxicity and inhibitory effect of these compounds against HCT-116 cell line, a range of results with IC_50_ values less than 1 µM to more than 100 µM was obtained.

**FIGURE 4:**
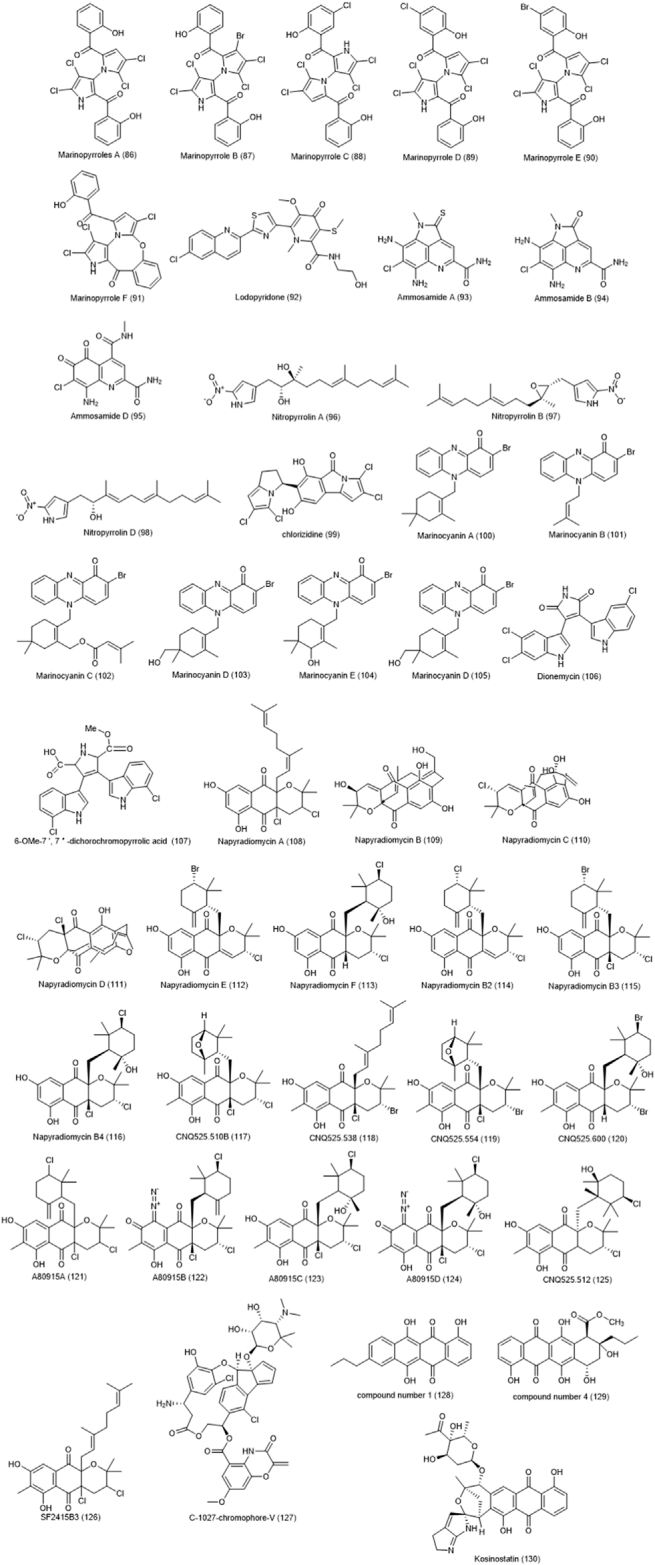
Chemical structures of NPs 86–130.

Actinobacterium *Streptomyces* strain ART 5, collected from the Arctic (eastern Siberia), were identified as a producer of fijiolide compounds A and B as well as C-1027-chromophore-V **(127)**, and C-1027-chromophore-III (Moon et al., 2014). Only two compounds, C-1027-chromophore V and C-1027-chromophore-III, were found to be effective against *Candida albicans*. Also, Chromophore-V was found to have a significant toxicity and antiproliferative activity against breast cancer MDA-MB231 and CRC HCT-116 cell lines with IC_50_ values of 0.9 and 2.7 µM, respectively. In 2012, four new anthracyclinone compounds were isolated from the marine derived *Micromonospora* sp. and their cytotoxicity effects on HCT-8 colon adenocarcinoma cell line were investigated (Sousa Tda et al., 2012). Among them compound number 1 (4,6,11-trihydroxy-9-propyltetracene-5,12-dione) **(128)** and compound number 4 (10β-carbomethoxy-7, 8,9,10-tetrahydro-4,6,7α 9α, 11-pentahydroxy-9-propyltetracene- 5,12-dione) **(129)** showed good cytotoxicity with IC_50_ values of 12.7 and 6.2 μM, respectively. In a 2002 study, kosinostatin **(130)** obtained from marine actinomycete *Micromonospora* sp. TP-A0468 showed a strong antibacterial effect against gram-positive bacteria and moderate effects against gram-negative bacteria and yeasts (Furumai et al., 2002). The cytotoxicity effect of this compound was tested against 39 cancer cell lines and exhibited significant impacts on most of test cell lines including HCT-116, HCT-15, HT-29, KM12, and HCC2998 with IC_50_ values average <1M. The compound inhibits human DNA topoisomerases I and IIα suggesting that the inhibitory effect of this compound occurs through interaction with DNA. The chemical structure of compounds 86 to 130 are shown in Figure 4.

In a 2001 study, lomaiviticins A **(131)** and B, isolated from *Micromonospora lomaivitiensis*, were examined against a wide range of human cancer lines including several colon cancer cell lines such as HCT15, CACO2, SW948, COLO205 (He et al., 2001). Lomaiviticin A was reported to have a strong toxicity effect against the tested cell lines with an average IC_50_ values of 0.01–98 ng/ml. lomaiviticins A and B also showed antibacterial effect against gram-positive pathogen species such as *S. aureus*. The compounds were introduced as potentially DNA-damaging compounds.

The actinomycete *Micromonospora aurantiaca* 110B, collected from the mangrove ecosystem in Fujian, China was reported to produce few compounds. Three isoflavonoid glycosides including daidzein-4′-(2-deoxy-α-l-fucopyranoside) **(132)**, daidzein-7-(2-deoxy-α-l-fucopyranoside) **(133)** and daidzein-4′,7-di-(2-deoxy-α-l-fucopyranoside) **(134)**, showed a good toxicity on A549 and HepG2 cancer cell lines, in addition to their appropriate effects on HCT-116 cell line (Wang RJ. et al., 2019). A new meroterpenoid, called actinoranone **(135)**, was isolated from a marine bacterium related to *Streptomyces*, and its cytotoxicity was examined against HCT-116 colon cancer cell line and reported to have a significant effect with LD_50_: 2.0 μg/ml (Nam et al., 2013). In 2019, three hydroxylated rhamnolipids, called dokdolipids A-C **(136–138)**, were found in marine actinomycete *Actinoalloteichus hymeniacidonis* 179DD-027, isolated from marine sediments on Dokdo Island, Republic of Korea (Choi et al., 2019). Their cytotoxicity was studied against various cancer cell lines, including HCT-15 and reported to have a good activity. It was reported that dokdolipid B had stronger activity than the other two compounds.

In 2019, the antibacterial and anticancer properties of Enediyne Congeners compounds produced by *Micromonospora yangpuensis* DSM 45577 isolated from cup-shaped sponge were examined. yangpumicins A, F, G **(139–141)** was reported to have antibacterial effects against gram-positive and negative pathogenic bacteria as well as a significant cytotoxicity against different human cancer cell lines (Wang Z. et al., 2019). All three compounds showed toxicity at about nM, against A549 lung cancer and Jurket lymphoma cell lines. However, in the case of human CRC cell lines Caco-2 and SKBR-3, the two compounds yangpumicins A and F were found to be 10 times more toxic than yangpumicins G.

Many NPs and metabolites were identified from marine actinomycetes *Saccharomonospora* sp. UR22 and *Dietzia* sp. UR66, isolated from the red sea sponge *Callyspongia siphonella*. (El-Hawary et al., 2018). Among them, the new compound saccharomonosporine A **(142)** and one of the induced metabolites **(143)** showed significant antiproliferative activity against human cancer cell lines HT-29 and HL60 (IC_50_ = 3.6, 3.7 µM respectively). Due to the inhibitory effect of these two compounds on Pim-1 kinase, it has been suggested that the inhibitory effect of these compounds on cancer cells is due to the induction of this function.

The structure and biological effects of 8 cyclizidine alkaloids, together with a known alkaloid, from a marine actinomycete called *Streptomyces* sp. HNA39 were defined. Among them, compound number 2 **(144)**, showed significant toxicity against HCT-116 with IC_50_ values of 8.3 µM and prostate cancer PC3 with IC_50_ values of 0.52 µM. In addition, the compound, along with compounds No. 5, 7 and 8, was stated to inhibit moderately ROCK2 protein kinase (Jiang et al., 2018a). Arenimycin **(145)** is a compound that has been shown to have a strong inhibitory effect on cell division with an IC_50_ value of 1.16 μg/ml against HCT-116 cell line. Arenimycin is an antibiotic consumed against rifampin- and methicillin-resistant *Staphylococcus aureus* produced by marine *actinomycete Salinispora* Arenicola (Asolkar et al., 2010).

New compounds called strepoxepinmycins A-D along with the well-known compound medermycin, have been identified from marine *Streptomyces* sp. XMA39 (Jiang et al., 2018b). They have new medermycin naphthoquinone’s chemical structure. It was reported all compounds possess the antibacterial effect on *E. coli* and MRSA species, as well as the antifungal effect on *Candida albicans*. Strepoxepinmycins C and D **(146–147)** were documented to possess good cytotoxic effects on colon cancer HCT-116 (IC_50_ values of 4.4 and 2.9 µM, respectively) cell line. Three new compounds called kendomycins B, C, D **(148–150)** were identified from the marine *Actinomycete Verrucosispora* sp. SCSIO 07399 (Zhang et al., 2019). In addition to their antibacterial activity against six species of gram-positive bacteria, these compounds also showed a suitable cytotoxic effect against several human cancer cell lines. Kendomycins B, C and D were suggested to have a good cytotoxicity against RKO, human colon cancer cell line, with IC_50_ values of 6.1, 3.8 and 36 µM, respectively. Four compounds. cyclo (Pro-Phe) **(151)**, cyclo (Pro-Ala) **(152)**, cyclo (Pro-Val) **(153)**, and cyclo (Pro-Leu) **(154)** with significant effects against HCT-116 cell line have been isolated from *Streptomyces nigra* sp. nov., collected from the rhizosphere soil of the *Avicennia marina* mangrove in China, which is closely related to *S. coerulescens* DSM 40146, *S. bellus* DSM 40185, and *S. coeruleorubidus* DSM 41172 (Chen C. et al., 2018). These compounds have a cytotoxic effect with an IC_50_ values of 32.3, 47.6, 67.2, and 92.6 µg/ml, respectively.

A unique furan-type compound **(155)** has been isolated *Streptomyces* sp. VN1 that was reported to have a good cytotoxic effect (IC_50_ = 123.7 µM) on various cell lines, including HCT-116 (Nguyen et al., 2020). This strain is isolated from the coastal region of Phu Yen Province (central Viet Nam). Petrocidin A **(156)** and 2,3-dihydroxybenzamide **(157)** were identified from *Streptomyces* sp*.* SBT348, isolated from a Marine Sponge (Cheng et al., 2017). These compounds exert their cytotoxic effect against HT-29 cell line by inhibiting the overexpression of microsomal prostaglandin E2 synthase-1 (IC_50_ = 5.3 and 3.8 μg/ml, respectively). In another study conducted in Turkey (Khan et al., 2019). Two compounds isolated from *Streptomyces cacaoi* 14CM034 have shown significant cytotoxic effects on the Caco-2 cell line through induction of apoptosis and inhibition of autophagy. Polyethers called K41 A **(158)** and compound **(159)** had IC_50_ values of 7.4 and 27.9 µM, respectively.

**FIGURE 5:**
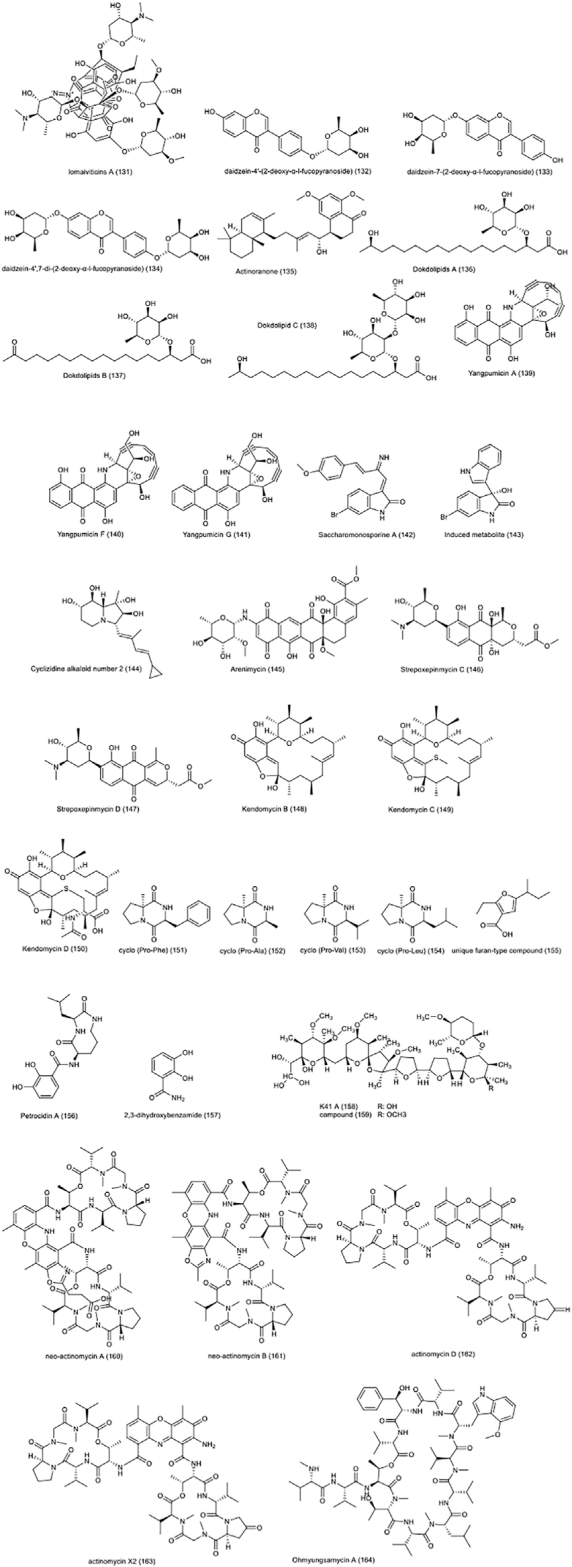
Chemical structures of NPs 131–164, and other NPs with anti-CRC properties from marine actinobacteria.

Two new compounds, neo-actinomycin A **(160)**, neo-actinomycin B **(161)**, along with actinomycin D **(162)**, and actinomycin X2 **(163)**, were isolated from *Streptomyces* sp. IMB094 (Wang et al., 2017). The cytotoxic effects of these compounds on HCT-116 cell line were determined with an IC_50_ value of 38.7, 339.1, 0.045 and, 0.0075 nM, respectively. Ohmyungsamycin A **(164)**, isolated from marine *Streptomyces* sp. SNJ042, was also reported to have a cytotoxic effect on the same CRC cell line with an IC_50_ value of 7.61 µM (Byun et al., 2020). The compound has been reported to work through caspase-mediated apoptosis, reducing the expression of Skp2 as an oncogenic factor, and increase the expression of p21 and p27, which are CDK inhibitors. The chemical structure of compounds 131 to 164 are presented in Figure 5.

### 2.2 Natural Products From Terrestrial Actinobacteria With Anti-CRC Activity

The soil ecosystem contains an estimated one billion bacterial cells per gram of soil, which includes tens of thousands of different taxa. (Wagg et al., 2014). Actinobacteria play a large role in this diversity. Actinomycetes, with a 90% share, are the most common actinobacteria isolated from this ecosystem (Suela Silva et al., 2013). Therefore, the soil ecosystem can be a valuable reservoir of NPs the structure of some NPs with anti-CRC properties, isolated from terrestrial actinobacteria and their name and mechanism of action and origin are summarized in Table 1.

**TABLE 1:** the structure of some NPs with anti-CRC properties from terrestrial actinobacteria and their mechanism of action and origin.

Bacteria	Origin of bacteria	Compound name	Chemical structure	Colorectal Cell line	Special property	country of origin	references
*Amycolatopsis* sp. IRD-009	Soil sample from Brazilian rainforest	Pradimicin-IRD (165)	Naphthacenequinone, Other hydrocarbones	HCT-116, TP53 −/−, HT-29, SW480 and Caco-2	Severe DNA damage, Apoptosis and cell cycle arrest	Iracemápolis/São Paulo, brazil	Almeida et al. (2019)
*Nonomuraea Endophytica* Strain GW58/450	Soil of an oak/beech mixed forest	Karamomycins A and C (166–167)	2-Naphthalen-2-Yl-Thiazole, Azoles	HT-29, HCT-116	—	Stadt Allendorf (Hessen, Germany)	Shaaban et al. (2019)
*Streptomyces* sp. 166	Clayey cold saline soil	Sekgranaticin, Granaticins A, B and Methyl granaticinate (168–171)	Polyketide-Naphthoquinone, Quinones	HCT-116 and P6C	—	altitude of 4547 m in Nima County (N31°52′, E87°1′), Naqu District, Tibet Autonomous Region, China	Lv et al. (2019)
*Streptomyces* sp. CC8-201	Soil of a remote karst cave	Xiakemycin A (172)	Naphthoquinone, Quinones	HCT-116	—	suburb of Chongqing city, China	Jiang et al. (2015)
*Streptomyces* sp. CPCC 204980	Soil sample	Cervinomycins C1-4 (173–176)	Polycyclic Xanthone, Xanthones	HCT-116	—	Mount Emei of Sichuan Province, China	Hu et al. (2020)
*Streptomyces* sp. HS-NF-780	Soil sample	9-Methylstreptimidone 2-Α-D-Glucopyranoside (177) and Hydroxyiso-9-methylstreptimidone (178)	Piperidines	HCT-116	—	Linyi, Shandong province, China	Zhao et al. (2019)
*Streptomyces* sp. KIB-H714	Soil sample	Actinomycin Z6 (179)	Peptides	SW480	—	Kunming Botany Garden, Kunming, China	Dong et al. (2019)
*Streptomyces* sp. KCB13F030	Soil sample	Ulleungoside (180)	Glycosides	SW480	Indoleamine 2,3-dioxygenase inhibition	depth of 5–10 cm from Ulleung Island, Korea	Son et al. (2017)
*Streptomyces* sp. *NEAU-L3*	Soil sample	Tetracenoquinocin A (181)	Aminoglycoside -Naphthacene, Glycosides	HCT-116	—	peak of Dayao Mountain of Guangxi province, China	Lu et al. (2017)
*Streptomyces* sp*.* KIB-H1318	Soil sample	A Phenoxazinone-related Alkaloid (182)	Alkaloids	SW480	—	Yuxi, Yunnan province, China	Yang et al. (2018)
*Streptomyces* sp. (172614)	Soil sample	Staurosporine analog (183)	Alkaloids	HCT-116	—	Jiulongjiangkou Mangrove, Fujian, China	Li et al. (2011)
*Streptomyces* sp. 211726	Mangrove rhizosphere soil of Heritiera globosa	Azalomycin F (4a) 2-ethylpentyl ester and Azalomycin F (5a) 2-ethylpentyl ester (184–185)	Macrolide, Lactones	HCT-116	—	Wenchang, China	Yuan et al. (2011)
*Soil-Derived Actinomadura Strain*	Rice field soil	Nonthmicin and Ecteinamycin (201–202)	Polyether/Polyketide, Lactones	mouse colon carcinoma cell line 26-L5	Anti-invasive effect	Thailand	Igarashi et al. (2017)
*Micromonospora* sp. Strain No. R385-2	Soil sample	Rakicidin A (219)	Lipopeptide, Peptides	HCT-8 and DLD-1	Angiogenesis and hypoxia	Andhra Pradesh, India	McBrien et al. (1995), Yamazaki et al. (2007)
*Streptomyces Hygroscopicus*	—	Sirolimus (Rapamycin) (224)	Macrocyclic Lactones	HT-29, SW620, and HCT-116	mTOR inhibition	Easter Island (also known as Rapa Nui), Chile	Li et al. (2014), Mussin et al. (2017)

In 2019, the pradimicin-IRD **(165)**, polycyclic compound was isolated from the terrestrial actinobacter *Amycolatopsis* sp. IRD-009 (Bauermeister et al., 2019). In addition to the strong antibacterial effect against *Streptococcus agalactiae*, *Pseudomonas aeruginosa* and *Staphylococcus aureus*, the compound was reported to possess a significant cytotoxic effect on HCT-116 cell line with IC_50_ value of 0.8 µM. Another study simultaneously was also examined the anti-cancer properties of the compound against colon cancer, various human colon cancer cell lines containing common mutations TP53 and KRAS. All of them, unlike the non-cancerous fibroblasts, showed significant sensitivity to the pradimicin-IRD compound. In the study of molecular cellular processes, induction of severe DNA damage, apoptosis and cell cycle arrest were observed. The regulation of p21 expression, independent of TP53, was observed in some cell lines such as HCT 116 TP53−/−, HT-29, SW480 and Caco-2 (Almeida et al., 2019). Karamomycins A-C isolated from soil actinomycete Nonomuraea endophytica strain GW58/450, and their biological effects was studied (Shaaban et al., 2019). Unlike karamomycin B, karamomycin A **(166)** showed non-selective toxicity against 36 human cancer cell lines, including two colon cancer cell lines HT-29 and HCT-116 with an average IC_50_ value of 6.8 µM. Karamomycin C **(167)** with slightly higher toxicity than karamomycin A (IC_50_ = 1.3 µM), showed good results.

Sekgranaticin **(168)**, granaticins A **(169)**, B (**170**) and methyl granaticinate (**171**) were isolated from the soil *Streptomyces* sp. 166, collected from the Tibetan highlands of China (Lv et al., 2019). The cytotoxicity of these compounds against lung, breast, colon carcinoma HCT-116 cell lines as well as human CRC stem cells P6C was evaluated by MTT method. All four compounds had a significant inhibitory effect on all tested cell lines. The strongest cytotoxicity effect on the two cell lines HCT-116 and P6C, was associated with granaticin B with IC_50_ values of 0.01 and 0.28 µM, respectively.

Xiakemycin A **(172)**, produced by soil *Streptomyces* sp. CC8-201, in addition to demonstrating a strong antibacterial effect against gram-positive bacteria, has shown a significant toxicity against various human cancer cell lines, including HCT-116 with an IC_50_ value of 0.59 µM (Jiang et al., 2015).

Polycyclic xanthones called cervinomycins C1-4 **(173–176)**, isolated from the soil *Streptomyces* sp. CPCC 204980, showed strong cytotoxicity effects against two cancer cell lines namely pancreatic BxPC-3 and colon HCT-116, with IC_50_ values in the range of 0.9–801.0 nM (Hu et al., 2020). Cervinomycin C3 showed the strongest effect on the HCT-116 cell line with an IC_50_ value of 0.9 nM. These compounds also showed potent antibacterial properties against gram-positive bacteria.

9-methylstreptimidone 2-α-D-glucopyranoside **(177)** and hydroxyiso-9-methylstreptimidone **(178)**, produced by the terrestrial *Streptomyces* sp. HS-NF-780, have shown a moderate cytotoxicity against lung, leukemia and colon cancer cell lines (Zhao et al., 2019). The compounds exhibited toxicity on the HCT-116 cell line with IC_50_ values of 34.83 μg/ml and 36.76 μg/ml, respectively.

A new member of the actinomycin family, called actinomycin Z6 **(179)**, identified from *Streptomyces* sp. KIB-H714. Unlike other Z- type actinomycens, this compound has an additional link between actinoyl chromophore and β-peptidolactone (Dong et al., 2019). The compound exhibited cytotoxicity on different cancer cell lines, including the human colon cancer line SW480 with an IC_50_ value of 1.57 µM. This compound had no antibacterial effects on the two test microorganisms namely *S. aureus* and *C. albicans*.

Three new glycoside compounds, including ulleungoside, 2-methylaminobenzoyl 6-deoxy-α-l-talopyranoside, and naphthomycinoside, were isolated from the terrestrial actinobacter species *Streptomyces* sp. KCB13F030 (Son et al., 2017). Ulleungoside **(180)** showed a significant cytotoxicity effect against several cancer cell lines including SW480 with an IC_50_ value of 9.3 µM. The compound also showed an inhibitory effect on the enzyme indoleamine 2,3-dioxygenase, involved in tryptophan metabolism.

The new compound, tetracenoquinocin A **(181)**, an anthracycline metabolite, was produced by the terrestrial *Streptomyces* sp. NEAU-L3, and its cytotoxicity was studied against three cancer cell lines of lung, liver and human colon in which showed a moderate cytotoxicity with an IC_50_ value of 20.82 μM on HCT-116 colon cancer cell line (Lu et al., 2017). Three new phenoxazinone-related alkaloids and two known compounds exfoliazone and viridobrunnine A, were identified in *Streptomyces* sp. KIB-H1318 (Yang et al., 2018). The second compound of phenoxazinone-related alkaloids **(182)** showed a weak toxicity effect against HeLa and SW480 cancer cell lines, with IC_50_ values of 36.8 and 37.8 µM, respectively.

**FIGURE 6:**
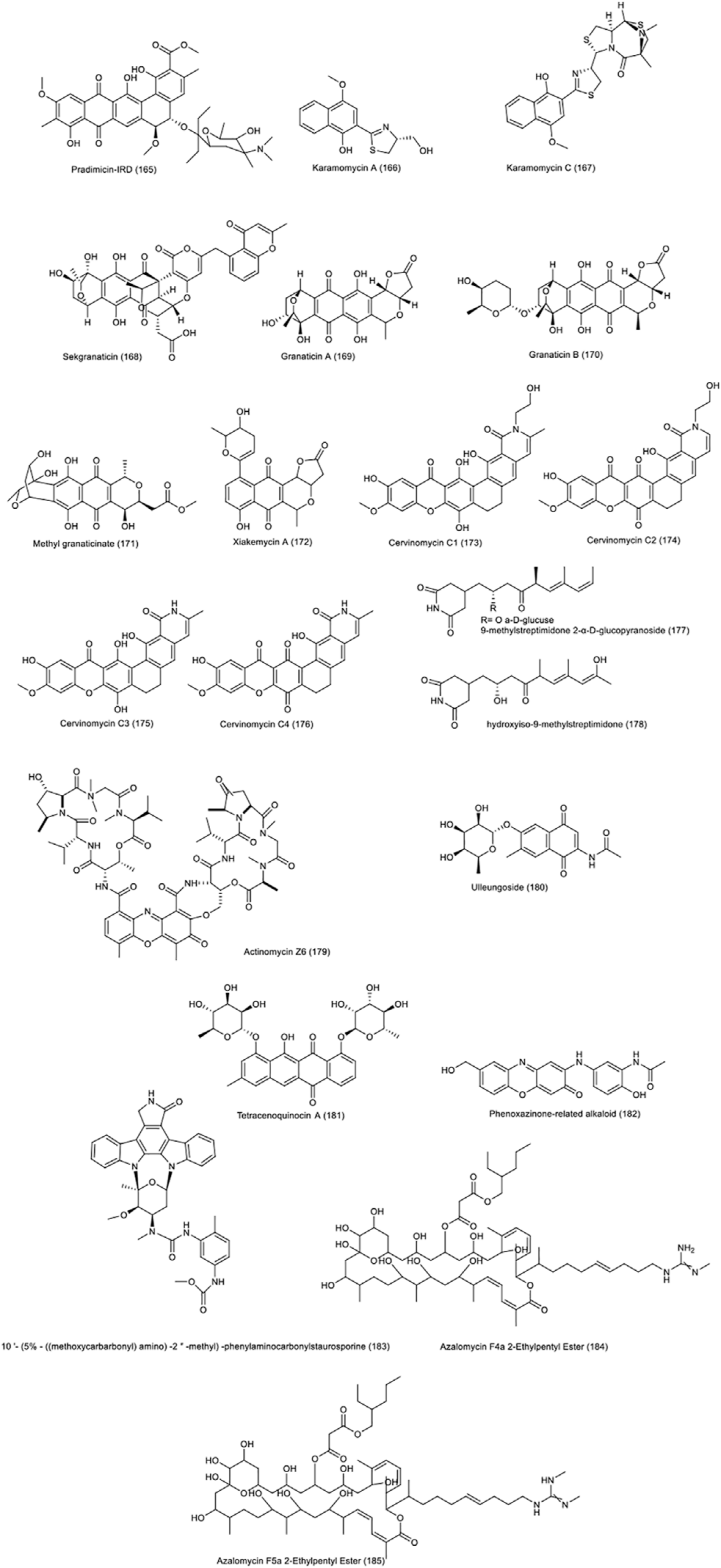
Chemical structure of NPs produced by terrestrial strains of actinobacteria.

The cytotoxicity of a staurosporine analog compound called 10′-(5%—((methoxycarbarbonyl)amino)-2″-methyl)-phenylaminocarbonylstaurosporine **(183),** isolated from mangrove soil derived actinomycetes *Streptomyces* sp. (172614) was studied against HCT-116. It was reported the compound showed a significant toxicity with an IC_50_ value of 0.37 µM (Li et al., 2011). The two compounds azalomycin F 2-ethylpentyl ester (4a) **(184)** and Azalomycin F 2-ethylpentyl ester (5a) **(185)** are produced by *Streptomyces* sp. 211726, isolated from mangrove rhizosphere soil (Yuan et al., 2011). Both compounds showed cytotoxic activity with IC_50_ values of 5.64 and 2.58 µg·ml^−1^ on the HCT-116 cell line, respectively, in addition to their antifungal activity toward *Candida albicans.* Figure 6 demonstrates the chemical structure of compounds 165 to 185 .

### 2.3 Natural Products From Endophytic Actinobacteria With Anti-CRC Activity

Endophytic actinobacteria are group of Gram-positive bacteria that live in symbiotic relationships inside plant tissues (Delbari et al., 2020; Abedinlou et al., 2022). This unique class of actinobacteria has shown high potential to produce a wide range of secondary compounds with diverse biological properties (Bahrami et al., 2022). The research on this category of bacteria has led to the discovery of over 200 new bioactive secondary metabolites having a diverse chemical structure with the being majority alkaloids, polyketides, flavonoids and terpenoids (Dinesh et al., 2017; Bernardi et al., 2019; Delbari et al., 2020). Table 2 summarizes the structure of some NPs with anti-CRC properties, isolated from terrestrial actinobacteria and their name and mechanism of action and origin.

**TABLE 2:** the structure of some NPs with anti-CRC properties from the endophytic actinobacteria and their mechanism of action and origin.

Bacteria	Origin of bacteria	Compound name	Chemical structure	Colorectal Cell line	Host plant	country of origin	References
*Streptomyces* sp. YIM66403	Healthy stem of the traditional Chinese medicinal plant Isodon eriocalyx	Misamycin (186)	Anthraquinone, Quinones	SW480	Root of Isodon eriocalyx	Xishuangbanna, Yunnan, China	Li et al. (2015)
*Streptomyces* sp. RLe8 *and Streptomyces cattleya* RLe 4	Brazilian Medicinal Plant Lychnophora ericoides Mart	2,3-dihydro-2,2-dimethyl-4 (1H) –quinazolinone and Nocardamine also called deferrioxamine (187–188)	Peptides and Quinazolines	HCT-8	Lychnophora ericoides	Brazilian bioma Cerrado, brazil	Conti et al. (2016)
*Streptomyces sp* KIB-H1083	Traditional Chinese medicinal plant Diaphasiastrum veitchii	Glucopiericidinol A (189)	Aminoglycoside, Glycosides	SW480	Diaphasiastrum veitchii	china	Shang et al. (2018)
*Micromonospora lupini Lupac* 08	Root nodules of Lupinus angustifolius	Lupinacidin A and B (190–191)	Anthraquinone, Quinones	Murine carcinoma colon 26-L5	Root nodules of Lupinus angustifolius	mid-west Spain	Igarashi et al. (2007)

An anthracycline called misamycin **(186)** is produced by the endophyte *Streptomyces* sp. YIM66403, isolated from the root of *Isodon eriocalyx* which is a traditional medicinal plant collected in China (Li et al., 2015). In addition to its antibiotic properties, the compound has shown a good cytotoxicity on several human cancer cell lines, including the CRC cell line SW4801 with an IC_50_ value of 9.75 μM.

2,3-dihydro-2,2-dimethyl-4 (1H) -quinazolinone **(187)** is a natural compound produced by the endophytic bacterium *Streptomyces sp* RLe8 isolated from the Brazilian plant *Lychnophora ericoides* that has shown significant cytotoxicity on the MDA-MB435, HCT-8, SF-295 and HL-60 cell lines with an IC_50_ value of 1.10 μg/ml on the HCT-8 colon cancer cell line (Conti et al., 2016). Another compound called nocardamine **(188)** produced by another endophytic species isolated from this plant called *Streptomyces cattleya* RLe 4 also showed significant cytotoxicity on these cells.

Two new compounds of glycosylated piericidins and four known compounds were identified from *Streptomyces* sp KIB-H1083, isolated from the traditional medicinal plant *Diaphasiastrum veitchii* (Shang et al., 2018). Among them, glucopiericidinol A **(189)** had a good cytotoxic effect on various cell lines such as HL-60, SMMC-7721, A-549, and MCF-7, although this compound was the weakest one against the SW480 CRC cell line with an IC_50_ value of 32.59 µM.

**FIGURE 7:**
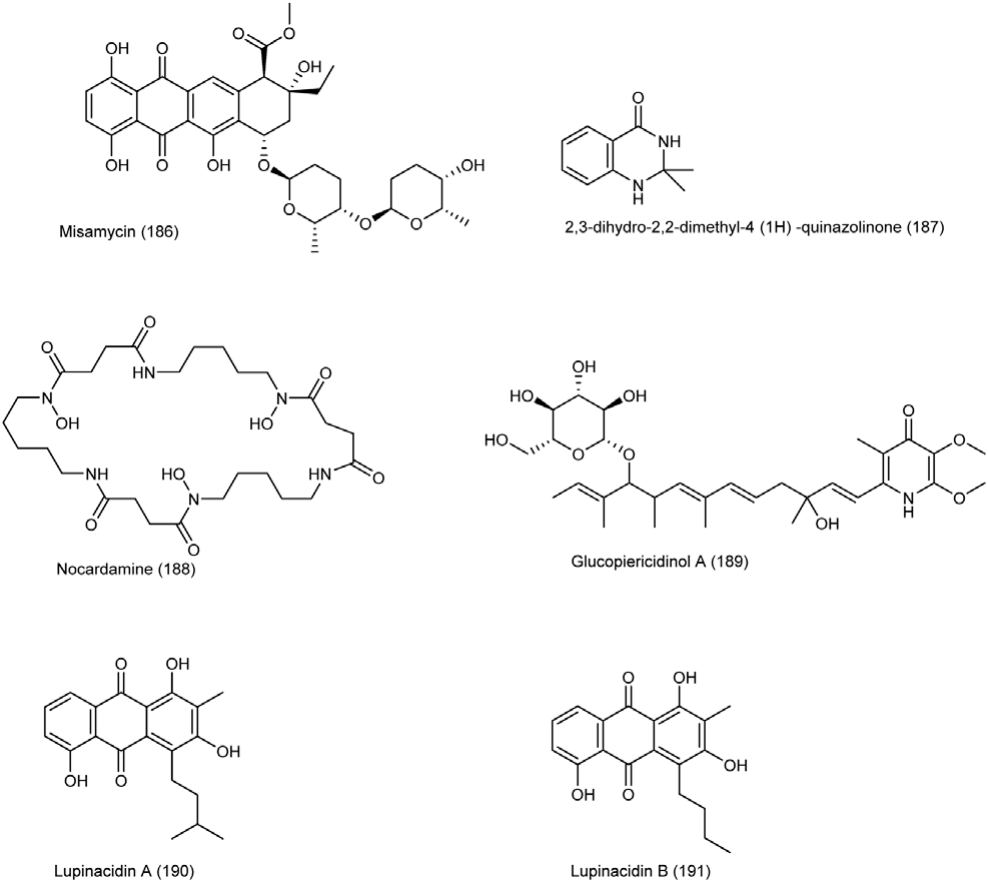
Chemical structure of NPs produced by endophytic strains of actinobacteria.

Lupinacidins A, B **(190–191)** from the endophytic species *Micromonospora lupini* Lupac 08 in non-toxic doses, was able to inhibit the invasive carcinoma cells, murine colon 26-L5, with IC_50_ values of 0.21 and 0.3 µM, respectively (Igarashi et al., 2007). The chemical structure of compounds 186 to 191 are illustrated in Figure 7.

### 2.4 Natural Products From Miscellaneous Actinobacteria With Anti-CRC Activity (From Unconventional Sources or Using Genome Mining)

A new derivative of the spectinabilin **(192)** compound, was identified from *Streptomyces* sp. 1H-GS5, isolated from ant (Liu et al., 2017). The cytotoxicity of this compound, along with another derivative of spectinabilin and the main compound spectinabilin as a positive control, gave good results against lung, liver and colon (HCT-116) cancer cell lines. The new spectinabilin derivative, showed a much stronger effect than the original compound, against the HCT-116 cell line, with an IC_50_ value of 12.8 μg/ml .

A total of 16 natural compounds of bafilomycins and odoriferous sesquiterpenoids were described from *Streptomyces albolongus,* isolated from the feces of the Asian *Elephas maximus* (Ding et al., 2016). Three compounds, 19-methoxybafilomycin C1 amide, bafilomycin C1 and bafilomycin C1 amide **(193–195)**, have significant cytotoxic effects on various cell lines, including BGC-823 human gastric carcinoma, Caco-2 colonic adenocarcinoma, H460 lung carcinoma, and SMMC-7721 hepatocellular carcinoma cell lines, with an IC_50_ range of 0.54–5.02 µM.

Retimycin A **(196)** was produced and identified from *Salinispora* sp. in genome-mining studies (Duncan et al., 2015). This compound showed a significant toxicity (IC_50_ < 0.076 μg/ml) on the HCT-116 cell line. Retimycin A, is a member of the quinomycin-like depsipeptide family. It was identified through the biosynthetic pathway of the rtm gene cluster using HR-MS/MS data, as compared to normal authentic.

Using genome mining, a peptide called curacozole **(197)** was isolated from *Streptomyces curacoi mutant* strain R25 (Kaweewan et al., 2019). The significant inhibitory effect was observed on the cell line of human osteosarcoma HOS (IC_50_ = 10.5 nM) and HCT-116 colon carcinoma (IC_50_ = 8.6 nM).

In 2015, salternamides A-D compounds were isolated from the *halophilic actinomycete* strain (no. HK10) found in salt ponds on Shinui Island in the Republic of Korea. The toxicity of these compounds on 6 human cancer cell lines, including colon, was investigated. The most significant toxicity was related to salternamide A **(198)** compound on HCT-116 colon cancer and SNU638 gastric cancer cell lines (IC_50_ = 0.96 µM) (Kim et al., 2015). The Subsequent studies have shown that this compound can inhibit hypoxia inducible factor (HIF-1α) accumulation in hypoxic conditions and suppress the upstream signaling such as PI3K/Akt/mTOR, p42/p44 MAPK, and STAT3. Stopping the cell cycle at G2/M and induction of apoptosis by this compound in HCT-116 cells were other results of this study (Bach et al., 2015).

**FIGURE 8:**
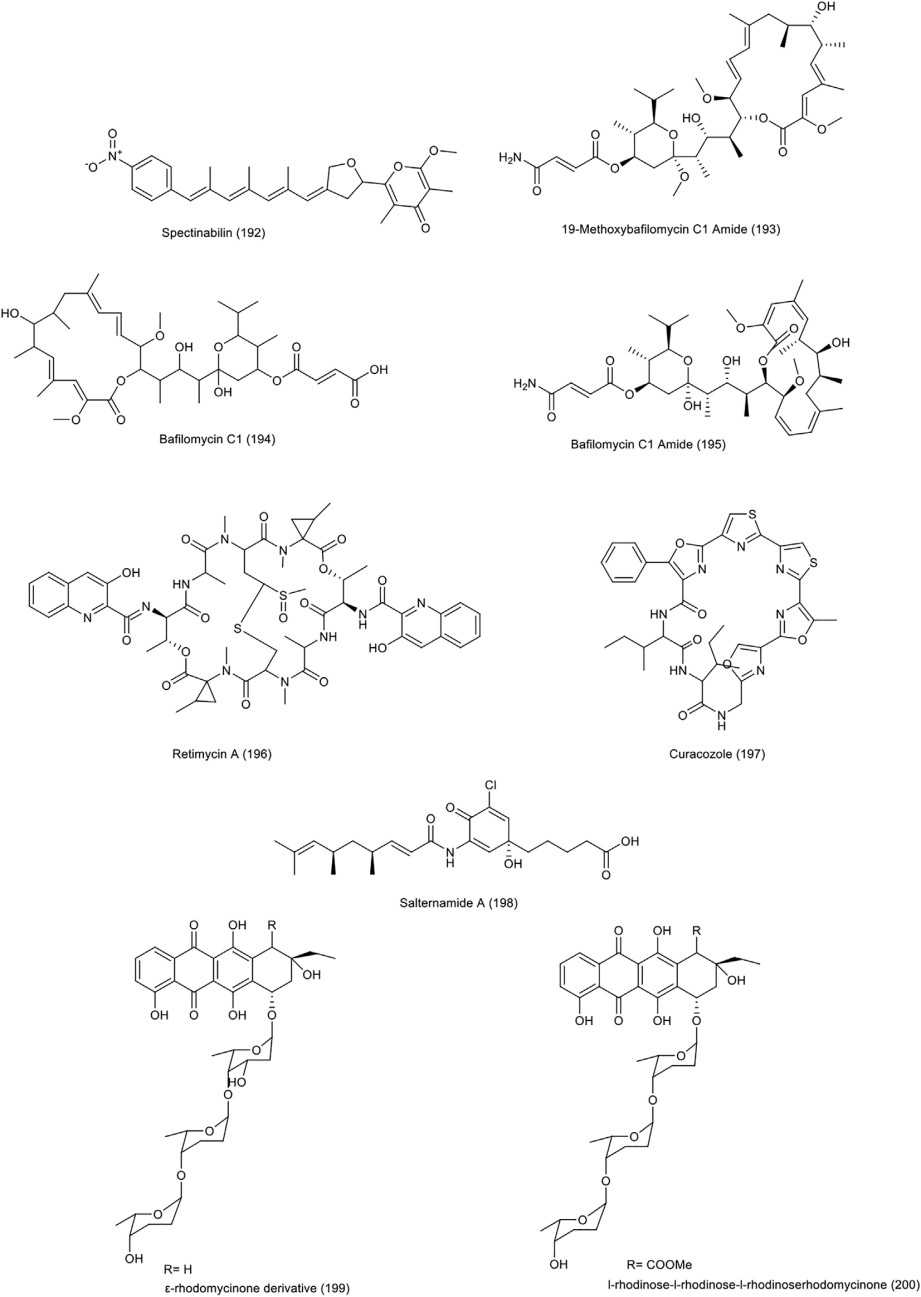
Chemical structure of NPs produced by miscellaneous actinobacteria.

In 2018, a mutant species *Streptomyces* sp. SCSIO 1666/17C4 was obtained by inserting cosmid p17C4 into the marine derived *Streptomyces* sp. SCSIO 1666 (Gui et al., 2018). Cosmid was the carrier of the biosynthetic gene l-rhodinose and the gene for glycosyltransferase12 anthracycline antibiotics, including three new derivatives of ε-rhodomycinone and nine new derivatives of β-rhodomycinone, were identified along with three previously known compounds from this species. Almost all compounds had a good cytotoxic effect against different cell lines. The strongest activity against HCT-116 cell line were related to compound number 4 of ε-rhodomycinone derivatives **(199)** and known compound l-rhodinose-l-rhodinose-l-rhodinoserhodomycinone **(200)**, with IC_50_ values of 0.3 and 0.2 µM, respectively. The chemical structure of compounds 192 to 200 are illustrated in Figure 8. The chemical composition of NPs with anti-CRC properties from miscellaneous actinobacteria and their mechanism of action and origin are outlined in Table 3.

**TABLE 3:** The structure of some NPs with anti-CRC properties from other actinobacteria and their mechanism of actions and origins.

Bacteria	Origin of Bacteria	Compound	Chemical structure	Colorectal Cell line	Country of Origin	Special property	references
*Streptomyces* sp. 1h-gs5	Head of ant (Camponotus japonicas Mayr)	A new derivative of the spectinabilin (192)	Pyrone, Pyranes	HCT-116	Northeast Agricultural University, Harbin of Heilongjiang province, China	—	Liu et al. (2017)
*Streptomyces albolongus*	Fresh fecal samples excreted by healthy adult *Elephas maximus*	19-methoxybafilomycin C1 amide, bafilomycin C1 and bafilomycin C1 amide (193–195)	Macrolide, Lactones	Caco-2	Xishuangbanna National Nature Reserve, Xishuangbanna, Yunnan Province, P. R. China	—	Ding et al. (2016)
*Salinispora* sp.	—	Retimycin A (196)	Quinomycin-type depsipeptide, Peptides	HCT-116	—	—	Duncan et al. (2015)
*Streptomyces curacoi* mutant strain r25	—	Curacozole (197)	Oxazole thiazole, Azoles	HCT-116	—	—	Kaweewan et al. (2019)
*Halophilic streptomyces* strain (no. Hk10)	Saltern	Salternamide A (198)	Polyene, Amides	HCT-116	Shinui Island in the Republic of Korea	Inhibition of HIF-1α accumulation, suppression of upstream signaling, Stopping the cell cycle at G2/M and induction of apoptosis	Bach et al. (2015)
*Streptomyces* sp. Scsio 1666/17c4	Recombinant	Number 4 of ε-rhodomycinone derivatives and -rhodinose-l-rhodinose-l-rhodinoserhodomycinone (199–200)	Anthraquinone, Quinones	HCT-116	-	-	Gui et al. (2018)
*Streptomyces violascens*	Fresh fecal samples excreted by healthy adult Ailuropoda melanoleuca (giant panda)	Fusicomycin A, fusicomycin B and isofusicomycin A (203–205)	Fusicoccane diterpene, Terpenes	HCT-116	Yunnan Wild Animal Park, Kunming, Yunnan Province, People’s Republic of China	Anti-adhesion, anti-invasion and migration effects	Zheng et al. (2017)
*Streptomyces* sp. Gku 220	Marine sponge sample	Rakicidin F and C (206–207)	Cyclic depsipeptide, Peptides	murine carcinoma colon 26-L5	Andaman sea, Ranong, Thailand	Anti-invasion	Kitani et al. (2017)
Nanoparticle conjugated *-Streptomyces* sp. Js520	Pristine sediment from the cave	Undecylprodigiosin (211)	Pyrrole, Azoles	HCT-116	mountain Miroc in Serbia	Apoptosis	Nikodinovic-Runic et al. (2014)
*Synthesized –Actinomycete* z2039-2	Sea Earth	K252c derivative (212)	Indolopyrazolocarbazole, Indoles	HCT-116	coast of Qingdao, China	Apoptosis	Liu et al. (2007), Esvan et al. (2016)

### 2.5 Actinobacteria as Human Gastrointestinal Tract (Gut) Microbiome

In addition to producing effective bioactive secondary metabolites, actinobacteria have been reported to be one of the four main phyla of the gut microbiota, revealing another aspect of their relationship. It is evident that one of the most associated risk factors for precancerous and cancerous intestine conditions is a change in the gut microbiome and microbiota dysbiosis. The variation in the gut microbiome may generate CRC by destructing host DNA, creating and maintaining a pro-inflammatory condition, and influencing host immune responses (Kværner et al., 2021). Evidence has corroborated that the alteration in the gut microbiome in the early stages of CRC may be employed to diagnose and detect individuals at risk of presenting colorectal adenoma. These changes can also affect the effectiveness of conventional therapies such as radiotherapy and chemotherapy (Zhou et al., 2021). Therefore, several studies, have been conducted to determine specific microbial patterns or significant changes in the intestinal microbial community of patients and healthy individuals, using the molecular study of faecal microbiota, in order to find a suitable biomarker for CRC .

Actinobacteria are reported to play a critical role in the maintenance of gut homeostasis. A study compared healthy individuals (H) and groups with hyperplastic polyps (HP), low-risk adenomas (LRA), high-risk adenomas (HRA), adenocarcinomas (ADK), and the last group included patients with ADK who received chemotherapy or radiotherapy (ADK-T) (Mori et al., 2018). Bacterial flora analysis showed that the most common phyla in healthy people are *Firmicutes, Bacteroidetes, Actinobacteria* and *Proteobacteria*. However, patients who are dealing with ADK, *Firmicutes* and *Actinobacteria* are significantly decreased, which can, in addition to the *Lachnospiraceae* family, be considered as good specific biomarkers for H, LRA and HP individuals. Also, in people with preneoplastic/neoplastic lesions, in general, a decrease in the *Firmicutes/Bacteroidetes* ratio can be considered as a marker for intestinal dysbiosis. Comparison between healthy people, people with inflammatory bowel disease (IBD) and those suffering from CRC have shown that the frequencies of the three phyla*, Firmicutes*, *Bacteroidetes*, and *Actinobacteria*, involved in metabolic pathways, are significantly different among them (Ma et al., 2021). Phyla *Firmicutes* and *Actinobacteria* were the main differential bacteria in the healthy group and phyla *Fusobacteria*, *Proteobacteria*, and *Verrucomicrobia* were reported as differential bacteria in the CRC patient group.

In a cohort study between healthy young people (below 30 years old), healthy old group (over 60 years old) and CRC patients, several bacterial species were reported to be distributed inequality in these groups (Zhang et al., 2021). *Clostridia, Fusobacteria, Actinobacteria*, and *Bifidobacterium* were introduced as potential biomarkers in CRC patients of different ages. *Actinobacteria* at the phylum and class level were more abundant in the older group than in the younger volunteers. Interestingly, the abovementioned frequency was lower in CRC patients than in both healthy groups.

A similar study compared the microbiome of CRC and adjacent normal mucosa tissues by which a significant reduction in the relative abundance of *Firmicutes* and *Actinobacteria* was observed in tumor tissue as compared to adjacent healthy tissues (Gao et al., 2017). In addition, the presence of *Villanelle, Firmicutes*, and *Actinobacteria* (family *Bifidobacteriales*) were reported with the greater frequency in the group without adenoma as compared to the group of patients having adenoma, which is a common precursor for CRC, confirmed the previous results (Hale et al., 2017).

Determining the frequency and specificity of microbiome in CRC patients and the microorganisms involved in the development and progression of this disease can be used as a screening marker to predict and evaluate precancerous evidence based on the present ratio of the protective bacteria to its harmful types. As documented in many studies, phyla such as *Actinobacteria* and *Firmicutes* have been implicated as protective microbiomes against CRC (Hale et al., 2017; Mori et al., 2018; Zhang et al., 2021).

#### 2.5.1 Perspectives on the Relationship Between Gut Microbiome and Cancer

Actinobacteria, these amazing microorganisms that produce a variety of bioactive secondary metabolites, have not withheld their gift from the human body due to their diversity in various habitats. The isolates from intestinal microbiomes in addition to producing the secondary metabolites and their amazing potency of the anticancer effects, these bacteria were introduced as cancer killers. In this view, it is possible that these bacteria, of which actinobacteria constitute a significant part, are enabled not only to prevent CRC locally, but also to inhibit spreading cancer in other organs systemically in the very early stages by producing anti-cancer compounds (Zhou et al., 2017). It has been suggested the difference between the function of these bacteria in preventing cancer and the production of secondary compounds in individuals can be due to the genetic predisposition of individuals and environmental factors such as diet and the amount of antimicrobial use during their lifetime.

A study presented that a significant difference in the abundance of several taxa between adenoma and non-adenoma patients, led to the perspective that the accumulation of sugar, protein and lipids metabolites as well as increased bile acid production can provide environmental conditions in favor of the growth of bile acid-resistant microbes, and these microbes, in turn, provide the conditions for the development of adenomas and CRC, by generating substances such as inflammatory and genotoxic metabolites (Hale et al., 2017).

## 3 Potential Biological Mechanisms and Function of NPs Against Colorectal Cancer

### 3.1 Migration and Invasion

Nonthmicin **(201)** and ecteinamycin **(202)** compounds produced by soil-derived *Actinomadura strain*, in addition to exhibiting strong antibacterial effects against gram-positive bacteria, have shown anti-invasive effect against mouse colon carcinoma cell line 26-L5 (Igarashi et al., 2017). These two compounds, with IC_50_ values of 0.017 and 0.15 µM, respectively, inhibit the invasion of cancer cells into the extracellular matrix; however, they did not show cytotoxicity.

Six new fusicoccane-type diterpenoids were identified from *Streptomyces violascens,* isolated from the feces of the panda *Ailuropoda melanoleuca* (Zheng et al., 2017). Fusicomycin A **(203**, fusicomycin B **(204** and isofusicomycin A **(205)** showed good cytotoxicity against 5 human cancer cell lines including colon carcinoma HCT-116 with IC_50_ values of 6.7, 5.8 and 8.0 µM, respectively. In the study of the effect of these compounds on adhesion, invasion and migration of SMMC7721 hepatocarcinoma cells, fusicomycin B showed a significant anti-adhesion effect and also reduced the number of migrating cells and therefore the anti-invasion and migration effect was recorded in these studies.

Rakicidin F **(206)**, C **(207)**, produced by *Streptomyces* sp. GKU 220 isolated from sea sponge were examined for biological activity (Kitani et al., 2017). Rakicidin F, in addition to its growth inhibitory effect towards *B. subtilis* and *E. coli*, showed higher cytotoxicity than rakicidin C. While treatment of murine carcinoma colon 26-L5 cells with rakicidin C compound at a dose of 1.25 µg, inhibited the invasion of these cells by 30% without causing toxicity.

Androsamide **(208)**, isolated from marine actinomycete *Nocardiopsis,* strain CNT-189 was reported to exhibit antitumor activity against colorectal cell lines in a variety of ways. The effect of this compound on three human cell lines adenocarcinoma (AGS), HCT-116 and Caco-2 showed cytotoxicity with IC_50_ values of 18, 21 and 13 µM, respectively (Lee et al., 2020). Further studies on Caco2 CRC cell line, the androsamide showed the inhibition of migration and invasion of these cancer cells. These results were confirmed in further studies on the expression of related factors such as Snail, Slug, Twist, ZEB1, and ZEB2 and markers such as E-cadherin, N-cadherin, and vimentin at the mRNA and protein levels. Finally, strong suppression of Caco2 cells motility by this compound was reported.

### 3.2 Apoptosis

**FIGURE 9:**
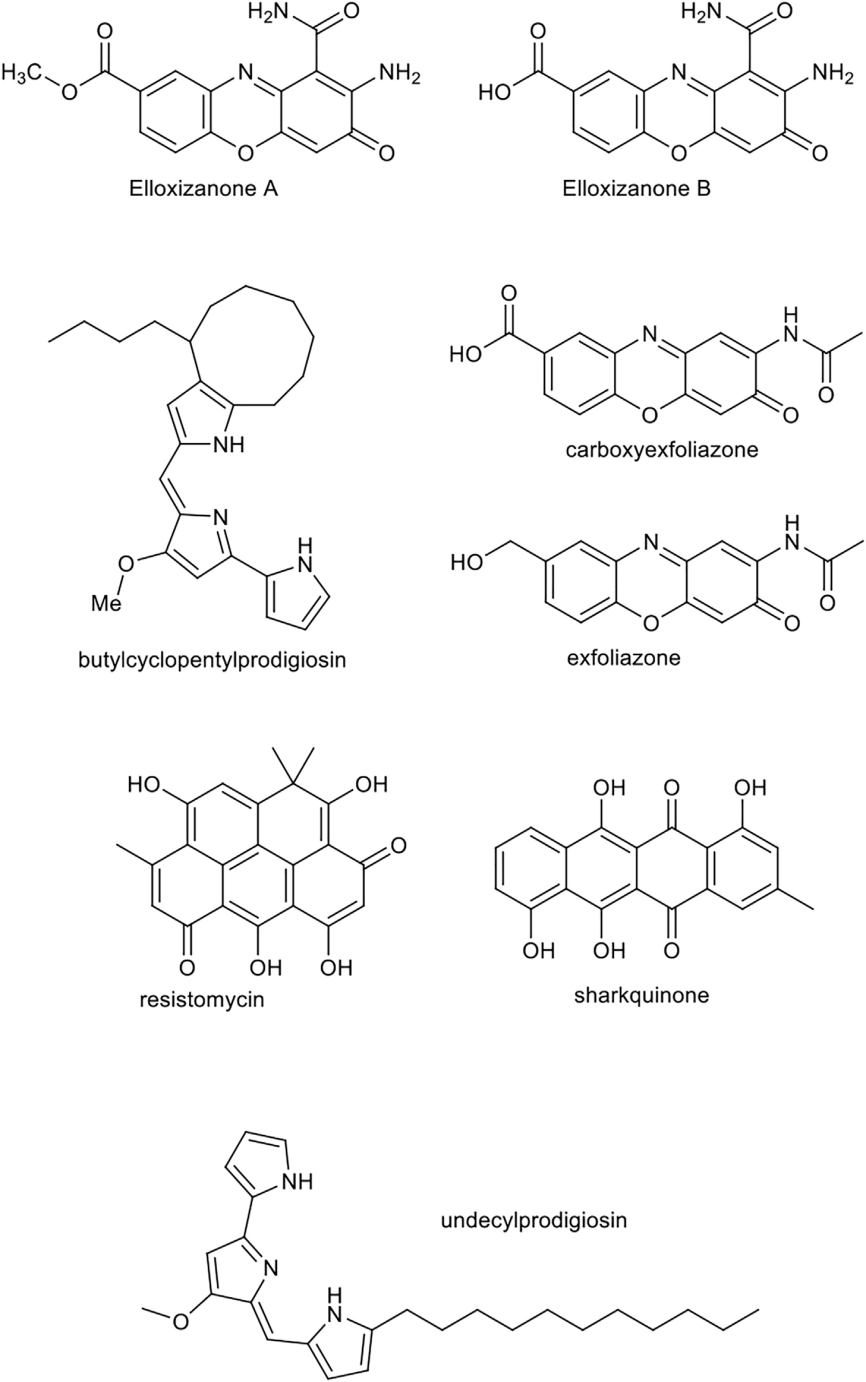
Chemical structure of sharkquinone, resistomycin, undecylprodigiosin, butylcyclopentylprodigiosin, elloxizanones A and B, carboxyexfoliazone and exfoliazone.

It was reported that natural compounds produced by distinct marine actinomycetes namely sharkquinone, resistomycin, undecylprodigiosin, butylcyclopentylprodigiosin, elloxizanones A and B, carboxyexfoliazone and exfoliazone, were able to initiate cell death via internal apoptosis pathway and susceptibility of cancerous to tumor necrosis factor-related apoptosis-inducing ligand (TRAIL). The chemical structure of these compounds is displayed in Figure 9. Evident has showed that these compounds are able to reduce the expression of X-linked inhibitor of apoptosis protein (XIAP) and survivin factors, which are members of the inhibitor-of-apoptosis protein (IAP) family recognized for their inhibitory effects on caspase (Elmallah et al., 2020). Thereby they can stimulate the apoptosis of human cancer cells. The use of these compounds could possibly help to fight the resistant cancer cells to TRAIL and its derivatives. A study suggested the above-mentioned pure compounds induce death in breast cancer MDA-MB-231, Jurkat leukemia and HCT-116 colon carcinoma cell lines, and their combination uses with TRAIL, synergistically increase its apoptotic properties. These compounds show hallmarks of apoptosis including activation of pro-caspase -10, -8, -9 and -3 and consequently the increase in lamins A/C and cleavage of PARP in cancer cells.

Two compounds, echinosporin **(209)** and 7-deoxyechinosporin **(210)**, produced by the marine derived *S. albogriseolus A2002*, collected in Jiaozhou Bay, China, were documented to have replication inhibitory effects on different types of cancer (Cui et al., 2007). Echinosporin with relatively strong effects and 7-deoxyechinosporin with weaker effects show cytotoxicity on HCT-15 colon carcinoma cell line. They induce apoptosis and cell cycle arrest generally in the G2/M phase, which are promising.

Metacycloprodigiosin and undecylprodigiosin from the prodigiosin family, were isolated from the marine actinomycete *Saccharopolyspora* sp. nov which showed cytotoxicity against five cancer cell lines namely mouse lymphoma (P388), human leukemia (HL60), human lung carcinoma (A549 and SPCA4) and liver carcinoma (BEL-7402) (Liu et al., 2005). The compound undecylprodigiosin **(211)**, was isolated from the non-marine *Streptomyces* sp. JS520. It was conjugated to gold nanoparticles and showed lethal effects through induction of apoptosis on melanoma A375, lung carcinoma A549, breast cancer MCF-7 and colon HCT-116 (Nikodinovic-Runic et al., 2014).

The compound k252c, produced by strain *actinomycete* Z2039-2, was documented to induce apoptosis in the leukemia cell line K562 (Liu et al., 2007). In 2016, a derivative of this compound (indolopyrazolocarbazole) **(212** was synthesized by replacing the lactam ring with the pyrazole component, which showed moderate lethal effects against leukemia and colon carcinoma K562 and HCT-116 (Esvan et al., 2016).

Manumycin A **(213)**, a compound with the inhibitory activity of Ras farnesyl transfrase, from the marine *Streptomyces* sp. M045 inhibits the growth of Ki-ras-activated mouse fibrosarcoma (Hara et al., 1993; Sattler et al., 1998). A 2000 study demonstrated the inhibition of the signaling pathway of manumycin by inducing apoptosis in the COLO320-DM colon cancer cell line. Finally, they reported that the inhibition of p21 ras processing and signaling was associated with the inhibition of proliferation and induction of apoptosis, and that the presence of the ras mutant gene was not required to exhibit these effects (Di Paolo et al., 2000). However, it was later found that the cytotoxic activity of this compound was independent to the RAS pathway and they reported ROS induction, especially O2−, in cells treated with this compound and were identified as effective compounds on thioredoxin reductase (Tuladhar et al., 2019).

**FIGURE 10:**
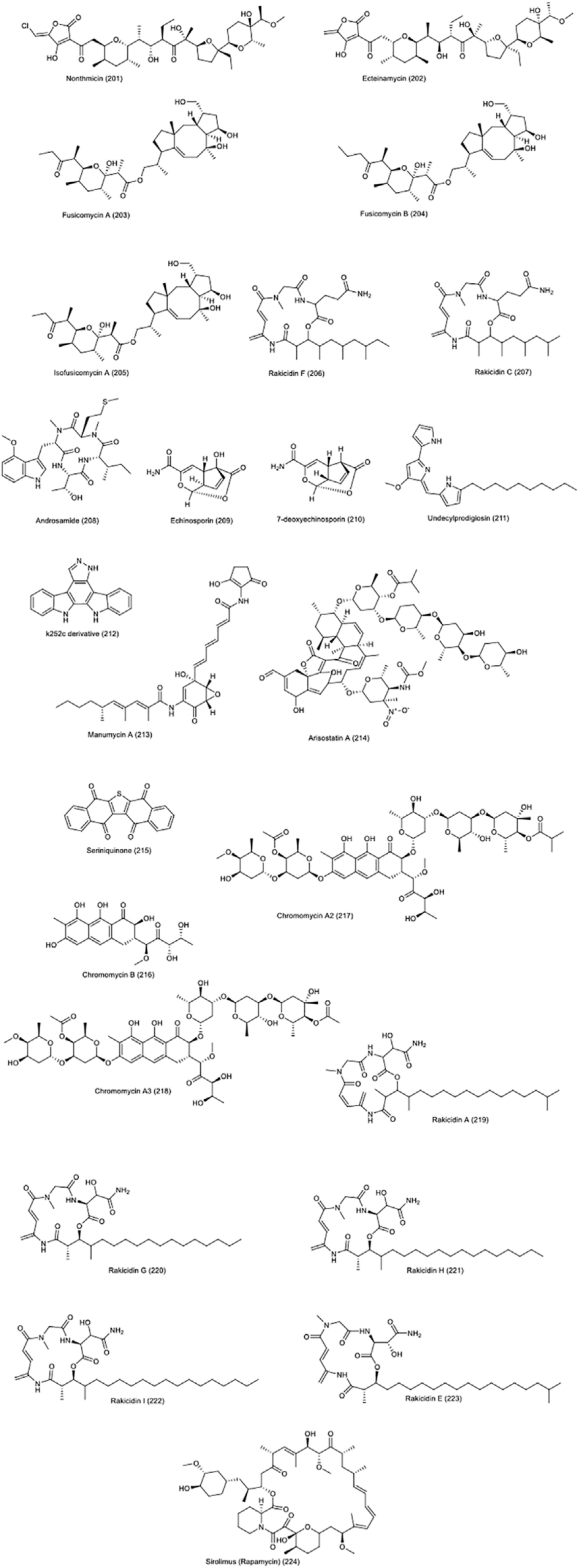
Chemical structure of compounds presented in the Section 2 “Potential biological mechanisms and function of NPs against CRC.”

Two tetrocarcin analogues called arisostatins A **(214)** and B were isolated from *Micromonospora* sp. Strain TP-A0316. Arisostatin A showed a good activity against gram-positive bacteria as well as cytotoxicity and antitumor activity against myeloid leukemia cell line U937 and human colon cancer cell line HCC2998 (IC_50_ = 0.22M), breast, lung and brain (Furumai et al., 2000). A 2003 study examined the antitumor effect of this compound on the head and neck cancer cell line; AMC-HN-4. It has been documented that the antitumor effect and the onset of apoptosis in cells treated with this compound is due to the activation of Caspase-3 and the formation of ROS species (Kim et al., 2003). Chemical structure of compounds 201 to 224 are presented in Figure 10.

### 3.3 Autophagy

A study was performed to evaluate the autophagy and apoptosis properties of the compound seriniquinone **(215)**, produced by *Serinicoccus* sp., an actinobacter from the family *Ornithinimicrobiaceae* on various cell lines, including HCT-15 colon cancer cell line (Trzoss et al., 2014). These studies, generally performed on melanoma cell line and HCT-116 colon cancer, suggested that the compound induces autophagocytosis in the examined cells by targeting dermcidin protein; a protein involved in autophagy and apoptosis processes.

Aureolic acid compounds, called chromomycins B, A2, A3 **(216–218)**, from marine actinomycetes *Streptomyces* sp. WBF16 were showed significant antitumor effects against several cancer cell lines, including HCT-116 and HepG2, SGC7901, A549, and COC1. In 2016, a study found that the compound chromomycin A2 exerts its antitumor effects on cancer cells by inducing autophagy through members of the TP53 family (Lu et al., 2012; Ratovitski, 2016).

### 3.4 Angiogenesis and Hypoxia

Angiogenesis is the process of forming new blood vessels from existing ones that occurs in both physiological and pathological conditions. Various factors are involved in the initiation and development of this process, perhaps the most important of which is VEGF (Nathan and Kannan, 2020). One of the pathological conditions in which angiogenesis is involved is tumor growth and progression. Under hypoxia and oxidative stress, cells begin the process of angiogenesis through factors such as HIF-1α to maintain their survival. The formation of new blood vessels in hypoxic areas of solid tumor cells, such as colon cancer, will lead to better nutrition of the cells and help them to grow and metastasize, and thus tumor progression. It has been shown that the levels of factors involved in angiogenesis and metastasis like VEGF and matrix metalloproteinases (MMPs) increase in the tumor tissue of patients with colon cancer (Auyeung and Ko, 2017).

Many studies are being conducted on the relationship between angiogenesis and cancer in order to identify new factors involved in this process. Factors or compounds that can inhibit angiogenesis or disrupt the process in any way, may be good candidates for the treatment, diagnosis and management of cancer. One of them is TSGA10. It has been shown that the over expression of the compound is associated with decreased HIF-1α transcriptional activity as well as disruption of HIF-1α axis (Mansouri et al., 2016; Amoorahim et al., 2020).

**FIGURE 11:**
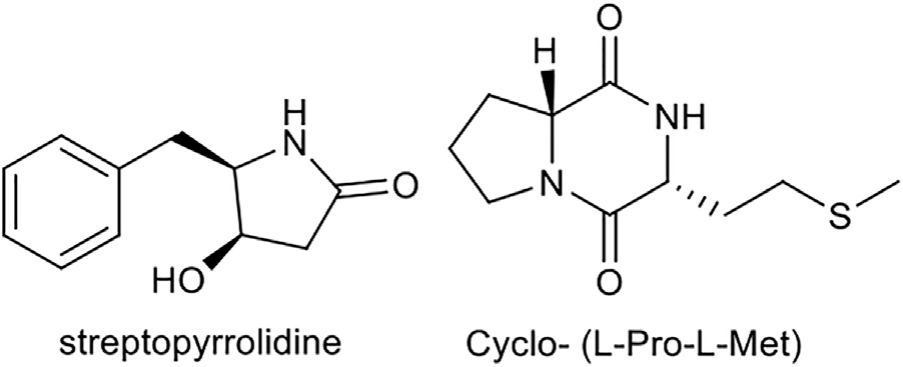
Chemical structure of streptopyrrolidine, and cyclo- (L-Pro-L-Met).

Among the various compounds such as NPs produced by plants and bacteria that affect the process of angiogenesis, NPs of actinomycetic origin, especially marine species, have received increasing attention (Nathan and Kannan, 2020). In a 2008 study, the compound streptopyrrolidine, isolated from the marine derived *Streptomyces* sp. KORDI-3973, after structural and biological examination, has shown an inhibitory effect on the process of angiogenesis (Shin et al., 2008). This compound exhibits a significant effect on HUVECs cells in the presence and absence of VEGF in the study of tube formation assay. The treatment of these cells with streptopyrrolidine, resulted in significant blockade of capillary tube formation, equivalent to the effect of known anti-angiogenesis compound, SU11248. This compound did not show cytotoxicity against the cells used, at of 100 μg/ml concentration. Cyclo- (L-Pro-L-Met) compound, isolated from marine *actinomycete Nocardiopsis* sp. 03N67 was stated to inhibit the angiogenesis, invasion and cell migration of epithelial cells in similar studies using capillary tube formation and migration/invasion assay, at a concentration of 10 µM (Shin et al., 2010). The compound had no cytotoxicity at the reported concentration. Figure 11 illustrates the chemical structure of these compounds.

In 1995, for the first time, the production of rakicidins A and B, from the soil *Micromonospora* sp. strain No. R385-2, was reported. These compounds showed cytotoxicity against M109 lung cancer cell line (McBrien et al., 1995). In subsequent studies in 2006, rakicidin A **(219)** was reported as a compound with selective toxicity against solid tumors in hypoxic conditions. Hypoxia is a hallmark of solid tumors and has been linked to the angiogenesis and related factors such as HIF-1 and VEGF in tumor progression and spread. the various cell lines, including HCT-8 and DLD-1, related to human colon cancer, were examined and this compound showed 17.5 times more toxicity in hypoxia as compared to normal conditions. In further studies, no change in HIF-1 transcriptional level was observed and the mechanism of action of this compound in hypoxic conditions remained unknown (Yamazaki et al., 2007). In 2018, rakicidins G-I **(220–222)** compounds were isolated from the marine derived *Micromonospora chalcea* FIM 02–523 (Chen L. et al., 2018). These compounds along with known compound rakicidin E **(223)**, were tested against pancreatic PANC-1 and colon HCT-8 cancer cell lines in both normal oxygen and hypoxia conditions in which they were 18.2–20.3 times more toxic in hypoxia conditions.

**CHART 2:**
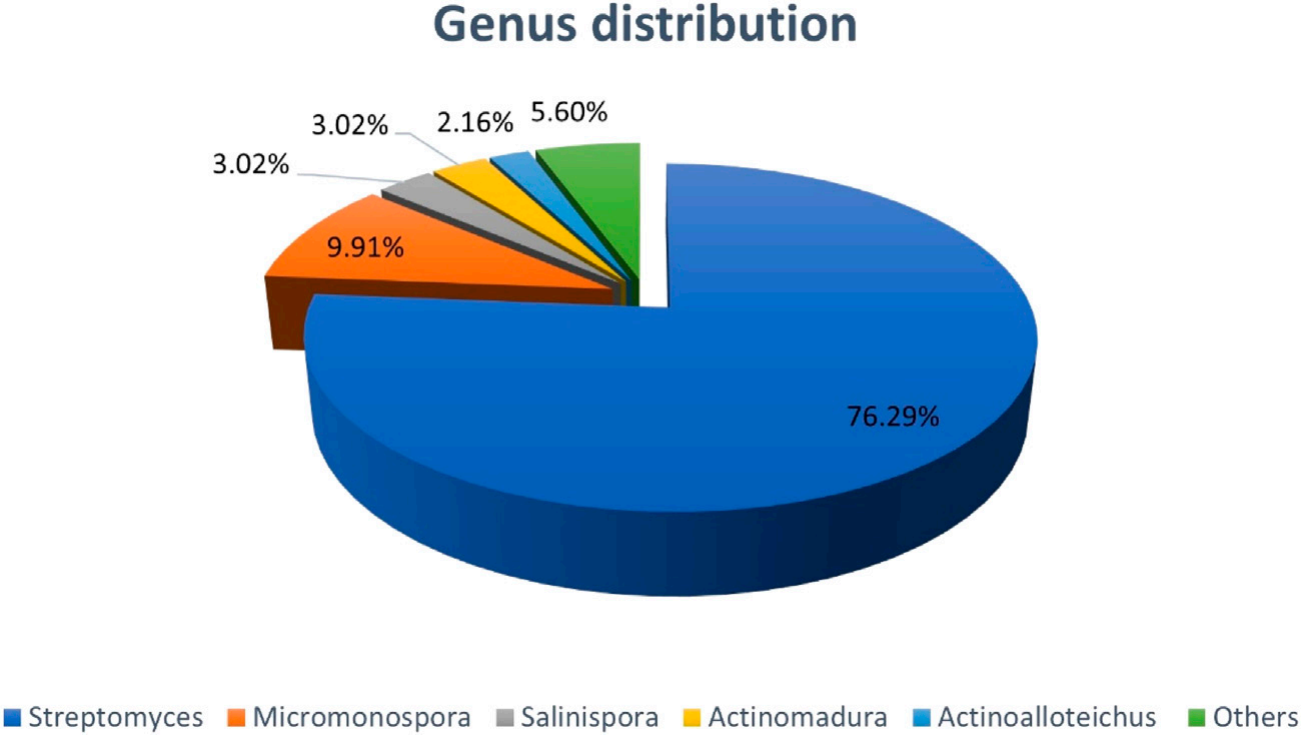
Distribution of NPs with anti-CRC properties produced by Actinobacteria based on Genus. The section entitled “Others”; green aera, includes genera such as *Nocardiosis, Verrucosispora, Amycolatopsis, Pseudonocardia, Microbacterium, Saccharomonospora, Ornithinimicrobiac*.

The distribution of NPs having anti-CRC activities, produced by actinobacteria based on genus is summarized in Chart 2. Chart 2 demonstrates that most of compounds are of *Streptomyces* genera.

## 4 The Structure–Activity Relationship of Natural Products

**CHART 3:**
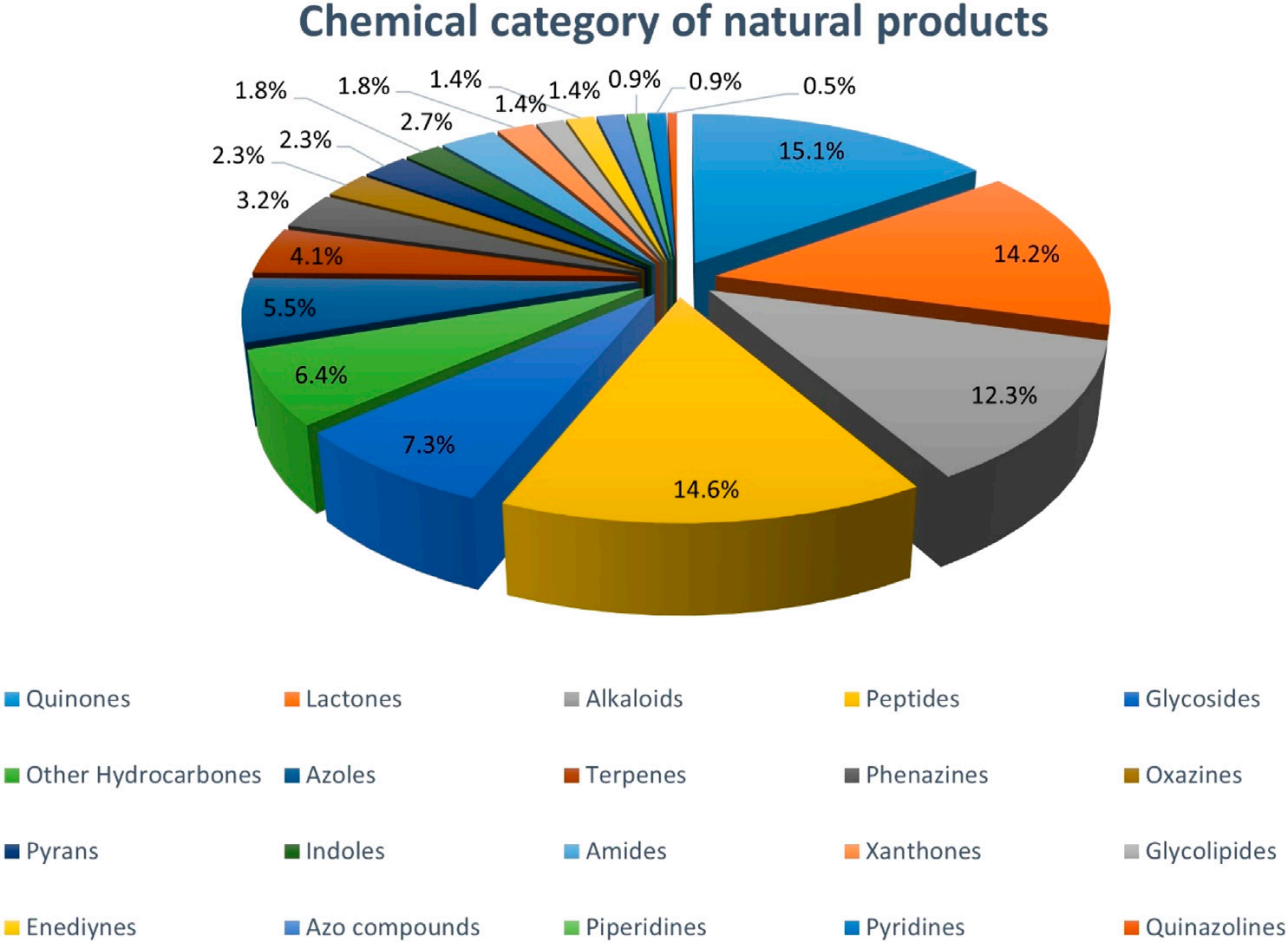
Chemical classification of NPs with anti-CRC properties produced by *Actinobacteria*.

The NPs presented in this paper include a wide range of chemical compounds with unique properties. We have tried to put all of them in 20 different chemical categories. As you can see in Chart 3, more than 54% of the compounds placed into only four major categories, including quinones, lactones, alkaloids, and peptides. Quinones, which are undoubtedly one of the largest groups of antitumor compounds, account for 16% of the NPs in our study. Although the exact relationship between the quinone moiety in these compounds and the cytotoxic effect is not fully understood, it seems that they often exert their biological effects by targeting DNA (Asche, 2005). The second major category belongs to lactones, accounting for 15% of these NPs. Lactones are able to affect cell growth, signaling and differentiation. So, they can exhibit biological effects such as antimicrobial and anticancer properties due to the functional groups attached to them (Konaklieva and Plotkin, 2005).

Here are provided some examples of effective NPs on CRC cell lines with different structural features in order to express the importance of the smallest changes in the chemical structure of compounds in their cytotoxic and anticancer performance.

**FIGURE 12:**
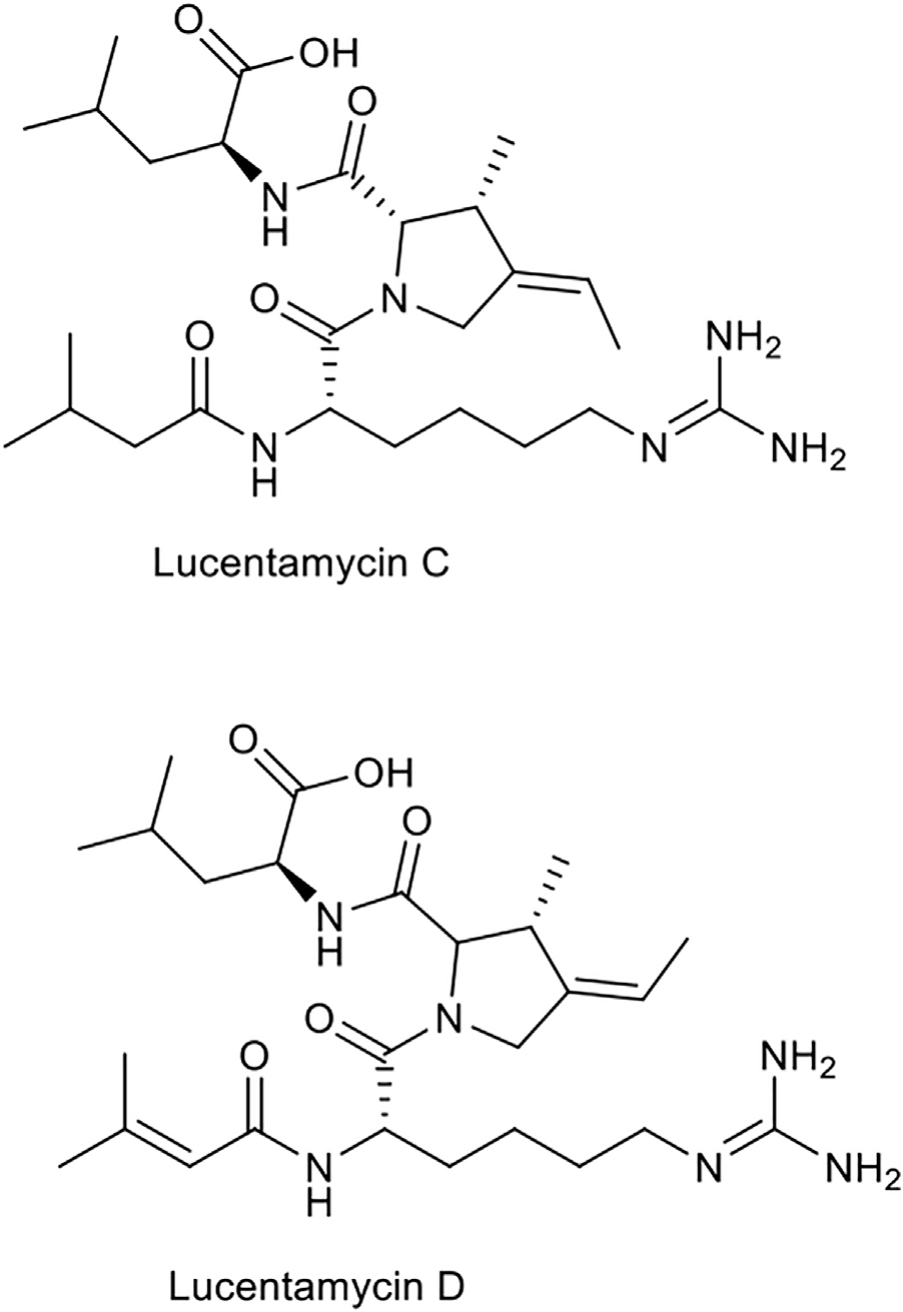
Chemical structure of lucentamycins C and D.

The obvious difference in the biological function of lucentamycins, suggests the presence of aromatic rings in the structure of compounds is pivotal for their cytotoxic activity (Cho et al., 2007). So that, compounds lucentamycins A and B **(23,24)** having aromatic ring (phenyl and indole rings respectively) showed 75 and 13.6 times more cytotoxicity than lucentamycins C and D (which lack of this moieties) against HCT-116, respectively. Figure 12 shows the chemical structure of these compounds.

Another example is the differences between central spiroaminal organization and tetrahydropyran conformation in marineosins A and B **(29,30)** have been reported as reasons for the superiority of type A in causing cytotoxicity on the HCT-116 cell line as compared to type B of the compound (Boonlarppradab et al., 2008).

The presence of sugar bridge and bisindolocarbazole is shown to impact on the activity of compounds. For instances, Structure-activity relationship for staurosporine derivatives **(39,52)**, and strong cytotoxic activity for staurosporine derivative No. 7 **(44)** and No. 14 **(51)**, showed the importance of sugar bridge and bisindolocarbazole in those whose exhibited stronger cytotoxicity against cancer cell lines (Zhou et al., 2019).

The presence of halogens into the structures of natural products or synthetic compounds has often reported to improve their biological activity and physicochemical properties. Actinobacteria, in particular, marine *streptomyces* species generate a diverse range of halogenated compounds with a wide spectrum of biological activities. These compounds possess various chemical structures including polyketides, alkaloids (nitrogen-containing compounds) and terpenoids (Wang et al., 2021). It was reported that halogenated substances from marine actinomycetes have crucial biological activities such as antibacterial and anticancer properties. In the case of chlorizidine **(99)**, the presence of the chlorinated 5H-pyrrolo [2,1-a] isoindol-5-one ring, which had not previously been reported in natural compounds, was a possible factor in the effect of cytotoxicity against HCT-116 cell line (Alvarez-Mico et al., 2013).

Another group of natural compounds that have a special chemical structure are halogenated compounds or halometabolites. Halogenation is a common modification of secondary metabolites and can have a critical role in establishing the bioactivity of a compound. The presence of halogen substituents (F, Cl, Br, I) in their structure can usually add and lead to increase the efficacy and properties of a compound including stability and bioactivity. Having various functions, such as the production of toxins and antibodies and other biochemical properties, are due to the presence of halometabolites in their hosts (Kasanah and Triyanto, 2019). The high concentration of chloride and bromine ions in marine environments, it leads to the discovery of more halogenated compounds in secondary metabolites of marine origin than its terrestrial equivalents (Wang et al., 2021).

Compounds such as marinopyrroles A-F **(86–91)**, ammosamides A **(93)** and B **(94)** and marinocyanins A-F **(100–105)** are types of halometabolites, produced by actinobacteria that have been reported to possess significant cytotoxicity against CRC cell lines (Hughes et al., 2008; Hughes et al., 2009a; Doi et al., 2012; Pan et al., 2012; Asolkar et al., 2017).

In a 2012 study, the presence of a conjugated diene unit and a suitable alkyl chain length were shown to be critical for demonstrating specific activity in rakicidin A **(219)** which is cytotoxic against various cell lines (Oku et al., 2014).

## 5 Cell Lines Used to Study the Anti Colorectal Cancer Property of Compounds

**CHART 4:**
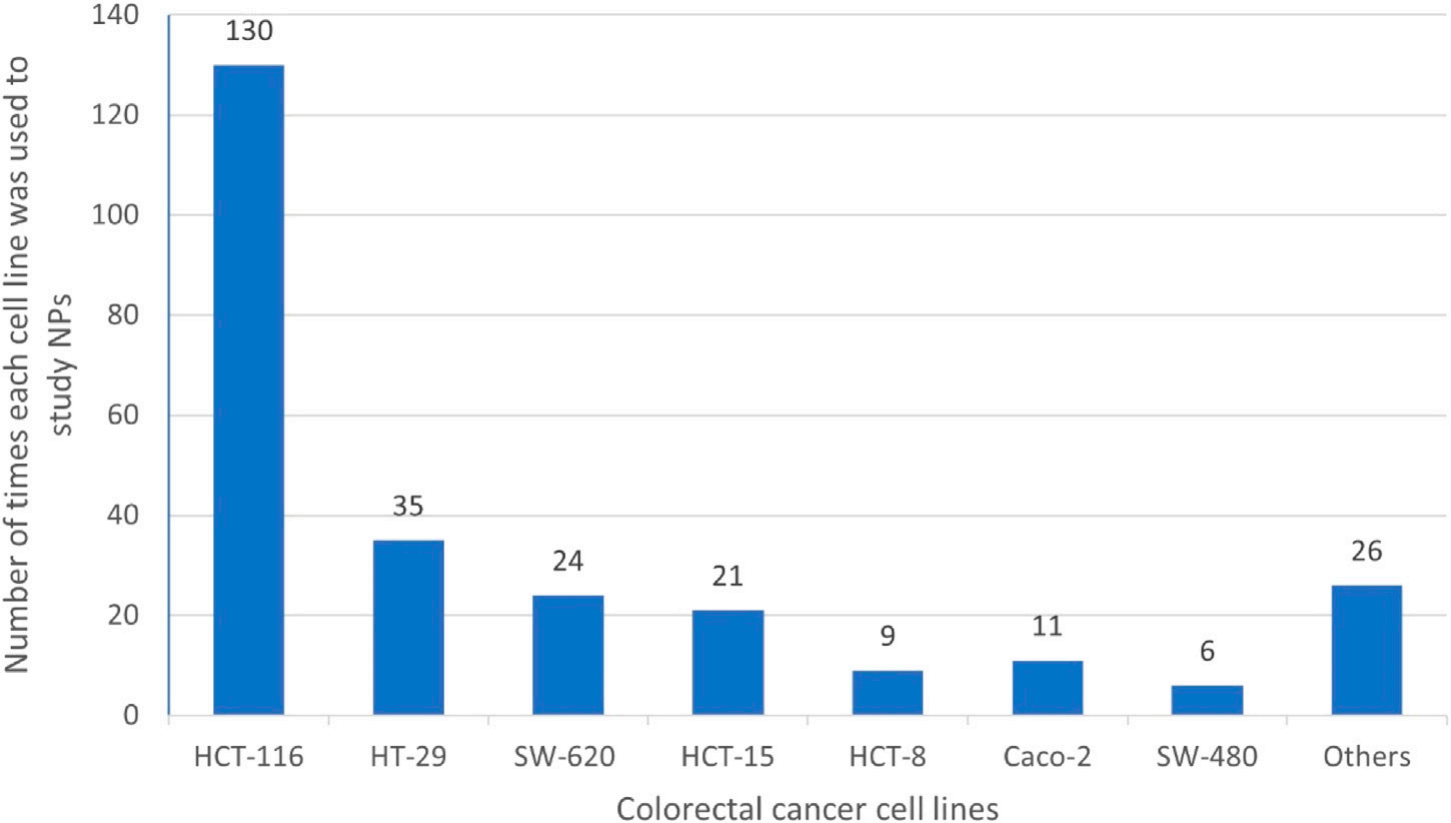
Frequency of CRC cell lines used to evaluate the effectiveness of various natural compounds from actinobacteria.

Our study reveals that a diverse range of cell lines have been employed to assess the anti CRC properties of compounds isolated from actinobacteria which are summarized in Chart 4. While each of them can be classified into one of four categories of CMS based on their molecular, genetic and other characteristics and have their own importance. The most common cell lines, been used to study anti CRC of actinobacterial compounds are, HCT-116, followed by the HT-29 with much lower frequency as compared with the former one. These cell lines possess some of the main features of different types of CRC. The selection of proper cancer cell lines can greatly influence the output of a biological experiment. Useful information can be obtained by selecting the appropriate cell line to perform baseline studies on newly identified compounds. For example, HCT-116 cell line based on its characteristics can be classified as CMS4, which is a mesenchymal CRC with the ability of tissue invasion and angiogenesis, whereas the HT-29 cell line is classified as CMS3, a class that includes epithelial cells with metabolic disorders (Berg et al., 2017). HCT-116 cell line has an MSI status and among its important genes, KRAS and PIK3CA are mutated, while HT-29 has an MSS status and BRAF, PIK3CA and TP53 genes are mutated in this cell line (Ahmed et al., 2013). The presence of such molecular differences in cell lines is representative of clinical differences in various types of CRC and also results in differences in response to treatment with different drugs. the utilization of such cell lines in baseline studies has paved the way for future studies in the clinical phase.

## 6 FDA-Approved Drugs of Actinobacteria Origin Affecting Colorectal Cancer

Mitomycin C is a natural compound isolated from *Streptomyces caespitosus* in 1950. This compound has attracted the attention of scientists by demonstrating selective inhibition of DNA synthesis, mutagenesis, recombination stimulation, chromosome breakdown, DNA crosslinking, etc. as an antibiotic-antitumor. Since then, many studies have been performed to reveal how the cell cycle stop and induces apoptosis by this compound in cancer cells (Tomasz, 1995). Among many studies conducted, it is notable to mention the effect of combination therapy of using mitomycin C and irinotecan in the treatment of CRC, which provides very good results with high tolerance (Ducreux et al., 1998). Mitomycin is currently used as an approved drug in the treatment of various neoplasms, including neoplasms of the breast, bladder, gastrointestinal tract such as colon carcinoma, etc., and even in glaucoma surgery (PubChem, 2004a).

Doxorubicin (DXR), also called adriamycin, is an Anthracyclines compound produced by *Streptomyces peucetius* ATCC 27952, and has reported to have the inhibitory properties on DNA topoisomerase II. This approved drug has anti-cancer effects against various cancers, including thyroid, breast, lung and ovarian cancers, and so on (PubChem, 2004b). Although, in the case of CRC, sometimes the situation is different due to factors such as hereditary resistance to anthracyclines. For example, a study showed that treatment of HT-29 and SW480 cells with aldose reductase (AR) inhibitors such as fidarestat is increased the effect of this drug on cancer cells (Sonowal et al., 2017).

Paclitaxel is a chemotherapy drug that is consumed to treat various solid tumors such as breast, ovary, prostate, lung, etc. In the case of the HT-29-D4 colon cancer cell line, a 2000 study reported that the compound induces apoptosis and activates caspase-8, which is not CD95/CD95-L-dependent (Gonçalves et al., 2000; Trzoss et al., 2014). In 2000, the drug was derived from the endophytic actinomycete *Kitasatospora* sp, isolated from *Taxus baccata* (Caruso et al., 2000; PubChem, 2004c).

Sirolimus (Rapamycin) **(224)** is a compound first isolated from soil *Streptomyces hygroscopicus*. Due to its inhibitory effect on the mTOR pathway, this compound and its derivatives can treat a wide range of diseases such as diabetes, tuberous sclerosis, neurodegenerative and others. Among the therapeutic effects of this compound, anti-CRC properties have also been reported (Li et al., 2014; Mussin et al., 2017).

Everolimus (Afinitor; Novartis) is a derivative of abovementioned compound that has received FDA approval for the treatment of kidney and pancreatic cancers. The use of Everolimus, in combination with compounds such as bevacizumab and mFOLFOX-6 as combination therapy to treat metastatic colorectal cancer, has had significant effects which is in the clinical trial phase (Atkins et al., 2009; Weldon Gilcrease et al., 2019).

## 7 Discussion

CRC is a common, deadly and heterogeneous disease that kills nearly 900,000 people worldwide each year (Sung et al., 2021). The need for targeted, personalized therapies, as well as those with fewer side effects, has led researchers to constantly look for effective drugs for this disease in various sources, of which natural actinobacterial products have been no exception in recent decades. To the best of our knowledge this is the first study on natural compounds having anti CRC properties from actinobacteria. In reviewing the studies of few past decades on natural actinobacterial products, affecting CRC, our findings revealed the structure of 232 NPs with the properties against CRC cell lines, which are produced by over 119 strains of actinobacteria, the majority of them having a chemical structure of quinones, lactones, alkaloids, and peptides (Chart 3). Over 76% of compounds are produced by *streptomyces* strain exclusively. Marine actinobacteria are predominant producers of the anti-colorectal cancer compounds (79.02%), following by terrestrial and endophytic strains counting for 11.16 and 2.68%, respectively. The rest of compounds (7.14%) have been produced by strains living in unconventional environments, synthetic strains (Chart 1). Although more attention has been paid to marine actinobacteria in this review, this significant number of compounds, shows the great importance of marine actinobacteria in the field of bioactive products. Even though, the relatively small number of compounds presented here was from the terrestrial ecosystem does not mean to negate the extraordinary importance of soil actinobacteria.

As expected, most of the NPs reported in this review are produced by *Streptomyces* (76.29%), as they constitute the largest genus of actinobacteria. *Micromonospora* and *Salinispora*, both members of the family *Micromonosporaceae*, and *Actinomadura*, with production of 9.91%, 3.02% and 3.02% of anti-CRC compounds, respectively, appear to be other important genera in this field.

The compounds show enormous biological activities, from autophagy, angiogenesis, invasion, migration, apoptosis, to a range of cytotoxicity, some in terms of values comparable to conventional drug compounds to very strong levels of toxicity (with IC_50_ values in ng scale). Even though the mechanism of action of most compounds was not addressed and determined by abundant of published papers, it is notable that these compounds are able to exert their antitumor properties and toxicity through various pathways. Therefore, the biological process and mechanism of actions of compounds involved in the anti-cancer effects is needed to be investigated further. In addition, further studies in this field can reveal the secret of the protective effects of actinobacteria associated with gut and colorectal cancer more clearly. The knowledge of how gut actinobacteria along with lifestyle and microenvironment factors can interfere with the development of CRC at early stage is pivotal to design biomarkers for CRC.

Despite the advances in compound discovery and structure elucidation, our knowledge on structure activities relationship is still limited. Therefore, mining of structure activity relationship is another field, which requires further exploration. In addition, the molecular docking studies might indicate the role of halogen bonding in complexes and uncover the secret of structure activities relationship.

Another point to note is the use of diverse CRC cell lines in previous studies, while in most of them, the reason for selecting each cell line was not specified. It is also clear that the frequency of use of these cell lines is very unequal. Based on the present evidence, the anti-CRC effect of natural actinobacterial compounds, less attention has been paid to the two groups CMS1 and CMS2 and their associated cell lines. In other words, although most of these researches are preliminary studies of the cytotoxic and antitumor effects of natural compounds, it is worth to use cell lines belonging to two or more different CMSs simultaneously, in order to consider the heterogeneity of this cancer and to comprehensively investigate the antitumor effects of a compound.

## Glossary

5-FU: 5-fluorouracil
Akt or PKB: Protein kinase B
CIMP: CpG island methylator phenotype
CIN: Chromosomal instability
CLL: Chronic lymphocytic leukemia
CMSs: Consensus molecular subtypes
CRC: Colorectal cancer
EGFR: Epidermal growth factor receptor
EMT: Epithelial-to-mesenchymal transition
FDA: Food and drug administration
HIF-1α: Hypoxia-inducible factor 1-alpha
HR-MS: High resolution mass spectrometry
IBD: Inflammatory bowel disease
KRAS gene: Kirsten rat sarcoma virus gene
MAPK: Mitogen-activated protein kinase
MMPs: Matrix metalloproteinases
MRSA: Methicillin-resistant *Staphylococcus aureus*
MSI: Microsatellite instability
mTOR: Mammalian target of rapamycin
NF-κB: nuclear factor ‘kappa-light-chain-enhancer’ of activated B-cells
NPs: Natural products
PARP: Poly (ADP-ribose) polymerase
PI3K: Phosphoinositide 3-kinase
RAS: Rat sarcoma virus
ROS: Reactive oxygen species
SML: Sea surface microlayer
SRB assay: Sulforhodamine B assay
STAT3: Signal transducer and activator of transcription 3
TNF: Tumor necrosis factor
TNM: Tumor node metastasis
TRAIL: Tumor necrosis factor-related apoptosis-inducing ligand
TSGA10: Testis-specific Gene Antigen 10
VEGF: Vascular endothelial growth factor
XIAP: X-linked inhibitor of apoptosis
ZEB1 or 2: Zinc finger E-box binding homeobox 1or 2

## References

[B1] Abedinlou H. Bahrami Y. Mohammadi S. Kakaei E. (2022). Rare Actinobacteria and Their Potential Biotechnological Applications. Sci. J. Kurdistan Univ. Med. Sci. 26 (7), 108–131.

[B2] Ahmed D. Eide P. W. Eilertsen I. A. Danielsen S. A. Eknæs M. Hektoen M. (2013). Epigenetic and Genetic Features of 24 Colon Cancer Cell Lines. Oncogenesis 2 (9), e71. 10.1038/oncsis.2013.35 24042735 PMC3816225

[B3] Ahn K. S. Sethi G. Chao T. H. Neuteboom S. T. Chaturvedi M. M. Palladino M. A. (2007). Salinosporamide A (NPI-0052) Potentiates Apoptosis, Suppresses Osteoclastogenesis, and Inhibits Invasion through Down-Modulation of NF-kappaB Regulated Gene Products. Blood 110 (7), 2286–2295. 10.1182/blood-2007-04-084996 17609425 PMC1988928

[B4] Almeida L. C. Bauermeister A. Rezende-Teixeira P. Santos E. A. D. Moraes L. A. B. Machado-Neto J. A. (2019). Pradimicin-IRD Exhibits Antineoplastic Effects by Inducing DNA Damage in Colon Cancer Cells. Biochem. Pharmacol. 168, 38–47. 10.1016/j.bcp.2019.06.016 31228463

[B5] Alvarez-Mico X. Jensen P. R. Fenical W. Hughes C. C. (2013). Chlorizidine, a Cytotoxic 5H-Pyrrolo[2,1-A]isoindol-5-One-Containing Alkaloid from a Marine *Streptomyces* Sp. Org. Lett. 15 (5), 988–991. 10.1021/ol303374e 23405849 PMC3702164

[B6] Amoorahim M. Valipour E. Hoseinkhani Z. Mahnam A. Rezazadeh D. Ansari M. (2020). TSGA10 Overexpression Inhibits Angiogenesis of HUVECs: A HIF-2α Biased Perspective. Microvasc. Res. 128, 103952. 10.1016/j.mvr.2019.103952 31704243

[B7] Arcamone F. Cassinelli G. Fantini G. Grein A. Orezzi P. Pol C. (1969). Adriamycin, 14-hydroxydaunomycin, a New Antitumor Antibiotic from *S. Peucetius Var. Caesius* . Biotechnol. Bioeng. 11 (6), 1101–1110. 10.1002/bit.260110607 5365804

[B8] Arnold M. Sierra M. S. Laversanne M. Soerjomataram I. Jemal A. Bray F. (2017). Global Patterns and Trends in Colorectal Cancer Incidence and Mortality. Gut 66 (4), 683–691. 10.1136/gutjnl-2015-310912 26818619

[B9] Asche C. (2005). Antitumour Quinones. Mini Rev. Med. Chem. 5 (5), 449–467. 10.2174/1389557053765556 15892687

[B10] Asolkar R. N. Freel K. C. Jensen P. R. Fenical W. Kondratyuk T. P. Park E. J. (2009). Arenamides A-C, Cytotoxic NFkappaB Inhibitors from the Marine Actinomycete *Salinispora Arenicola* . J. Nat. Prod. 72 (3), 396–402. 10.1021/np800617a 19117399 PMC2837138

[B11] Asolkar R. N. Jensen P. R. Kauffman C. A. Fenical W. (2006). Daryamides A-C, Weakly Cytotoxic Polyketides from a Marine-Derived Actinomycete of the Genus *Streptomyces* Strain CNQ-085. J. Nat. Prod. 69 (12), 1756–1759. 10.1021/np0603828 17190455

[B12] Asolkar R. N. Kirkland T. N. Jensen P. R. Fenical W. (2010). Arenimycin, an Antibiotic Effective against Rifampin- and Methicillin-Resistant *Staphylococcus aureus* from the Marine Actinomycete *Salinispora Arenicola* . J. Antibiot. (Tokyo) 63 (1), 37–39. 10.1038/ja.2009.114 19927167 PMC2825372

[B13] Asolkar R. N. Singh A. Jensen P. R. Aalbersberg W. Carté B. K. Feussner K. D. (2017). Marinocyanins, Cytotoxic Bromo-Phenazinone Meroterpenoids from a Marine Bacterium from the *Streptomycete* Clade MAR4. Tetrahedron 73 (16), 2234–2241. 10.1016/j.tet.2017.03.003 28814819 PMC5555602

[B14] Atkins M. B. Yasothan U. Kirkpatrick P. (2009). Everolimus. Nat. Rev. Drug Discov. 8 (7), 535–536. 10.1038/nrd2924 19568281

[B15] Auyeung K. K. Ko J. K. (2017). Angiogenesis and Oxidative Stress in Metastatic Tumor Progression: Pathogenesis and Novel Therapeutic Approach of Colon Cancer. Curr. Pharm. Des. 23 (27), 3952–3961. 10.2174/1381612823666170228124105 28245762

[B16] Bach D. H. Kim S. H. Hong J. Y. Park H. J. Oh D. C. Lee S. K. (2015). Salternamide A Suppresses Hypoxia-Induced Accumulation of HIF-1α and Induces Apoptosis in Human Colorectal Cancer Cells. Mar. Drugs 13 (11), 6962–6976. 10.3390/md13116962 26610526 PMC4663561

[B17] Bahrami Y. Delbari Y. Buzhani K. R. Kakaei E. Mohassel Y. Bouk S. (2022). “Endophytic Actinobacteria in Biosynthesis of Bioactive Metabolites and Their Application in Improving Crop Yield and Sustainable Agriculture,” in Natural Products from Actinomycetes: Diversity, Ecology and Drug Discovery. Editors Rai, R.V. Bai J.A. (Singapore: Springer Singapore), 119–150. 10.1007/978-981-16-6132-7_5

[B18] Bailly C. (2019). Irinotecan: 25 Years of Cancer Treatment. Pharmacol. Res. 148, 104398. 10.1016/j.phrs.2019.104398 31415916

[B19] Barka E. A. Vatsa P. Sanchez L. Gaveau-Vaillant N. Jacquard C. Meier-Kolthoff J. P. (2016). Taxonomy, Physiology, and Natural Products of Actinobacteria. Microbiol. Mol. Biol. Rev. 80 (1), 1–43. 10.1128/mmbr.00019-15 26609051 PMC4711186

[B20] Baudino T. A. (2015). Targeted Cancer Therapy: The Next Generation of Cancer Treatment. Curr. Drug Discov. Technol. 12 (1), 3–20. 10.2174/1570163812666150602144310 26033233

[B21] Bauermeister A. Calil F. A. das C L Pinto F. Medeiros T. C. T. Almeida L. C. Silva L. J. (2019). Pradimicin-IRD from *Amycolatopsis* Sp. IRD-009 and its Antimicrobial and Cytotoxic Activities. Nat. Prod. Res. 33 (12), 1713–1720. 10.1080/14786419.2018.1434639 29451013

[B22] Berg K. C. G. Eide P. W. Eilertsen I. A. Johannessen B. Bruun J. Danielsen S. A. (2017). Multi-omics of 34 Colorectal Cancer Cell Lines - a Resource for Biomedical Studies. Mol. Cancer 16 (1), 116. 10.1186/s12943-017-0691-y 28683746 PMC5498998

[B23] Bernardi D. I. das Chagas F. O. Monteiro A. F. dos Santos G. F. de Souza Berlinck R. G. (2019). “Secondary Metabolites of Endophytic Actinomycetes: Isolation, Synthesis, Biosynthesis, and Biological Activities,” in Progress in the Chemistry of Organic Natural Products 108. Editors Kinghorn A.D. Falk H. Gibbons S. Kobayashi J.i. Asakawa Y. Liu J.-K. (Cham: Springer International Publishing) . 10.1007/978-3-030-01099-7_3 30924015

[B24] Boonlarppradab C. Kauffman C. A. Jensen P. R. Fenical W. (2008). Marineosins A and B, Cytotoxic Spiroaminals from a Marine-Derived Actinomycete. Org. Lett. 10 (24), 5505–5508. 10.1021/ol8020644 19007176 PMC2654761

[B25] Bull A. T. Stach J. E. Ward A. C. Goodfellow M. (2005). Marine Actinobacteria: Perspectives, Challenges, Future Directions. Ant. Van Leeuwenhoek 87 (1), 65–79. 10.1007/s10482-004-6562-8 15726293

[B26] Byun W. S. Kim S. Shin Y. H. Kim W. K. Oh D. C. Lee S. K. (2020). Antitumor Activity of Ohmyungsamycin A through the Regulation of the Skp2-P27 Axis and MCM4 in Human Colorectal Cancer Cells. J. Nat. Prod. 83 (1), 118–126. 10.1021/acs.jnatprod.9b00918 31894983

[B27] Cañedo L. M. Puentes J. L. F. Baz J. P. Huang X.-H. Rinehart K. L. (2000). IB-96212, a Novel Cytotoxic Macrolide Produced by a Marine *Micromonospora*. II. Physico-Chemical Properties and Structure Determination. J. Antibiot. 53 (5), 479–483. 10.7164/antibiotics.53.479 10908111

[B28] Caruso M. Colombo A. Fedeli L. Pavesi A. Quaroni S. Saracchi M. (2000). Isolation of Endophytic Fungi and Actinomycetes Taxane Producers. Ann. Microbiol. 50 (1), 3–14.

[B29] Chen C. Ye Y. Wang R. Zhang Y. Wu C. Debnath S. C. (2018a). *Streptomyces Nigra Sp. Nov.* Is a Novel Actinobacterium Isolated from Mangrove Soil and Exerts a Potent Antitumor Activity *In Vitro* . Front. Microbiol. 9, 1587. 10.3389/fmicb.2018.01587 30072967 PMC6058180

[B30] Chen L. Zhao W. Jiang H.-L. Zhou J. Chen X.-M. Lian Y.-Y. (2018b). Rakicidins G - I, Cyclic Depsipeptides from Marine *Micromonospora Chalcea* FIM 02-523. Tetrahedron 74 (30), 4151–4154. 10.1016/j.tet.2018.06.039

[B31] Cheng C. Othman E. M. Stopper H. Edrada-Ebel R. Hentschel U. Abdelmohsen U. R. (2017). Isolation of Petrocidin A, a New Cytotoxic Cyclic Dipeptide from the Marine Sponge-Derived Bacterium Streptomyces Sp. SBT348. Mar. Drugs 15 (12), 383. 10.3390/md15120383 PMC5742843 29211005

[B32] Cheng Y. B. Jensen P. R. Fenical W. (2013). Cytotoxic and Antimicrobial Napyradiomycins from Two Marine-Derived, MAR 4 *Streptomyces* Strains. Eur. J. Org. Chem. 2013 (18), 3751–3757. 10.1002/ejoc.201300349 PMC3873167 24376369

[B33] Cho J. Y. Williams P. G. Kwon H. C. Jensen P. R. Fenical W. (2007). Lucentamycins A-D, Cytotoxic Peptides from the Marine-Derived Actinomycete *Nocardiopsis Lucentensis* . J. Nat. Prod. 70 (8), 1321–1328. 10.1021/np070101b 17630797

[B34] Choi B. K. Lee H. S. Kang J. S. Shin H. J. (2019). Dokdolipids A-C, Hydroxylated Rhamnolipids from the Marine-Derived Actinomycete *Actinoalloteichus Hymeniacidonis* . Mar. Drugs 17 (4), 237. 10.3390/md17040237 PMC6521253 31010028

[B35] Conti R. Chagas F. O. Caraballo-Rodriguez A. M. Melo W. G. do Nascimento A. M. Cavalcanti B. C. (2016). Endophytic Actinobacteria from the Brazilian Medicinal Plant *Lychnophora Ericoides Mart*. And the Biological Potential of Their Secondary Metabolites. Chem. Biodivers. 13 (6), 727–736. 10.1002/cbdv.201500225 27128202

[B36] Cui C. B. Liu H. B. Gu J. Y. Gu Q. Q. Cai B. Zhang D. Y. (2007). Echinosporins as New Cell Cycle Inhibitors and Apoptosis Inducers from Marine-Derived *Streptomyces Albogriseolus* . Fitoterapia 78 (3), 238–240. 10.1016/j.fitote.2006.11.017 17376609

[B37] Cusack J. C. Jr. Liu R. Xia L. Chao T. H. Pien C. Niu W. (2006). NPI-0052 Enhances Tumoricidal Response to Conventional Cancer Therapy in a Colon Cancer Model. Clin. Cancer Res. 12 (22), 6758–6764. 10.1158/1078-0432.Ccr-06-1151 17121896

[B38] de Gramont A. Figer A. Seymour M. Homerin M. Hmissi A. Cassidy J. (2000). Leucovorin and Fluorouracil with or without Oxaliplatin as First-Line Treatment in Advanced Colorectal Cancer. J. Clin. Oncol. 18 (16), 2938–2947. 10.1200/jco.2000.18.16.2938 10944126

[B39] Delbari Y. Mohassel Y. Bahrami Y. Kakaie E. Mostafaie A. (2020). A Review on Isolation and Identification of Endophytic Actinobacteria, Their Chemical Structure, Bioactive Compounds, and Potential Medical-Pharmaceutical Applications. J J. Mazandaran Univ. Med. Sci. 30 (186), 195–217.

[B40] Deng L. J. Qi M. Li N. Lei Y. H. Zhang D. M. Chen J. X. (2020). Natural Products and Their Derivatives: Promising Modulators of Tumor Immunotherapy. J. Leukoc. Biol. 108 (2), 493–508. 10.1002/JLB.3MR0320-444R 32678943 PMC7496826

[B41] Di Paolo A. Danesi R. Nardini D. Bocci G. Innocenti F. Fogli S. (2000). Manumycin Inhibits Ras Signal Transduction Pathway and Induces Apoptosis in COLO320-DM Human Colon Tumour Cells. Br. J. Cancer 82 (4), 905–912. 10.1054/bjoc.1999.1018 10732765 PMC2374379

[B42] Dinesh R. Srinivasan V. T E S. Anandaraj M. Srambikkal H. (2017). Endophytic Actinobacteria: Diversity, Secondary Metabolism and Mechanisms to Unsilence Biosynthetic Gene Clusters. Crit. Rev. Microbiol. 43 (5), 546–566. 10.1080/1040841x.2016.1270895 28358596

[B43] Ding L. Ndejouong Ble. S. Maier A. Fiebig H. H. Hertweck C. (2012). Elaiomycins D-F, Antimicrobial and Cytotoxic Azoxides from *Streptomyces* Sp. Strain HKI0708. J. Nat. Prod. 75 (10), 1729–1734. 10.1021/np300329m 23013356

[B44] Ding L. Pfoh R. Rühl S. Qin S. Laatsch H. (2009). T-muurolol Sesquiterpenes from the Marine *Streptomyces* Sp. M491 and Revision of the Configuration of Previously Reported Amorphanes. J. Nat. Prod. 72 (1), 99–101. 10.1021/np8006843 19072130

[B45] Ding N. Jiang Y. Han L. Chen X. Ma J. Qu X. (2016). Bafilomycins and Odoriferous Sesquiterpenoids from *Streptomyces Albolongus* Isolated from elephas Maximus Feces. J. Nat. Prod. 79 (4), 799–805. 10.1021/acs.jnatprod.5b00827 26933756

[B46] Doi K. Li R. Sung S. S. Wu H. Liu Y. Manieri W. (2012). Discovery of Marinopyrrole A (Maritoclax) as a Selective Mcl-1 Antagonist that Overcomes ABT-737 Resistance by Binding to and Targeting Mcl-1 for Proteasomal Degradation. J. Biol. Chem. 287 (13), 10224–10235. 10.1074/jbc.M111.334532 22311987 PMC3323047

[B47] Dong M. Cao P. Ma Y. T. Luo J. Yan Y. Li R. T. (2019). A New Actinomycin Z Analogue with an Additional Oxygen Bridge between Chromophore and β-depsipentapeptide from Streptomyces Sp. KIB-H714. Nat. Prod. Res. 33 (2), 219–225. 10.1080/14786419.2018.1443097 29495881

[B48] Ducreux M. Gil-Delgado M. André T. Ychou M. de Gramond A. Khayat D. (1998). Irinotecan in Combination for Colon Cancer. Bull. Cancer Spec No, 43–46. 9932084

[B49] Duncan K. R. Crüsemann M. Lechner A. Sarkar A. Li J. Ziemert N. (2015). Molecular Networking and Pattern-Based Genome Mining Improves Discovery of Biosynthetic Gene Clusters and Their Products from *Salinispora* Species. Chem. Biol. 22 (4), 460–471. 10.1016/j.chembiol.2015.03.010 25865308 PMC4409930

[B50] El-Hawary S. S. Sayed A. M. Mohammed R. Khanfar M. A. Rateb M. E. Mohammed T. A. (2018). New Pim-1 Kinase Inhibitor from the Co-culture of Two Sponge-Associated Actinomycetes. Front. Chem. 6, 538. 10.3389/fchem.2018.00538 30525020 PMC6262321

[B51] Elmallah M. I. Y. Cogo S. Constantinescu A. A. Elifio-Esposito S. Abdelfattah M. S. Micheau O. (2020). Marine Actinomycetes-Derived Secondary Metabolites Overcome TRAIL-Resistance via the Intrinsic Pathway through Downregulation of Survivin and XIAP. Cells 9 (8), 1760. 10.3390/cells9081760 PMC7464567 32708048

[B52] Erba E. Bergamaschi D. Ronzoni S. Faretta M. Taverna S. Bonfanti M. (1999). Mode of Action of Thiocoraline, a Natural Marine Compound with Anti-tumour Activity. Br. J. Cancer 80 (7), 971–980. 10.1038/sj.bjc.6690451 10362104 PMC2363046

[B53] Esvan Y. J. Giraud F. Pereira E. Suchaud V. Nauton L. Théry V. (2016). Synthesis and Biological Activity of Pyrazole Analogues of the Staurosporine Aglycon K252c. Bioorg Med. Chem. 24 (14), 3116–3124. 10.1016/j.bmc.2016.05.032 27255178

[B54] Farnaes L. Coufal N. G. Kauffman C. A. Rheingold A. L. DiPasquale A. G. Jensen P. R. (2014). Napyradiomycin Derivatives, Produced by a Marine-Derived Actinomycete, Illustrate Cytotoxicity by Induction of Apoptosis. J. Nat. Prod. 77 (1), 15–21. 10.1021/np400466j 24328269 PMC3913167

[B55] Fearon E. R. Vogelstein B. (1990). A Genetic Model for Colorectal Tumorigenesis. Cell. 61 (5), 759–767. 10.1016/0092-8674(90)90186-i 2188735

[B56] Fei P. Chuan-Xi W. Yang X. Hong-Lei J. Lu-Jie C. Uribe P. (2013). A New 20-membered Macrolide Produced by a Marine-Derived *Micromonospora* Strain. Nat. Prod. Res. 27 (15), 1366–1371. 10.1080/14786419.2012.740038 23157320

[B57] Feling R. H. Buchanan G. O. Mincer T. J. Kauffman C. A. Jensen P. R. Fenical W. (2003). Salinosporamide A: a Highly Cytotoxic Proteasome Inhibitor from a Novel Microbial Source, a Marine Bacterium of the New Genus *Salinospora* . Angew. Chem. Int. Ed. Engl. 42 (3), 355–357. 10.1002/anie.200390115 12548698

[B58] Fernández-chimeno R. I. Cañedo L. Espliego F. Grávalos D. Calle F. D. L. Fernández-puentes J. L. (2000). IB-96212, a Novel Cytotoxic Macrolide Produced by a Marine *Micromonospora*. I. Taxonomy, Fermentation, Isolation and Biological Activities. J. Antibiot. 53 (5), 474–478. 10.7164/antibiotics.53.474 10908110

[B59] Fu P. Zhu Y. Mei X. Wang Y. Jia H. Zhang C. (2014). Acyclic Congeners from *Actinoalloteichus Cyanogriseus* Provide Insights into Cyclic Bipyridine Glycoside Formation. Org. Lett. 16 (16), 4264–4267. 10.1021/ol5019757 25090585

[B60] Furumai T. Igarashi Y. Higuchi H. Saito N. Oki T. (2002). Kosinostatin, a Quinocycline Antibiotic with Antitumor Activity from *Micromonospora* Sp. TP-A0468. J. Antibiot. (Tokyo) 55 (2), 128–133. 10.7164/antibiotics.55.128 12002993

[B61] Furumai T. Takagi K. Igarashi Y. Saito N. Oki T. (2000). Arisostatins A and B, New Members of Tetrocarcin Class of Antibiotics from *Micromonospora* Sp. TP-A0316. I. Taxonomy, Fermentation, Isolation and Biological Properties. J. Antibiot. (Tokyo) 53 (3), 227–232. 10.7164/antibiotics.53.227 10819292

[B62] Gao R. Kong C. Huang L. Li H. Qu X. Liu Z. (2017). Mucosa-associated Microbiota Signature in Colorectal Cancer. Eur. J. Clin. Microbiol. Infect. Dis. 36 (11), 2073–2083. 10.1007/s10096-017-3026-4 28600626

[B63] Gao X. Lu Y. Xing Y. Ma Y. Lu J. Bao W. (2012). A Novel Anticancer and Antifungus Phenazine Derivative from a Marine Actinomycete BM-17. Microbiol. Res. 167 (10), 616–622. 10.1016/j.micres.2012.02.008 22494896

[B64] Gonçalves A. Braguer D. Carles G. André N. Prevôt C. Briand C. (2000). Caspase-8 Activation Independent of CD95/CD95-L Interaction during Paclitaxel-Induced Apoptosis in Human Colon Cancer Cells (HT29-D4). Biochem. Pharmacol. 60 (11), 1579–1584. 10.1016/s0006-2952(00)00481-0 11077039

[B65] Gui C. Yuan J. Mo X. Huang H. Zhang S. Gu Y. C. (2018). Cytotoxic Anthracycline Metabolites from a Recombinant *Streptomyces* . J. Nat. Prod. 81 (5), 1278–1289. 10.1021/acs.jnatprod.8b00212 29767975

[B66] Guillaumot M. A. Cerles O. Bertrand H. C. Benoit E. Nicco C. Chouzenoux S. (2019). Oxaliplatin-induced Neuropathy: the Preventive Effect of a New Super-oxide Dismutase Modulator. Oncotarget 10 (60), 6418–6431. 10.18632/oncotarget.27248 31741707 PMC6849645

[B67] Guinney J. Dienstmann R. Wang X. de Reyniès A. Schlicker A. Soneson C. (2015). The Consensus Molecular Subtypes of Colorectal Cancer. Nat. Med. 21 (11), 1350–1356. 10.1038/nm.3967 26457759 PMC4636487

[B68] Gulder T. A. Moore B. S. (2010). Salinosporamide Natural Products: Potent 20 S Proteasome Inhibitors as Promising Cancer Chemotherapeutics. Angew. Chem. Int. Ed. Engl. 49 (49), 9346–9367. 10.1002/anie.201000728 20927786 PMC3103133

[B69] Hale V. L. Chen J. Johnson S. Harrington S. C. Yab T. C. Smyrk T. C. (2017). Shifts in the Fecal Microbiota Associated with Adenomatous Polyps. Cancer Epidemiol. Biomarkers Prev. 26 (1), 85–94. 10.1158/1055-9965.Epi-16-0337 27672054 PMC5225053

[B70] Hara M. Akasaka K. Akinaga S. Okabe M. Nakano H. Gomez R. (1993). Identification of Ras Farnesyltransferase Inhibitors by Microbial Screening. Proc. Natl. Acad. Sci. U. S. A. 90 (6), 2281–2285. 10.1073/pnas.90.6.2281 8460134 PMC46070

[B71] Hardt I. H. Jensen P. R. Fenical W. (2000). Neomarinone, and New Cytotoxic Marinone Derivatives, Produced by a Marine Filamentous Bacterium (Actinomycetales). Tetrahedron Lett. 41 (13), 2073–2076. 10.1016/s0040-4039(00)00117-9

[B72] Hayakawa Y. Shirasaki S. Kawasaki T. Matsuo Y. Adachi K. Shizuri Y. (2007a). Structures of New Cytotoxic Antibiotics, Piericidins C7 and C8. J. Antibiot. (Tokyo) 60 (3), 201–203. 10.1038/ja.2007.23 17446693

[B73] Hayakawa Y. Shirasaki S. Shiba S. Kawasaki T. Matsuo Y. Adachi K. (2007b). Piericidins C7 and C8, New Cytotoxic Antibiotics Produced by a Marine *Streptomyces* Sp. J. Antibiot. (Tokyo) 60 (3), 196–200. 10.1038/ja.2007.22 17446692

[B74] He H. Ding W. D. Bernan V. S. Richardson A. D. Ireland C. M. Greenstein M. (2001). Lomaiviticins A and B, Potent Antitumor Antibiotics from *Micromonospora Lomaivitiensis* . J. Am. Chem. Soc. 123 (22), 5362–5363. 10.1021/ja010129o 11457405

[B75] Hernández L. M. Blanco J. A. Baz J. P. Puentes J. L. Millán F. R. Vázquez F. E. (2000). 4'-N-methyl-5'-hydroxystaurosporine and 5'-hydroxystaurosporine, New Indolocarbazole Alkaloids from a Marine *Micromonospora* Sp. Strain. J. Antibiot. (Tokyo) 53 (9), 895–902. 10.7164/antibiotics.53.895 11099222

[B76] Hu X. Sun W. Li S. Li L. Yu L. Liu H. (2020). Cervinomycins C1-4 with Cytotoxic and Antibacterial Activity from Streptomyces Sp. CPCC 204980. J. Antibiot. (Tokyo) 73 (12), 812–817. 10.1038/s41429-020-0342-1 32616897

[B77] Huang X. M. Yang Z. J. Xie Q. Zhang Z. K. Zhang H. Ma J. Y. (2019). Natural Products for Treating Colorectal Cancer: A Mechanistic Review. Biomed. Pharmacother. 117, 109142. 10.1016/j.biopha.2019.109142 31238258

[B78] Hughes C. C. MacMillan J. B. Gaudêncio S. P. Fenical W. La Clair J. J. (2009a). Ammosamides A and B Target Myosin. Angew. Chem. Int. Ed. Engl. 48 (4), 728–732. 10.1002/anie.200804107 19097126 PMC2820877

[B79] Hughes C. C. MacMillan J. B. Gaudêncio S. P. Jensen P. R. Fenical W. (2009b). The Ammosamides: Structures of Cell Cycle Modulators from a Marine-Derived *Streptomyces* Species. Angew. Chem. Int. Ed. Engl. 48 (4), 725–727. 10.1002/anie.200804890 19090514 PMC2819817

[B80] Hughes C. C. Prieto-Davo A. Jensen P. R. Fenical W. (2008). The Marinopyrroles, Antibiotics of an Unprecedented Structure Class from a Marine *Streptomyces* Sp. Org. Lett. 10 (4), 629–631. 10.1021/ol702952n 18205372 PMC2820876

[B81] Hwang J. H. Kim J. Y. Cha M. R. Ryoo I. J. Choo S. J. Cho S. M. (2008). Etoposide-resistant HT-29 Human Colon Carcinoma Cells during Glucose Deprivation Are Sensitive to Piericidin A, a GRP78 Down-Regulator. J. Cell. Physiol. 215 (1), 243–250. 10.1002/jcp.21308 17941090

[B82] Igarashi Y. Matsuoka N. In Y. In T. Tashiro E. Saiki I. (2017). Nonthmicin, a Polyether Polyketide Bearing a Halogen-Modified Tetronate with Neuroprotective and Antiinvasive Activity from *Actinomadura* Sp. Org. Lett. 19 (6), 1406–1409. 10.1021/acs.orglett.7b00318 28256141

[B83] Igarashi Y. Trujillo M. E. Martínez-Molina E. Yanase S. Miyanaga S. Obata T. (2007). Antitumor Anthraquinones from an Endophytic Actinomycete *Micromonospora Lupini* Sp. Nov. Bioorg Med. Chem. Lett. 17 (13), 3702–3705. 10.1016/j.bmcl.2007.04.039 17475486

[B84] Itoh T. Kinoshita M. Aoki S. Kobayashi M. (2003). Komodoquinone A, a Novel Neuritogenic Anthracycline, from Marine *Streptomyces* Sp. KS3. J. Nat. Prod. 66 (10), 1373–1377. 10.1021/np030212k 14575440

[B85] Jensen P. R. Williams P. G. Oh D. C. Zeigler L. Fenical W. (2007). Species-specific Secondary Metabolite Production in Marine Actinomycetes of the Genus *Salinispora* . Appl. Environ. Microbiol. 73 (4), 1146–1152. 10.1128/aem.01891-06 17158611 PMC1828645

[B86] Jeong S. Y. Shin H. J. Kim T. S. Lee H. S. Park S. K. Kim H. M. (2006). Streptokordin, a New Cytotoxic Compound of the Methylpyridine Class from a Marine-Derived *Streptomyces* Sp. KORDI-3238. J. Antibiot. (Tokyo) 59 (4), 234–240. 10.1038/ja.2006.33 16830891

[B87] Jiang Y. J. Li J. Q. Zhang H. J. Ding W. J. Ma Z. J. (2018a). Cyclizidine-type Alkaloids from *Streptomyces* Sp. HNA39. J. Nat. Prod. 81 (2), 394–399. 10.1021/acs.jnatprod.7b01055 29389122

[B88] Jiang Y. J. Zhang D. S. Zhang H. J. Li J. Q. Ding W. J. Xu C. D. (2018b). Medermycin-type Naphthoquinones from the Marine-Derived *Streptomyces* Sp. XMA39. J. Nat. Prod. 81 (9), 2120–2124. 10.1021/acs.jnatprod.8b00544 30209946

[B89] Jiang Z. K. Guo L. Chen C. Liu S. W. Zhang L. Dai S. J. (2015). Xiakemycin A, a Novel Pyranonaphthoquinone Antibiotic, Produced by the *Streptomyces* Sp. CC8-201 from the Soil of a Karst Cave. J. Antibiot. (Tokyo) 68 (12), 771–774. 10.1038/ja.2015.70 26104142

[B90] Kalaitzis J. A. Hamano Y. Nilsen G. Moore B. S. (2003). Biosynthesis and Structural Revision of Neomarinone. Org. Lett. 5 (23), 4449–4452. 10.1021/ol035748b 14602022

[B91] Kasanah N. Triyanto T. (2019). Bioactivities of Halometabolites from Marine Actinobacteria. Biomolecules 9 (6), 225. 10.3390/biom9060225 PMC6627970 31212626

[B92] Kaweewan I. Komaki H. Hemmi H. Hoshino K. Hosaka T. Isokawa G. (2019). Isolation and Structure Determination of a New Cytotoxic Peptide, Curacozole, from *STREPTOMYCES CURACOI* Based on Genome Mining. J. Antibiot. (Tokyo) 72 (1), 1–7. 10.1038/s41429-018-0105-4 30310179

[B93] Khalifa S. A. M. Elias N. Farag M. A. Chen L. Saeed A. Hegazy M. F. (2019). Marine Natural Products: A Source of Novel Anticancer Drugs. Mar. Drugs 17 (9), 491. 10.3390/md17090491 PMC6780632 31443597

[B94] Khan N. Yılmaz S. Aksoy S. Uzel A. Tosun Ç. Kirmizibayrak P. B. (2019). Polyethers Isolated from the Marine Actinobacterium Streptomyces Cacaoi Inhibit Autophagy and Induce Apoptosis in Cancer Cells. Chem. Biol. Interact. 307, 167–178. 10.1016/j.cbi.2019.04.035 31059704

[B95] Kim S. H. Shin Y. Lee S. H. Oh K. B. Lee S. K. Shin J. (2015). Salternamides A-D from a Halophilic *Streptomyces* Sp. Actinobacterium. J. Nat. Prod. 78 (4), 836–843. 10.1021/acs.jnatprod.5b00002 25700232

[B96] Kim Y. H. Shin H. C. Song D. W. Lee S. H. Furumai T. Park J. W. (2003). Arisostatins A Induces Apoptosis through the Activation of Caspase-3 and Reactive Oxygen Species Generation in AMC-HN-4 Cells. Biochem. Biophys. Res. Commun. 309 (2), 449–456. 10.1016/j.bbrc.2003.07.009 12951070

[B97] Kitani S. Ueguchi T. Igarashi Y. Leetanasaksakul K. Thamchaipenet A. Nihira T. (2017). Rakicidin F, a New Antibacterial Cyclic Depsipeptide from a Marine Sponge-Derived *Streptomyces* Sp. J. Antibiot. 71, 139–141. 10.1038/ja.2017.92 28765588

[B98] Konaklieva M. I. Plotkin B. J. (2005). Lactones: Generic Inhibitors of Enzymes? Mini Rev. Med. Chem. 5 (1), 73–95. 10.2174/1389557053402828 15638793

[B99] Kværner A. S. Birkeland E. Bucher-Johannessen C. Vinberg E. Nordby J. I. Kangas H. (2021). The CRCbiome Study: a Large Prospective Cohort Study Examining the Role of Lifestyle and the Gut Microbiome in Colorectal Cancer Screening Participants. BMC Cancer 21 (1), 930. 10.1186/s12885-021-08640-8 34407780 PMC8371800

[B100] Kwon H. C. Espindola A. P. Park J. S. Prieto-Davó A. Rose M. Jensen P. R. (2010). Nitropyrrolins A-E, Cytotoxic Farnesyl-α-Nitropyrroles from a Marine-Derived Bacterium within the Actinomycete Family Streptomycetaceae. J. Nat. Prod. 73 (12), 2047–2052. 10.1021/np1006229 21090803 PMC3077121

[B101] Kwon H. C. Kauffman C. A. Jensen P. R. Fenical W. (2006). Marinomycins A-D, Antitumor-Antibiotics of a New Structure Class from a Marine Actinomycete of the Recently Discovered Genus "marinispora". J. Am. Chem. Soc. 128 (5), 1622–1632. 10.1021/ja0558948 16448135

[B102] Kwon Y. Kim S. H. Shin Y. Bae M. Kim B. Y. Lee S. K. (2014). A New Benzofuran Glycoside and Indole Alkaloids from a Sponge-Associated Rare Actinomycete, *Amycolatopsis* Sp. Mar. Drugs 12 (4), 2326–2340. 10.3390/md12042326 24759001 PMC4012469

[B103] Lee J. Gamage C. D. B. Kim G. J. Hillman P. F. Lee C. Lee E. Y. (2020). Androsamide, a Cyclic Tetrapeptide from a Marine *Nocardiopsis* sp., Suppresses Motility of Colorectal Cancer Cells. J. Nat. Prod. 83 (10), 3166–3172. 10.1021/acs.jnatprod.0c00815 32985880

[B104] Lewin G. R. Carlos C. Chevrette M. G. Horn H. A. McDonald B. R. Stankey R. J. (2016). Evolution and Ecology of Actinobacteria and Their Bioenergy Applications. Annu. Rev. Microbiol. 70, 235–254. 10.1146/annurev-micro-102215-095748 27607553 PMC5703056

[B105] Li J. Kim S. G. Blenis J. (2014). Rapamycin: One Drug, Many Effects. Cell. Metab. 19 (3), 373–379. 10.1016/j.cmet.2014.01.001 24508508 PMC3972801

[B106] Li W. Yang X. Yang Y. Zhao L. Xu L. Ding Z. (2015). A New Anthracycline from Endophytic *Streptomyces* Sp. YIM66403. J. Antibiot. (Tokyo) 68 (3), 216–219. 10.1038/ja.2014.128 25248729

[B107] Li X. B. Tang J. S. Gao H. Ding R. Li J. Hong K. (2011). A New Staurosporine Analog from Actinomycetes *Streptomyces* Sp. (172614). J. Asian Nat. Prod. Res. 13 (8), 765–769. 10.1080/10286020.2011.586342 21751847

[B108] Liu C. X. Liu S. H. Zhao J. W. Zhang J. Wang X. J. Li J. S. (2017). A New Spectinabilin Derivative with Cytotoxic Activity from Ant-Derived *Streptomyces* Sp. 1H-GS5. J. Asian Nat. Prod. Res. 19 (9), 924–929. 10.1080/10286020.2016.1254200 27838921

[B109] Liu D. Lin H. Proksch P. Tang X. Shao Z. Lin W. (2015). Microbacterins A and B, New Peptaibols from the Deep Sea Actinomycete *Microbacterium Sediminis* Sp. Nov. YLB-01(T). Org. Lett. 17 (5), 1220–1223. 10.1021/acs.orglett.5b00172 25675340

[B110] Liu R. Cui C. B. Duan L. Gu Q. Q. Zhu W. M. (2005). Potent *In Vitro* Anticancer Activity of Metacycloprodigiosin and Undecylprodigiosin from a Sponge-Derived Actinomycete *Saccharopolyspora* Sp. Nov. Arch. Pharm. Res. 28 (12), 1341–1344. 10.1007/bf02977899 16392666

[B111] Liu R. Zhu T. Li D. Gu J. Xia W. Fang Y. (2007). Two Indolocarbazole Alkaloids with Apoptosis Activity from a Marine-Derived Actinomycete Z(2)039-2. Arch. Pharm. Res. 30 (3), 270–274. 10.1007/bf02977605 17424930

[B112] Lu C. Zhao Y. Jia W. Q. Zhang H. Qi H. Xiang W. S. (2017). A New Anthracycline-type Metabolite from *Streptomyces* Sp. NEAU-L3. J. Antibiot. (Tokyo) 70 (10), 1026–1028. 10.1038/ja.2017.95 28811620

[B113] Lu J. Ma Y. Liang J. Xing Y. Xi T. Lu Y. (2012). Aureolic Acids from a Marine-Derived *Streptomyces* Sp. WBF16. Microbiol. Res. 167 (10), 590–595. 10.1016/j.micres.2012.06.001 22789867

[B114] Lv Q. Fan Y. Tao G. Fu P. Zhai J. Ye B. (2019). Sekgranaticin, a SEK34b-Granaticin Hybrid Polyketide from *Streptomyces* Sp. 166. J. Org. Chem. 84 (14), 9087–9092. 10.1021/acs.joc.9b01022 31273973

[B115] Lynch H. T. de la Chapelle A. (2003). Hereditary Colorectal Cancer. N. Engl. J. Med. 348 (10), 919–932. 10.1056/NEJMra012242 12621137

[B116] Ma Y. Zhang Y. Jiang H. Xiang S. Zhao Y. Xiao M. (2021). Metagenome Analysis of Intestinal Bacteria in Healthy People, Patients with Inflammatory Bowel Disease and Colorectal Cancer. Front. Cell.. Infect. Microbiol. 11, 599734. 10.3389/fcimb.2021.599734 33738265 PMC7962608

[B117] Macherla V. R. Liu J. Bellows C. Teisan S. Nicholson B. Lam K. S. (2005). Glaciapyrroles A, B, and C, Pyrrolosesquiterpenes from a *Streptomyces* Sp. Isolated from an Alaskan Marine Sediment. J. Nat. Prod. 68 (5), 780–783. 10.1021/np049597c 15921430

[B118] Maldonado L. A. Stach J. E. Pathom-aree W. Ward A. C. Bull A. T. Goodfellow M. (2005). Diversity of Cultivable Actinobacteria in Geographically Widespread Marine Sediments. Antonie Leeuwenhoek 87, 11–18. 10.1007/s10482-004-6525-0 15726286

[B119] Malet-cascón L. Romero F. Espliego-vázquez F. Grávalos D. Fernández-puentes J. L. (2003). IB-00208, a New Cytotoxic Polycyclic Xanthone Produced by a Marine-Derived *Actinomadura*. I. Isolation of the Strain, Taxonomy and Biological Activites. J. Antibiot. 56 (3), 219–225. 10.7164/antibiotics.56.219 12760677

[B120] Maloney K. N. Macmillan J. B. Kauffman C. A. Jensen P. R. Dipasquale A. G. Rheingold A. L. (2009). Lodopyridone, a Structurally Unprecedented Alkaloid from a Marine Actinomycete. Org. Lett. 11 (23), 5422–5424. 10.1021/ol901997k 19883103 PMC2820114

[B121] Mansouri K. Mostafie A. Rezazadeh D. Shahlaei M. Modarressi M. H. (2016). New Function of TSGA10 Gene in Angiogenesis and Tumor Metastasis: a Response to a Challengeable Paradox. Hum. Mol. Genet. 25 (2), 233–244. 10.1093/hmg/ddv461 26573430

[B122] Mármol I. Sánchez-de-Diego C. Pradilla Dieste A. Cerrada E. Rodriguez Yoldi M. (2017). Colorectal Carcinoma: A General Overview and Future Perspectives in Colorectal Cancer. Ijms 18 (1), 197. 10.3390/ijms18010197 PMC5297828 28106826

[B123] Martin G. D. Tan L. T. Jensen P. R. Dimayuga R. E. Fairchild C. R. Raventos-Suarez C. (2007). Marmycins A and B, Cytotoxic Pentacyclic C-Glycosides from a Marine Sediment-Derived Actinomycete Related to the Genus *Streptomyces* . J. Nat. Prod. 70 (9), 1406–1409. 10.1021/np060621r 17844998

[B124] Maskey R. P. Helmke E. Kayser O. Fiebig H. H. Maier A. Busche A. (2004a). Anti-cancer and Antibacterial Trioxacarcins with High Anti-malaria Activity from a Marine *Streptomycete* and Their Absolute Stereochemistry. J. Antibiot. (Tokyo) 57 (12), 771–779. 10.7164/antibiotics.57.771 15745111

[B125] Maskey R. P. Li F. Qin S. Fiebig H. H. Laatsch H. (2003). Chandrananimycins A Approximately C: Production of Novel Anticancer Antibiotics from a Marine *Actinomadura* Sp. Isolate M048 by Variation of Medium Composition and Growth Conditions. J. Antibiot. (Tokyo) 56 (7), 622–629. 10.7164/antibiotics.56.622 14513905

[B126] Maskey R. P. Sevvana M. Usón I. Helmke E. Laatsch H. (2004b). Gutingimycin: A Highly Complex Metabolite from a Marine *Streptomycete* . Angew. Chem. Int. Ed. Engl. 43 (10), 1281–1283. 10.1002/anie.200352312 14991799

[B127] McBrien K. D. Berry R. L. Lowe S. E. Neddermann K. M. Bursuker I. Huang S. (1995). Rakicidins, New Cytotoxic Lipopeptides from *Micromonospora* Sp. Fermentation, Isolation and Characterization. J. Antibiot. (Tokyo) 48 (12), 1446–1452. 10.7164/antibiotics.48.1446 8557602

[B128] Miller E. D. Kauffman C. A. Jensen P. R. Fenical W. (2007). Piperazimycins: Cytotoxic Hexadepsipeptides from a Marine-Derived Bacterium of the Genus *Streptomyces* . J. Org. Chem. 72 (2), 323–330. 10.1021/jo061064g 17221946

[B129] Mitchell S. S. Nicholson B. Teisan S. Lam K. S. Potts B. C. (2004). Aureoverticillactam, a Novel 22-atom Macrocyclic Lactam from the Marine Actinomycete *Streptomyces Aureoverticillatus* . J. Nat. Prod. 67 (8), 1400–1402. 10.1021/np049970g 15332863

[B130] Moon K. Ahn C. H. Shin Y. Won T. H. Ko K. Lee S. K. (2014). New Benzoxazine Secondary Metabolites from an Arctic Actinomycete. Mar. Drugs 12 (5), 2526–2538. 10.3390/md12052526 24796308 PMC4052304

[B131] Mori G. Rampelli S. Orena B. S. Rengucci C. De Maio G. Barbieri G. (2018). Shifts of Faecal Microbiota during Sporadic Colorectal Carcinogenesis. Sci. Rep. 8 (1), 10329. 10.1038/s41598-018-28671-9 29985435 PMC6037773

[B132] Mussin N. Oh S. C. Lee K. W. Park M. Y. Seo S. Yi N. J. (2017). Sirolimus and Metformin Synergistically Inhibits Colon Cancer *In Vitro* and *In Vivo* . J. Korean Med. Sci. 32 (9), 1385–1395. 10.3346/jkms.2017.32.9.1385 28776332 PMC5546956

[B133] Nam S. J. Kauffman C. A. Paul L. A. Jensen P. R. Fenical W. (2013). Actinoranone, a Cytotoxic Meroterpenoid of Unprecedented Structure from a Marine Adapted *Streptomyces* Sp. Org. Lett. 15 (21), 5400–5403. 10.1021/ol402080s 24152065 PMC4112586

[B134] Nathan J. Kannan R. R. (2020). Antiangiogenic Molecules from Marine Actinomycetes and the Importance of Using Zebrafish Model in Cancer Research. Heliyon 6 (12), e05662. 10.1016/j.heliyon.2020.e05662 33319107 PMC7725737

[B135] Nguyen H. T. Pokhrel A. R. Nguyen C. T. Pham V. T. T. Dhakal D. Lim H. N. (2020). *Streptomyces Sp.* VN1, a Producer of Diverse Metabolites Including Non-natural Furan-type Anticancer Compound. Sci. Rep. 10 (1), 1756. 10.1038/s41598-020-58623-1 32019976 PMC7000394

[B136] Nikodinovic-Runic J. Mojic M. Kang Y. Maksimovic-Ivanic D. Mijatovic S. Vasiljevic B. (2014). Undecylprodigiosin Conjugated Monodisperse Gold Nanoparticles Efficiently Cause Apoptosis in Colon Cancer Cells *In Vitro* . J. Mater Chem. B 2 (21), 3271–3281. 10.1039/c4tb00300d 32261589

[B137] Oh D. C. Williams P. G. Kauffman C. A. Jensen P. R. Fenical W. (2006). Cyanosporasides A and B, Chloro- and Cyano-Cyclopenta[a]indene Glycosides from the Marine Actinomycete "Salinispora Pacifica". Org. Lett. 8 (6), 1021–1024. 10.1021/ol052686b 16524258

[B138] Oku N. Matoba S. Yamazaki Y. M. Shimasaki R. Miyanaga S. Igarashi Y. (2014). Complete Stereochemistry and Preliminary Structure-Activity Relationship of Rakicidin A, a Hypoxia-Selective Cytotoxin from *Micromonospora* Sp. J. Nat. Prod. 77 (11), 2561–2565. 10.1021/np500276c 25375258

[B139] Omura S. Iwai Y. Hirano A. Nakagawa A. Awaya J. Tsuchya H. (1977). A New Alkaloid AM-2282 of *Streptomyces* Origin. Taxonomy, Fermentation, Isolation and Preliminary Characterization. J. Antibiot. (Tokyo) 30 (4), 275–282. 10.7164/antibiotics.30.275 863788

[B140] Onaka H. (2017). Novel Antibiotic Screening Methods to Awaken Silent or Cryptic Secondary Metabolic Pathways in Actinomycetes. J. Antibiot. (Tokyo) 70 (8), 865–870. 10.1038/ja.2017.51 28442735

[B141] Pan E. Jamison M. Yousufuddin M. MacMillan J. B. (2012). Ammosamide D, an Oxidatively Ring Opened Ammosamide Analog from a Marine-Derived *Streptomyces Variabilis* . Org. Lett. 14 (9), 2390–2393. 10.1021/ol300806e 22515470 PMC3348922

[B142] Pérez M. Crespo C. Schleissner C. Rodríguez P. Zúñiga P. Reyes F. (2009). Tartrolon D, a Cytotoxic Macrodiolide from the Marine-Derived Actinomycete *Streptomyces* Sp. MDG-04-17-069. J. Nat. Prod. 72 (12), 2192–2194. 10.1021/np9006603 19968258

[B143] Pérez M. Schleissner C. Fernández R. Rodríguez P. Reyes F. Zuñiga P. (2016). PM100117 and PM100118, New Antitumor Macrolides Produced by a Marine *Streptomyces Caniferus* GUA-06-05-006A. J. Antibiot. 69 (5), 388–394. 10.1038/ja.2015.121 26648119

[B144] Peters U. Bien S. Zubair N. (2015). Genetic Architecture of Colorectal Cancer. Gut 64 (10), 1623–1636. 10.1136/gutjnl-2013-306705 26187503 PMC4567512

[B145] Piawah S. Venook A. P. (2019). Targeted Therapy for Colorectal Cancer Metastases: A Review of Current Methods of Molecularly Targeted Therapy and the Use of Tumor Biomarkers in the Treatment of Metastatic Colorectal Cancer. Cancer 125 (23), 4139–4147. 10.1002/cncr.32163 31433498

[B146] PubChem (2004b). PubChem Compound Summary for CID 31703, Doxorubicin. Bethesda (MD): National Library of Medicine US. National Center for Biotechnology Information. [Online]. Available: https://pubchem.ncbi.nlm.nih.gov/compound/Doxorubicin (Accessed June 15, 2021).

[B147] PubChem (2004c). PubChem Compound Summary for CID 36314, Paclitaxel. Bethesda (MD): National Library of Medicine (US). National Center for Biotechnology Information. [Online]. Available: https://pubchem.ncbi.nlm.nih.gov/compound/Paclitaxel (Accessed June 16, 2021).

[B148] PubChem (2004a). PubChem Compound Summary for CID 5746, Mitomycin. Bethesda (MD): National Library of Medicine (US). National Center for Biotechnology Information. [Online]. Available: https://pubchem.ncbi.nlm.nih.gov/compound/Mitomycin (Accessed June 9, 2021).

[B149] Ratovitski E. A. (2016). Tumor Protein (TP)-p53 Members as Regulators of Autophagy in Tumor Cells upon Marine Drug Exposure. Mar. Drugs 14 (8), 154. 10.3390/md14080154 PMC4999915 27537898

[B150] Rejhová A. Opattová A. Čumová A. Slíva D. Vodička P. (2018). Natural Compounds and Combination Therapy in Colorectal Cancer Treatment. Eur. J. Med. Chem. 144, 582–594. 10.1016/j.ejmech.2017.12.039 29289883

[B151] Rodríguez J. C. Puentes J. L. F. Baz J. P. Cañedo L. M. (2003). IB-00208, a New Cytotoxic Polycyclic Xanthone Produced by a Marine-Derived *Actinomadura*. II. Isolation, Physico-Chemical Properties and Structure Determination. J. Antibiot. 56 (3), 318–321. 10.7164/antibiotics.56.318 12760690

[B152] Romero F. Espliego F. Pérez Baz J. García de Quesada T. Grávalos D. de la Calle F. (1997). Thiocoraline, a New Depsipeptide with Antitumor Activity Produced by a Marine *Micromonospora*. I. Taxonomy, Fermentation, Isolation, and Biological Activities. J. Antibiot. (Tokyo) 50 (9), 734–737. 10.7164/antibiotics.50.734 9360617

[B153] Sánchez López J. M. Martínez Insua M. Pérez Baz J. Fernández Puentes J. L. Cañedo Hernández L. M. (2003). New Cytotoxic Indolic Metabolites from a Marine *Streptomyces* . J. Nat. Prod. 66 (6), 863–864. 10.1021/np0204444 12828477

[B154] Sattler I. Thiericke R. Zeeck A. (1998). The Manumycin-Group Metabolites. Nat. Prod. Rep. 15 (3), 221–240. 10.1039/a815221y 9652122

[B155] Schneemann I. Kajahn I. Ohlendorf B. Zinecker H. Erhard A. Nagel K. (2010). Mayamycin, a Cytotoxic Polyketide from a Streptomyces Strain Isolated from the Marine Sponge Halichondria Panicea. J. Nat. Prod. 73 (7), 1309–1312. 10.1021/np100135b 20545334

[B156] Shaaban K. A. Shaaban M. Rahman H. Grün-Wollny I. Kämpfer P. Kelter G. (2019). Karamomycins A-C: 2-Naphthalen-2-Yl-Thiazoles from *Nonomuraea Endophytica* . J. Nat. Prod. 82 (4), 870–877. 10.1021/acs.jnatprod.8b00928 30907593

[B157] Shang N. N. Zhang Z. Huang J. P. Wang L. Luo J. Yang J. (2018). Glycosylated Piericidins from an Endophytic *Streptomyces* with Cytotoxicity and Antimicrobial Activity. J. Antibiot. (Tokyo) 71 (7), 672–676. 10.1038/s41429-018-0051-1 29651143

[B158] Shin H. J. Kim T. S. Lee H. S. Park J. Y. Choi I. K. Kwon H. J. (2008). Streptopyrrolidine, an Angiogenesis Inhibitor from a Marine-Derived *Streptomyces* Sp. KORDI-3973. Phytochemistry 69 (12), 2363–2366. 10.1016/j.phytochem.2008.05.020 18649901

[B159] Shin H. J. Lee H. S. Lee J. S. Shin J. Lee M. A. Lee H. S. (2014). Violapyrones H and I, New Cytotoxic Compounds Isolated from *Streptomyces* Sp. Associated with the Marine Starfish *Acanthaster planci* . Mar. Drugs 12 (6), 3283–3291. 10.3390/md12063283 24886866 PMC4071576

[B160] Shin H. J. Mondol M. A. M. Yu T. K. Lee H.-S. Lee Y.-J. Jung H. J. (2010). An Angiogenesis Inhibitor Isolated from a Marine-Derived Actinomycete, *Nocardiopsis* Sp. 03N67. Phytochem. Lett. 3 (4), 194–197. 10.1016/j.phytol.2010.07.005

[B161] Siddharth S. Vittal R. R. (2019). Isolation, Characterization, and Structural Elucidation of 4-methoxyacetanilide from Marine Actinobacteria *Streptomyces* Sp. SCA29 and Evaluation of its Enzyme Inhibitory, Antibacterial, and Cytotoxic Potential. Arch. Microbiol. 201 (6), 737–746. 10.1007/s00203-019-01634-y 30820617

[B162] Sobolevskaia M. P. Kuznetsova T. A. (2010). Biologically Active Metabolites of the Marine Actinobacteria. Bioorg Khim 36 (5), 607–621. 10.1134/s1068162010050031 21063447

[B163] Son S. Ko S. K. Jang M. Lee J. K. Kwon M. C. Kang D. H. (2017). Polyketides and Anthranilic Acid Possessing 6-Deoxy-α-L-Talopyranose from a Streptomyces Species. J. Nat. Prod. 80 (5), 1378–1386. 10.1021/acs.jnatprod.6b01059 28406643

[B164] Song Y. Yang J. Yu J. Li J. Yuan J. Wong N. K. (2020). Chlorinated Bis-Indole Alkaloids from Deep-Sea Derived *Streptomyces* Sp. SCSIO 11791 with Antibacterial and Cytotoxic Activities. J. Antibiot. (Tokyo) 73 (8), 542–547. 10.1038/s41429-020-0307-4 32332871

[B165] Sonowal H. Pal P. B. Wen J. J. Awasthi S. Ramana K. V. Srivastava S. K. (2017). Aldose Reductase Inhibitor Increases Doxorubicin-Sensitivity of Colon Cancer Cells and Decreases Cardiotoxicity. Sci. Rep. 7 (1), 3182. 10.1038/s41598-017-03284-w 28600556 PMC5466629

[B166] Soria-Mercado I. E. Prieto-Davo A. Jensen P. R. Fenical W. (2005). Antibiotic Terpenoid Chloro-Dihydroquinones from a New Marine Actinomycete. J. Nat. Prod. 68 (6), 904–910. 10.1021/np058011z 15974616

[B167] Sousa Tda S. Jimenez P. C. Ferreira E. G. Silveira E. R. Braz-Filho R. Pessoa O. D. (2012). Anthracyclinones from *Micromonospora* Sp. J. Nat. Prod. 75 (3), 489–493. 10.1021/np200795p 22250891

[B168] Stoffel E. M. Kastrinos F. (2014). Familial Colorectal Cancer, beyond Lynch Syndrome. Clin. Gastroenterol. Hepatol. 12 (7), 1059–1068. 10.1016/j.cgh.2013.08.015 23962553 PMC3926911

[B169] Suela Silva M. Naves Sales A. Teixeira Magalhães-Guedes K. Ribeiro Dias D. Schwan R. F. (2013). Brazilian Cerrado Soil Actinobacteria Ecology. Biomed. Res. Int. 2013, 503805. 10.1155/2013/503805 23555089 PMC3595109

[B170] Sung H. Ferlay J. Siegel R. L. Laversanne M. Soerjomataram I. Jemal A. (2021). Global Cancer Statistics 2020: GLOBOCAN Estimates of Incidence and Mortality Worldwide for 36 Cancers in 185 Countries. CA A Cancer J. Clin. 71, 209–249. 10.3322/caac.21660 33538338

[B171] Thomford N. E. Senthebane D. A. Rowe A. Munro D. Seele P. Maroyi A. (2018). Natural Products for Drug Discovery in the 21st Century: Innovations for Novel Drug Discovery. Int. J. Mol. Sci. 19 (6), 1578. 10.3390/ijms19061578 PMC6032166 29799486

[B172] Tomasz M. (1995). Mitomycin C: Small, Fast and Deadly (But Very Selective). Chem. Biol. 2 (9), 575–579. 10.1016/1074-5521(95)90120-5 9383461

[B173] Toumazi D. Constantinou C. (2020). A Fragile Balance: The Important Role of the Intestinal Microbiota in the Prevention and Management of Colorectal Cancer. Oncology 98 (9), 593–602. 10.1159/000507959 32604093

[B174] Trzoss L. Fukuda T. Costa-Lotufo L. V. Jimenez P. La Clair J. J. Fenical W. (2014). Seriniquinone, a Selective Anticancer Agent, Induces Cell Death by Autophagocytosis, Targeting the Cancer-Protective Protein Dermcidin. Proc. Natl. Acad. Sci. U. S. A. 111 (41), 14687–14692. 10.1073/pnas.1410932111 25271322 PMC4205641

[B175] Tuladhar A. Hondal R. J. Colon R. Hernandez E. L. Rein K. S. (2019). Effectors of Thioredoxin Reductase: Brevetoxins and Manumycin-A. Comp. Biochem. Physiol. C Toxicol. Pharmacol. 217, 76–86. 10.1016/j.cbpc.2018.11.015 30476593 PMC7485175

[B176] Turashvili G. Brogi E. (2017). Tumor Heterogeneity in Breast Cancer. Front. Med. (Lausanne) 4, 227. 10.3389/fmed.2017.00227 29276709 PMC5727049

[B177] Udwary D. W. Zeigler L. Asolkar R. N. Singan V. Lapidus A. Fenical W. (2007). Genome Sequencing Reveals Complex Secondary Metabolome in the Marine Actinomycete *Salinispora Tropica* . Proc. Natl. Acad. Sci. U. S. A. 104 (25), 10376–10381. 10.1073/pnas.0700962104 17563368 PMC1965521

[B178] Um S. Choi T. J. Kim H. Kim B. Y. Kim S. H. Lee S. K. (2013). Ohmyungsamycins A and B: Cytotoxic and Antimicrobial Cyclic Peptides Produced by Streptomyces Sp. From a Volcanic Island. J. Org. Chem. 78 (24), 12321–12329. 10.1021/jo401974g 24266328

[B179] Van Bergeijk D. A. Terlouw B. R. Medema M. H. Van Wezel G. P. (2020). Ecology and Genomics of Actinobacteria: New Concepts for Natural Product Discovery. Nat. Rev. Microbiol. 18 (10), 546–558. 10.1038/s41579-020-0379-y 32483324

[B180] Vippila M. R. Ly P. K. Cuny G. D. (2015). Synthesis and Antiproliferative Activity Evaluation of the Disulfide-Containing Cyclic Peptide Thiochondrilline C and Derivatives. J. Nat. Prod. 78 (10), 2398–2404. 10.1021/acs.jnatprod.5b00428 26444379

[B181] Wagg C. Bender S. F. Widmer F. van der Heijden M. G. (2014). Soil Biodiversity and Soil Community Composition Determine Ecosystem Multifunctionality. Proc. Natl. Acad. Sci. U. S. A. 111 (14), 5266–5270. 10.1073/pnas.1320054111 24639507 PMC3986181

[B182] Wang C. Du W. Lu H. Lan J. Liang K. Cao S. (2021). A Review: Halogenated Compounds from Marine Actinomycetes. Molecules 26 (9), 2754. 10.3390/molecules26092754 34067123 PMC8125187

[B183] Wang Q. Zhang Y. Wang M. Tan Y. Hu X. He H. (2017). Neo-actinomycins A and B, Natural Actinomycins Bearing the 5H-Oxazolo[4,5-B]phenoxazine Chromophore, from the Marine-Derived Streptomyces Sp. IMB094. Sci. Rep. 7 (1), 3591. 10.1038/s41598-017-03769-8 28620204 PMC5472614

[B184] Wang R. J. Zhang S. Y. Ye Y. H. Yu Z. Qi H. Zhang H. (2019). Three New Isoflavonoid Glycosides from the Mangrove-Derived Actinomycete *Micromonospora Aurantiaca* 110B. Mar. Drugs 17 (5), 294. 10.3390/md17050294 PMC6562861 31108876

[B185] Wang W. Kandimalla R. Huang H. Zhu L. Li Y. Gao F. (2019). Molecular Subtyping of Colorectal Cancer: Recent Progress, New Challenges and Emerging Opportunities. Semin. Cancer Biol. 55, 37–52. 10.1016/j.semcancer.2018.05.002 29775690 PMC6240404

[B186] Wang Z. Wen Z. Liu L. Zhu X. Shen B. Yan X. (2019). Yangpumicins F and G, Enediyne Congeners from *Micromonospora Yangpuensis* DSM 45577. J. Nat. Prod. 82 (9), 2483–2488. 10.1021/acs.jnatprod.9b00229 31490685 PMC7170010

[B187] Ward A. C. Bora N. (2006). Diversity and Biogeography of Marine Actinobacteria. Curr. Opin. Microbiol. 9 (3), 279–286. 10.1016/j.mib.2006.04.004 16675292

[B188] Weldon Gilcrease G. Stenehjem D. D. Wade M. L. Weis J. McGregor K. Whisenant J. (2019). Phase I/II Study of Everolimus Combined with mFOLFOX-6 and Bevacizumab for First-Line Treatment of Metastatic Colorectal Cancer. Invest. New Drugs 37 (3), 482–489. 10.1007/s10637-018-0645-2 30302599

[B189] Williams P. G. Miller E. D. Asolkar R. N. Jensen P. R. Fenical W. (2007). Arenicolides A-C, 26-membered Ring Macrolides from the Marine Actinomycete *Salinispora Arenicola* . J. Org. Chem. 72 (14), 5025–5034. 10.1021/jo061878x 17266372 PMC2577615

[B190] Wyche T. P. Dammalapati A. Cho H. Harrison A. D. Kwon G. S. Chen H. (2014). Thiocoraline Activates the Notch Pathway in Carcinoids and Reduces Tumor Progression *In Vivo* . Cancer Gene Ther. 21 (12), 518–525. 10.1038/cgt.2014.57 25412645 PMC4270822

[B191] Xiao F. Li H. Xu M. Li T. Wang J. Sun C. (2018). Staurosporine Derivatives Generated by Pathway Engineering in a Heterologous Host and Their Cytotoxic Selectivity. J. Nat. Prod. 81 (8), 1745–1751. 10.1021/acs.jnatprod.8b00103 30106291

[B192] Yamazaki Y. Kunimoto S. Ikeda D. (2007). Rakicidin A: A Hypoxia-Selective Cytotoxin. Biol. Pharm. Bull. 30 (2), 261–265. 10.1248/bpb.30.261 17268062

[B193] Yang F. X. Hou G. X. Luo J. Yang J. Yan Y. Huang S. X. (2018). New Phenoxazinone-Related Alkaloids from Strain *Streptomyces* Sp. KIB-H1318. J. Antibiot. (Tokyo) 71 (12), 1040–1043. 10.1038/s41429-018-0099-y 30218038

[B194] Ye X. Anjum K. Song T. Wang W. Yu S. Huang H. (2016). A New Curvularin Glycoside and its Cytotoxic and Antibacterial Analogues from Marine Actinomycete Pseudonocardia Sp. HS7. Nat. Prod. Res. 30 (10), 1156–1161. 10.1080/14786419.2015.1047775 26119337

[B195] Yuan G. Lin H. Wang C. Hong K. Liu Y. Li J. (2011). 1H and 13C Assignments of Two New Macrocyclic Lactones Isolated from *Streptomyces* Sp. 211726 and Revised Assignments of Azalomycins F3a, F4a and F5a. Magn. Reson Chem. 49 (1), 30–37. 10.1002/mrc.2697 21086419

[B196] Zhang S. Xie Q. Sun C. Tian X. P. Gui C. Qin X. (2019). Cytotoxic Kendomycins Containing the Carbacylic Ansa Scaffold from the Marine-Derived *Verrucosispora* Sp. SCSIO 07399. J. Nat. Prod. 82 (12), 3366–3371. 10.1021/acs.jnatprod.9b00654 31765156

[B197] Zhang Y. K. Zhang Q. Wang Y. L. Zhang W. Y. Hu H. Q. Wu H. Y. (2021). A Comparison Study of Age and Colorectal Cancer-Related Gut Bacteria. Front. Cell. Infect. Microbiol. 11, 606490. 10.3389/fcimb.2021.606490 33996615 PMC8121496

[B198] Zhao X. L. Wang H. Xue Z. L. Li J. S. Qi H. Zhang H. (2019). Two New Glutarimide Antibiotics from *Streptomyces* Sp. HS-NF-780. J. Antibiot. (Tokyo) 72 (4), 241–245. 10.1038/s41429-019-0143-6 30696946

[B199] Zheng D. Han L. Qu X. Chen X. Zhong J. Bi X. (2017). Cytotoxic Fusicoccane-type Diterpenoids from *Streptomyces Violascens* Isolated from *Ailuropoda Melanoleuca* Feces. J. Nat. Prod. 80 (4), 837–844. 10.1021/acs.jnatprod.6b00676 28206772

[B200] Zhou B. Hu Z. J. Zhang H. J. Li J. Q. Ding W. J. Ma Z. J. (2019). Bioactive Staurosporine Derivatives from the *Streptomyces* Sp. NB-A13. Bioorg Chem. 82, 33–40. 10.1016/j.bioorg.2018.09.016 30268972

[B201] Zhou H. Yuan Y. Wang H. Xiang W. Li S. Zheng H. (2021). Gut Microbiota: A Potential Target for Cancer Interventions. Cancer Manag. Res. 13, 8281–8296. 10.2147/CMAR.S328249 34764691 PMC8572730

[B202] Zhou Y. J. Zhao D. D. Liu H. Chen H. T. Li J. J. Mu X. Q. (2017). Cancer Killers in the Human Gut Microbiota: Diverse Phylogeny and Broad Spectra. Oncotarget 8 (30), 49574–49591. 10.18632/oncotarget.17319 28484095 PMC5564789

