# Supplementary material for: Natural Products from Actinobacteria as a Potential Source of New Therapies Against Colorectal Cancer: A Review
Source: Front Pharmacol. 2022 Jul 11;13:929161. doi: 10.3389/fphar.2022.929161 (PMC9310018; doi:10.3389/fphar.2022.929161)
Supplement: Supplementary file 1 [file Table1.docx]

Table S1. The structure of NPs with anti-CRC properties from marine actinobacteria and their mechanism of actions and origins

| *Bacteria* | Origin of bacteria | Compound  name | Chemical Structure | Colorectal Cell line | Special property | country of origin | references |
| --- | --- | --- | --- | --- | --- | --- | --- |
| *Salinispora Tropica* CNB-392 | sediment sample- depth of about 1 m- mangrove environment | Salinosporamide A (1) (Marizomib, NPI-0052) | Indoles | HCT-116, HCC-2998, xenograft model of colon cancer | 20s proteasome inhibition | Chub Cay, Bahamas | (1-3) |
| *Actinomadura Sp.* M048 | bay sediments | Questiomycin A (2), N-acetylquestiomycin A (3) and Chandrananimycin A (4), B (5) | Oxazines | HT-29 | - | Jiaozhou Bay, China | (4) |
| *Streptomyces Strain* BL-49-58-005 | unidentified marine invertebrate | 3,6-Disubstituted indole 2  (Aldoxime) (6) | Benzopyrrole, Indoles | LOVO and LOVO-DOX | - | Mexico | (5) |
| *Salinispora Arenicola* CNT-088 | marine sediment- depth of ca. 20 m off the Great Astrolabe Reef | Arenamides A (7) and B (8) | Peptides | HCT-116 | Blocking TNF induction activation and NFkB inhibition | Kandavu Island chain, Fiji | (6) |
| *Salinispora Arenicola Strain* CNR-005 | marine sediment- depth of approximately 30 m | Arenicolide A (9) | Polyketide  /Macrolide, Lactones | HCT-116 | - | Guam | (7) |
| *Streptomyces Aureo-Verticillatus* NPS001583 | marine sediment | Aureoverticillactam (10) | Polyketide  /Macrolide, Lactones | HT-29 | - | - | (8) |
| *Streptomyces CNQ-525* | ocean sediments- depth of 152 m | Dihydroquinones 1, 2 and 4 (11-13) | Hydroquinones, Other hydrocarbones | HCT-116 | - | La Jolla, California | (9) |
| *Salinispora* *Pacifica* Strain CNS103 | Sediments- depth of 500 m | Cyanosporaside A (14) | Indene, Glycosides | HCT-116 | - | Palau | (10) |
| *Streptomyces Strain* CNQ-085 | marine sediment- depth of ca.  50 m | Daryamides (Especially Type A) (15) | Polyketide/Polyene, Other hydrocarbones | HCT-116 | - | San Diego, California | (11) |
| *Streptomyces Sp. Isolate* B8652 | ediment of  the Laguna de Terminos | Trioxacarcins A-D (16-19)  and Gutingimycin (20) | Aminoglycoside, Glycosides | HT-29 | - | Gulf of Mexico | (12, 13) |
| *Actinomadura Sp* BL-42-PO13-046 | - | IB-00208 (21) | Xanthene, Glycosides | HT-29 | - | the northern  coast of Spain | (14, 15) |
| *Micromonospora Sp.* L-25-ES25-008 | from a sponge- at the Indian Ocean | IB-96212 (22) | Polyketide  /Macrolide, Lactones | HT-29 | - | near the coast of  Mozambique | (16, 17) |
| *Nocardiopsis Lucentensis* Strain CNR-712 | from sediment -from a  shallow saline pond | Lucentamycins  A (23) and B (24) | Peptides | HCT-116 | - | island of Little San Salvador, in the  Bahamas | (18) |
| *Streptomyces* CNH-099 | sediment sample- at −1 m | Neomarinone (25), Isomarinone (26), Hydroxydebromomarinone (27), and Methoxydebromomarinone (28) | Other hydrocarbones | HCT-116 | - | Batiquitos Lagoon, North of San Diego, California | (19, 20) |
| *Streptomyces* Strain CNQ-617 | marine sediment | Marineosins A (29) and B (30) | Pyrrole, Azoles | HCT-116 | - | - | (21) |
| *Streptomyces* Strain CNH990 | marine sediment | Marmycins A (31) and B (32) | Anthraquinone, Quinones | HCT-116 | - | Sea of Cortez, Baja California Sur, México | (22) |
| *Streptomyces Sp.* YM14*-*060 | unidentified greenish ascidians- depth of 0.5 m | Piericidin A (33) | Pyridines | HT-29 | - | Iwayama Bay, Palau | (23) |
| *Streptomyces Sp. Strain* CNQ 593 | marine sediments | Piperazimycins A-C (34-36) | Depsipeptide, Peptides | HCT-116 |  | near the island of Guam | (24) |
| *Micromonospora Sp.* L-31-CLCO-002 | sponge Clathrina coriacea collected on the coast of  Fuerteventura Island | Staurosporines analoges  4'-N-Methyl-5'-Hydroxystaurosporine (37) and 5'-Hydroxystaurosporine (38) | Indole Alkaloid, Alkaloids | HT-29 | - | Canary Islands archipielago | (25-27) |
| *Streptomyces Sp.* NB-A13 | marine sediments | Staurosporine derivatives 1-14 (39-52) | Indole Alkaloid, Alkaloids | SW-620 | - | Ningbo  city (Zhejiang province, China) | (28) |
| *Streptomyces Coelicolor* M1146 | - | Staurosporine M1 (53),  Staurosporine M2 (54) | Indole Alkaloid, Alkaloids | HCT-116 | - | - | (29) |
| *Streptomyces Sp.* Kordi-3238 | deep-sea sediment | Streptokordin (55) | Pyridines | HCT-15 | - | Ayu Trough- western Pacific Ocean | (30) |
| *Micromonospora (Marina) Sp.* L-13-ACM2-092 | marine  soft coral | Thiocoraline (56) | Depsipeptide, Peptides | HT-29  LOVO  SW620 | Inhibition of DNA polymerase α -  inducing  activation  of  Notch pathway | Indian Ocean near the coast  of Mozambique | (31, 32) |
| *Previously Isolated from Verrucisispora Sp*. (Strain WMMA107) | sponge Chondrilla caribensis f. caribensis | Derivative No. 22 of thiochondrilline C (57) | Depsipeptide, Peptides | 7 different types | - | Florida Keys (24° 39’ 17.90”, 81° 17’ 51.09”), United States | (33) |
| *Streptomyces* Strain M491 | sand sample | 15-Hydroxy-T-Muurolol (58) | Sesquiterpenes, Terpenes | HCT-116, HT-29 | - | Qingdao coast (China) | (34) |
| *Streptomyces Sp.* MDG-04-17-069 | marine sediments | Tartrolon D (59) | Macrodiolide, Lactones | HT-29 | - | near  the east coast of Madagascar | (35) |
| *Streptomyces Sp.* SCA29 | - | 4-Methoxyacetanilide (60) | Acetamide, Amides | HT-29 | - | Havelock Island, Andaman and Nicobar Islands, India | (36) |
| *Micromonospora* Strain FIM07-0019 | shallow coastal waters | Levantilide C (61) | Polyketide  /Macrolide, Lactones | SW620 | - | near the island of Chiloe, Chile | (37) |
| *Actinoalloteichus Cyanogriseus* WH1-2216-6 And Its Mutant Species | marine sediments collected  from the seashore | Cyanogrisides F (62) and G (63) | Bipyridine, Glycosides | HCT-116 | - | Weihai, China | (38) |
| *Amycolatopsis Sp* | sponge sample | Amycolactam (64) | Indoles | HCT-116 | - | Micronesia | (39) |
| *Microbacterium Sediminis* Spp. Nov. YLB-01 | sediment sample  - at 2327 m water depth | Microbacterin B (65) | Peptides | HCT-8 | - | (49.8405u E  37.8111u S) in the south-west Indian Ocean | (40) |
| *Pseudonocardia Sp.* HS7 | cloacal aperture of sea cucumber Holothuria moebii | Curvularin macrolides (66-70) and their three synthetic acyl derivatives (5a-5c) (71-73) | Lactones | HCT-15, SW620 | - | China | (41) |
| *Streptomyces Caniferus* GUA-06-05-006A | marine polychaete, Filograna sp. | PM100117 (74) And PM100118 (75) | Polyketide  /Macrolide, Lactones | HT-29 | Altering the permeability and integrity of cell membranes | near  Guadalupe Island in the Pacific Ocean | (42) |
| *Streptomyces Sp.* 112CH148 | crown-of-thorns starfish, Acanthaster planci | Violapyrones B (76), C (77), H (78) and I (79) | Pyrone, Pyrans | HCT-116, HCT-15 | - | Chuuk, Federated States of Micronesia | (43) |
| *Nocardia Dassonvillei* BM-17 | marine sediments | N- (2-hydroxyphenyl) -2-phenazinamine (NHP) (80) | Phenazines | HCT-116 | - | Arctic Ocean (78°55′N, 11°56′E) | (44) |
| *Streptomyces Sp.* (NPS008187) | marine sediments | Glaciapyrrole A (81) | Sesquiterpene, Terpenes | HT-29 | - | Alaska | (45) |
| *Streptomyces Sp.* Strain HKI0708 | extreme and  unusual habitats | Elaiomycins D-F (82-84) | Azoxyalkene, Azo Compounds | HT-29 | - | Yunnan Province in the southwest of China | (46) |
| *Streptomyces Sp.* Strain HB202 | marine sponge Halichondria panicea | Mayamycin (85) | Benz(A)Anthracene, Other hydrocarbones | HT-29 | - | Baltic Sea | (47) |
| *Streptomyces* CNQ-418 | marine sediment | Marinopyrrole A*-F (86-91) | Pyrrole, Azoles | HCT-116 | MCL-1 inhibitor* | La Jolla, California | (48, 49) |
| *Saccharomonospora* CNQ-490 | marine sediment | Lodopyridone (92) | Alkaloids | HCT-116 | - | mouth of the La Jolla Submarine Canyon, California | (50) |
| *Streptomyces* CNR-698 | bottom sediments- depth of 1618 meters | Ammosamide A (93) and B (94) | Amides |  |  | Bahamas Islands |  |
| *Streptomyces Variabilis* SNA-020 | marine sediment | Ammosamide D (95) | Amides | HCT-116 | specific targeting a member of the myosin family | Sweetings Cay, Bahamas | (51-53) |
| *Streptomyces Malaysiensis* CNQ-509 | marine sediment- depth of 44 m | Nitropyrrolin A (96), (97) and D (98) | Sesquiterpenoid,  Terpenes | HCT-116 | - | offshore of La Jolla, California | (54) |
| *Streptomyces Strain* CNH-287 | marine sediment | Chlorizidine (99) | Benzenediol, Alkaloids | HCT-116 | - | intertidal zone near San Clemente, California | (55) |
| *Streptomyces MAR4* CNY-960 And CNS-284 | marine sediments | Marinocyanin A – F (100-105) | Phenazines | HCT-116 | - | Solomon Islands and in Palau, respectively | (56) |
| *Streptomyces Sp.* SCSIO 11791 | marine sediments | Dionemycin (106) and 6-ome-7 ', 7 ″ -dichorochromopyrrolic Acid (107) | Alkaloids | HCT-116 | - | South China Sea | (57) |
| *Streptomyces Strain* CNQ-329 And CNH-070 | marine sediments | Napyradiomycins A – F (108-113) and B2-B4 (114-116) | Naphthoquinones, Quinones |  |  | San Diego, California –  mouth of San Elijo Lagoon in Encinitas, California |  |
| *Streptomyces* CNQ-525 | marine sediment-depth of 152 m | Napyradiomycin CNQ525.510B, CNQ525.538, CNQ525.554, CNQ525.600 (117-120), Also  A80915A, A80915B, A80915C, A80915D, CNQ525.512, And SF24 (121-126) | Naphthoquinones, Quinones | HCT-116 | - | coast of La Jolla, California | (58, 59) |
| *Streptomyces* Strain ART 5 | surface sediment sample | Chromophore-V (127) | Benzoxazine, Oxazines | HCT-116 | - | East Siberian continental margin (75° 22.2743′ N, 177° 17.4846′ E) of Arctic Ocean | (60) |
| *Micromonospora Sp.* | Eudistoma vannamei, a  Brazilian endemic ascidian | 4,6,11-Trihydroxy-9-propyltetracene-5,12-dione  and  10β-Carbomethoxy-7, 8,9,10-tetrahydro-4,6,7α 9α, 11-pentahydroxy-9-propyltetracene- 5,12-dione (128-129) | Anthracyclinones, Glycosides | HCT-8 | - | coast of northeastern Brazil- Taibá  Beach, Ceara | (61) |
| *Micromonospora Sp. TP-*A0468 | seawater sample | Kosinostatin (130) | Fluorene, Other hydrocarbones | HCT-116, HCT-15, HT-29, KM12, and HCC2998 | Inhibition of human DNA topoisomerases I and IIα | toyama bay, japan | (62) |
| *Micromonospora Lomaivitiensis* | inner  core of marine ascidian Polysyncraton lithostrotum | Lomaiviticin A (131) | Fluorene, Other hydrocarbones | HCT-15, CACO2, SW948, COLO205 | Potentially DNA-damaging | - | (63) |
| *Micromonospora Aurantiaca* 110B | rhizosphere soil of mangrove plants | Daidzein-4'-(2-Deoxy-Α-L-Fucopyranoside), Daidzein-7-(2-Deoxy-Α-L-Fucopyranoside) And Daidzein-4',7-Di-(2-Deoxy-Α-L-Fucopyranoside) (132-134) | Isoflavonoid, Glycoside | HCT-116 | - | Fujian province, China | (64) |
| Marine Bacterium Closely Related to *Streptomyces* | marine sediments | Actinoranone (135) | Terpenes | HCT-116 | - | coast of southern California | (65) |
| *Actinoalloteichus Hymeniacidonis* 179DD-027 | marine sediments | Dokdolipids A-C (136-138) | Rhamnolipid, Glycolipides | HCT-15 | - | coasts of Dokdo island, Republic of Korea | (66) |
| *Micromonospora Yangpuensis* DSM 45577 | cup-shaped sponge | Yangpumicins A, F, G (139-141) | Anthraquinone, Enediynes | Caco-2 and SKBR-3 | - | Dachan reef, Yangpu in the South China Sea | (67) |
| *Saccharomonospora Sp.* UR22 And *Dietziaceae Dietzia Sp.* UR66 | Red Sea sponge Callyspongia siphonella | Saccharomonosporine A (142) And One Induced Metabolite (143) | Brominated Oxo-Indole, Alkaloids | HT-29 | Pim-1 kinase inhibition | depth of 10 m in the Red Sea (Hurghada, Egypt) | (68) |
| *Streptomyces Sp.* HNA39 | marine sediments | A Cyclizidine Alkaloid  Compound Number 2 (144) | Alkaloids | HCT-116 | ROCK2 protein kinase inhibition | Hainan Island (Hainan  province, China) | (69) |
| *Salinispora arenicola strain CNR-647* | sample of the ascidian Ecteinascidia turbinata | Arenimycin (145) | Tetracenequinone, Glycosides | HCT-116 | Strong inhibitory effect on cell division | mangrove channel at Sweetings Cay, Grand Bahama Island (tropical Atlantic Ocean) | (70) |
| *Streptomyces Sp.* XMA39 | - | Strepoxepinmycins C and D (146-147) | Naphthoquinone, Quinones | HCT-116 | - | - | (71) |
| *Verrucosispora Sp.* SCSIO 07399 | marine sediments | Kendomycins B, C and D (148-150) | Macrocyclic Lactam, Lactones | RKO | - | northern South China  Sea | (72) |
| *Streptomyces nigra sp. nov.* | from the rhizosphere soil of the mangrove | cyclo (Pro-Phe) (151), cyclo (Pro-Ala) (152), cyclo (Pro-Val) (153), and cyclo (Pro-Leu) (154) | Peptides | HCT-116 | - | Avicennia marina in China | (73) |
| *Streptomyces sp. VN1.* | coastal region | unique furan-type compound (155) | - | HCT-116 | - | Phu Yen Province (central Viet Nam) | (74) |
| *Streptomyces sp. SBT348* | Mediterranean sponge Petrosia ficiformis | petrocidin A (156); 2,3-dihydroxybenzamide (157) | Polyketides, Amides | HT-29 | Prevention of overexpression of microsomal prostaglandin E2 synthase-1 | Milos, Greece | (75) |
| *Streptomyces cacaoi 14CM034* | sediment sample, at a depth of 8m | K41 A (158) and compound (159) | polyether-type polyketides | Caco-2 | inhibiting autophagy and inducing apoptosis | Mersin Coastline, Turkey | (75, 76) |
| *Streptomyces sp. IMB094* | marine sediment sample at a depth of ca. 40 m | neo-actinomycin A (160), neo-actinomycin B (161), actinomycin D (162), and actinomycin X2 (163) | peptides | HCT116 | - | Heishijiao Bay, Dalian, China | (77) |
| *Streptomyces sp. SNJ042* | from a sand beach | Ohmyungsamycin A (164) | cyclic peptide | HCT116 | caspase-mediated apoptosis and reducing the expression of Skp2 | Jeju, a volcanic island in the Republic of Korea | (73, 74, 78, 79) |
| *Nocardiopsis,* Strain CNT-189 | shore sediment from the surf zone | Androsamide (208) | Cyclic Tetrapeptide, Peptides | HCT-116, Caco-2 | Anti-invasion and migration effects | Bahamas | (80) |
| *Streptomyces. Albogriseolus* A2002 | marine sediments | Echinosporin and 7-Deoxyechinosporin (209-210) | Lactones | HCT-15 | Apoptosis and cell cycle arrest | Jiaozhou Bay, China | (81) |
| *Streptomyces Sp.* M045 | marine sediment | Manumycin A (213) | Polyketide/Polyene, Other hydrocarbones | COLO320-DM | Inhibition of Ras farnesyl transfrase | Jiaozhou Bay in China | (82, 83) (84, 85) |
| *Micromonospora Sp*. Strain TP-A0316 | seawater sample | Arisostatin A (214) | Aminoglycoside, Glycosides | HCC2998 | Onset of apoptosis  due  to  activation of Caspase-3 and  ROS species formation | Toyama Bay, Japan | (86) |
| *Ornithinimicrobiaceae Serinicoccus Sp* | - | Seriniquinone (215) | Quinones | HCT-116, HCT-15 | Autophagy and apoptosis | - | (87) |
| *Streptomyces Sp.* WBF16 | marine sediments | Chromomycin B, A2*, A3 (216-218) | Anthraquinone, Quinonea | HCT-116 | Autophagy* | Bijiatuan, in the city of Weihai, China | (88, 89) |
| *Micromonospora Chalcea* FIM 02–523 | Fujian  Institute of Microbiology | Rakicidins E (223) And G-I (220-222) | Cyclic Depsipeptide, Peptides | HCT-8 | Angiogenesis and hypoxia | - | (90) |
|  |  |  |  |  |  |  |  |
|  |  |  |  |  |  |  |  |

**References**

1. Feling RH, Buchanan GO, Mincer TJ, Kauffman CA, Jensen PR, Fenical W. Salinosporamide A: a highly cytotoxic proteasome inhibitor from a novel microbial source, a marine bacterium of the new genus *Salinospora*. Angew Chem Int Ed Engl. 2003;42(3):355-7.

2. Jensen PR, Williams PG, Oh DC, Zeigler L, Fenical W. Species-specific secondary metabolite production in marine actinomycetes of the genus *Salinispora*. Appl Environ Microbiol. 2007;73(4):1146-52.

3. Udwary DW, Zeigler L, Asolkar RN, Singan V, Lapidus A, Fenical W, et al. Genome sequencing reveals complex secondary metabolome in the marine actinomycete *Salinispora tropica*. Proc Natl Acad Sci U S A. 2007;104(25):10376-81.

4. Maskey RP, Li F, Qin S, Fiebig HH, Laatsch H. Chandrananimycins A approximately C: Production of novel anticancer antibiotics from a marine *Actinomadura* sp. isolate M048 by variation of medium composition and growth conditions. J Antibiot (Tokyo). 2003;56(7):622-9.

5. Sánchez López JM, Martínez Insua M, Pérez Baz J, Fernández Puentes JL, Cañedo Hernández LM. New cytotoxic indolic metabolites from a marine *Streptomyces*. Journal of natural products. 2003;66(6):863-4.

6. Asolkar RN, Freel KC, Jensen PR, Fenical W, Kondratyuk TP, Park EJ, et al. Arenamides A-C, cytotoxic NFkappaB inhibitors from the marine actinomycete *Salinispora arenicola*. J Nat Prod. 2009;72(3):396-402.

7. Williams PG, Miller ED, Asolkar RN, Jensen PR, Fenical W. Arenicolides A-C, 26-membered ring macrolides from the marine actinomycete *Salinispora arenicola*. J Org Chem. 2007;72(14):5025-34.

8. Mitchell SS, Nicholson B, Teisan S, Lam KS, Potts BC. Aureoverticillactam, a novel 22-atom macrocyclic lactam from the marine actinomycete *Streptomyces aureoverticillatus*. J Nat Prod. 2004;67(8):1400-2.

9. Soria-Mercado IE, Prieto-Davo A, Jensen PR, Fenical W. Antibiotic terpenoid chloro-dihydroquinones from a new marine actinomycete. J Nat Prod. 2005;68(6):904-10.

10. Oh DC, Williams PG, Kauffman CA, Jensen PR, Fenical W. Cyanosporasides A and B, chloro- and cyano-cyclopenta[a]indene glycosides from the marine actinomycete "*Salinispora pacifica*". Org Lett. 2006;8(6):1021-4.

11. Asolkar RN, Jensen PR, Kauffman CA, Fenical W. Daryamides A-C, weakly cytotoxic polyketides from a marine-derived actinomycete of the genus *Streptomyces* strain CNQ-085. J Nat Prod. 2006;69(12):1756-9.

12. Maskey RP, Helmke E, Kayser O, Fiebig HH, Maier A, Busche A, et al. Anti-cancer and antibacterial trioxacarcins with high anti-malaria activity from a marine *Streptomycete* and their absolute stereochemistry. J Antibiot (Tokyo). 2004;57(12):771-9.

13. Maskey RP, Sevvana M, Usón I, Helmke E, Laatsch H. Gutingimycin: A highly complex metabolite from a marine *Streptomycete*. Angew Chem Int Ed Engl. 2004;43(10):1281-3.

14. Malet-Cascón L, Romero F, Espliego-Vázquez F, Grávalos D, Fernández-Puentes JL. IB-00208, a new cytotoxic polycyclic xanthone produced by a marine-derived *Actinomadura*. I. Isolation of the strain, taxonomy and biological activites. J Antibiot (Tokyo). 2003;56(3):219-25.

15. Rodríguez JC, Fernández Puentes JL, Baz JP, Cañedo LM. IB-00208, a new cytotoxic polycyclic xanthone produced by a marine-derived *Actinomadura*. II. Isolation, physico-chemical properties and structure determination. J Antibiot (Tokyo). 2003;56(3):318-21.

16. Cañedo LM, Fernández-Puentes JL, Baz JP. IB-96212, a novel cytotoxic macrolide produced by a marine *Micromonospora*. II. Physico-chemical properties and structure determination. J Antibiot (Tokyo). 2000;53(5):479-83.

17. Fernández-Chimeno RI, Cañedo L, Espliego F, Grávalos D, De La Calle F, Fernández-Puentes JL, et al. IB-96212, a novel cytotoxic macrolide produced by a marine *Micromonospora*. I. Taxonomy, fermentation, isolation and biological activities. J Antibiot (Tokyo). 2000;53(5):474-8.

18. Cho JY, Williams PG, Kwon HC, Jensen PR, Fenical W. Lucentamycins A-D, cytotoxic peptides from the marine-derived actinomycete *Nocardiopsis lucentensis*. J Nat Prod. 2007;70(8):1321-8.

19. Hardt IH, Jensen PR, Fenical W. Neomarinone, and new cytotoxic marinone derivatives, produced by a marine filamentous bacterium (actinomycetales). Tetrahedron Letters. 2000;41(13):2073-6.

20. Kalaitzis JA, Hamano Y, Nilsen G, Moore BS. Biosynthesis and structural revision of neomarinone. Org Lett. 2003;5(23):4449-52.

21. Boonlarppradab C, Kauffman CA, Jensen PR, Fenical W. Marineosins A and B, cytotoxic spiroaminals from a marine-derived actinomycete. Org Lett. 2008;10(24):5505-8.

22. Martin GD, Tan LT, Jensen PR, Dimayuga RE, Fairchild CR, Raventos-Suarez C, et al. Marmycins A and B, cytotoxic pentacyclic C-glycosides from a marine sediment-derived actinomycete related to the genus *Streptomyces*. J Nat Prod. 2007;70(9):1406-9.

23. Hwang JH, Kim JY, Cha MR, Ryoo IJ, Choo SJ, Cho SM, et al. Etoposide-resistant HT-29 human colon carcinoma cells during glucose deprivation are sensitive to piericidin A, a GRP78 down-regulator. J Cell Physiol. 2008;215(1):243-50.

24. Miller ED, Kauffman CA, Jensen PR, Fenical W. Piperazimycins: cytotoxic hexadepsipeptides from a marine-derived bacterium of the genus *Streptomyces*. J Org Chem. 2007;72(2):323-30.

25. Hernández LM, Blanco JA, Baz JP, Puentes JL, Millán FR, Vázquez FE, et al. 4'-N-methyl-5'-hydroxystaurosporine and 5'-hydroxystaurosporine, new indolocarbazole alkaloids from a marine *Micromonospora* sp. strain. J Antibiot (Tokyo). 2000;53(9):895-902.

26. Itoh T, Kinoshita M, Aoki S, Kobayashi M. Komodoquinone A, a novel neuritogenic anthracycline, from marine *Streptomyces* sp. KS3. J Nat Prod. 2003;66(10):1373-7.

27. Omura S, Iwai Y, Hirano A, Nakagawa A, Awaya J, Tsuchya H, et al. A new alkaloid AM-2282 of *Streptomyces* origin. Taxonomy, fermentation, isolation and preliminary characterization. J Antibiot (Tokyo). 1977;30(4):275-82.

28. Zhou B, Hu ZJ, Zhang HJ, Li JQ, Ding WJ, Ma ZJ. Bioactive staurosporine derivatives from the *Streptomyces* sp. NB-A13. Bioorg Chem. 2019;82:33-40.

29. Xiao F, Li H, Xu M, Li T, Wang J, Sun C, et al. Staurosporine Derivatives Generated by Pathway Engineering in a Heterologous Host and Their Cytotoxic Selectivity. J Nat Prod. 2018;81(8):1745-51.

30. Jeong SY, Shin HJ, Kim TS, Lee HS, Park SK, Kim HM. Streptokordin, a new cytotoxic compound of the methylpyridine class from a marine-derived *Streptomyces* sp. KORDI-3238. J Antibiot (Tokyo). 2006;59(4):234-40.

31. Erba E, Bergamaschi D, Ronzoni S, Faretta M, Taverna S, Bonfanti M, et al. Mode of action of thiocoraline, a natural marine compound with anti-tumour activity. Br J Cancer. 1999;80(7):971-80.

32. Romero F, Espliego F, Pérez Baz J, García de Quesada T, Grávalos D, de la Calle F, et al. Thiocoraline, a new depsipeptide with antitumor activity produced by a marine *Micromonospora*. I. Taxonomy, fermentation, isolation, and biological activities. J Antibiot (Tokyo). 1997;50(9):734-7.

33. Vippila MR, Ly PK, Cuny GD. Synthesis and Antiproliferative Activity Evaluation of the Disulfide-Containing Cyclic Peptide Thiochondrilline C and Derivatives. J Nat Prod. 2015;78(10):2398-404.

34. Ding L, Pfoh R, Rühl S, Qin S, Laatsch H. T-muurolol sesquiterpenes from the marine *Streptomyces* sp. M491 and revision of the configuration of previously reported amorphanes. J Nat Prod. 2009;72(1):99-101.

35. Pérez M, Crespo C, Schleissner C, Rodríguez P, Zúñiga P, Reyes F. Tartrolon D, a cytotoxic macrodiolide from the marine-derived actinomycete *Streptomyces* sp. MDG-04-17-069. J Nat Prod. 2009;72(12):2192-4.

36. Siddharth S, Vittal RR. Isolation, characterization, and structural elucidation of 4-methoxyacetanilide from marine actinobacteria *Streptomyces* sp. SCA29 and evaluation of its enzyme inhibitory, antibacterial, and cytotoxic potential. Arch Microbiol. 2019;201(6):737-46.

37. Fei P, Chuan-Xi W, Yang X, Hong-Lei J, Lu-Jie C, Uribe P, et al. A new 20-membered macrolide produced by a marine-derived *Micromonospora* strain. Nat Prod Res. 2013;27(15):1366-71.

38. Fu P, Zhu Y, Mei X, Wang Y, Jia H, Zhang C, et al. Acyclic congeners from *Actinoalloteichus cyanogriseus* provide insights into cyclic bipyridine glycoside formation. Org Lett. 2014;16(16):4264-7.

39. Kwon Y, Kim SH, Shin Y, Bae M, Kim BY, Lee SK, et al. A new benzofuran glycoside and indole alkaloids from a sponge-associated rare actinomycete, *Amycolatopsis* sp. Mar Drugs. 2014;12(4):2326-40.

40. Liu D, Lin H, Proksch P, Tang X, Shao Z, Lin W. Microbacterins A and B, new peptaibols from the deep sea actinomycete *Microbacterium sediminis* sp. nov. YLB-01(T). Org Lett. 2015;17(5):1220-3.

41. Ye X, Anjum K, Song T, Wang W, Yu S, Huang H, et al. A new curvularin glycoside and its cytotoxic and antibacterial analogues from marine actinomycete *Pseudonocardia* sp. HS7. Nat Prod Res. 2016;30(10):1156-61.

42. Pérez M, Schleissner C, Fernández R, Rodríguez P, Reyes F, Zuñiga P, et al. PM100117 and PM100118, new antitumor macrolides produced by a marine *Streptomyces caniferus* GUA-06-05-006A. J Antibiot (Tokyo). 2016;69(5):388-94.

43. Shin HJ, Lee HS, Lee JS, Shin J, Lee MA, Lee HS, et al. Violapyrones H and I, new cytotoxic compounds isolated from *Streptomyces* sp. associated with the marine starfish *Acanthaster planci*. Mar Drugs. 2014;12(6):3283-91.

44. Gao X, Lu Y, Xing Y, Ma Y, Lu J, Bao W, et al. A novel anticancer and antifungus phenazine derivative from a marine actinomycete BM-17. Microbiol Res. 2012;167(10):616-22.

45. Macherla VR, Liu J, Bellows C, Teisan S, Nicholson B, Lam KS, et al. Glaciapyrroles A, B, and C, pyrrolosesquiterpenes from a *Streptomyces* sp. isolated from an Alaskan marine sediment. J Nat Prod. 2005;68(5):780-3.

46. Ding L, Ndejouong Ble S, Maier A, Fiebig HH, Hertweck C. Elaiomycins D-F, antimicrobial and cytotoxic azoxides from *Streptomyces* sp. strain HKI0708. J Nat Prod. 2012;75(10):1729-34.

47. Schneemann I, Kajahn I, Ohlendorf B, Zinecker H, Erhard A, Nagel K, et al. Mayamycin, a cytotoxic polyketide from a Streptomyces strain isolated from the marine sponge Halichondria panicea. J Nat Prod. 2010;73(7):1309-12.

48. Doi K, Li R, Sung SS, Wu H, Liu Y, Manieri W, et al. Discovery of marinopyrrole A (maritoclax) as a selective Mcl-1 antagonist that overcomes ABT-737 resistance by binding to and targeting Mcl-1 for proteasomal degradation. J Biol Chem. 2012;287(13):10224-35.

49. Hughes CC, Prieto-Davo A, Jensen PR, Fenical W. The marinopyrroles, antibiotics of an unprecedented structure class from a marine *Streptomyces* sp. Org Lett. 2008;10(4):629-31.

50. Maloney KN, Macmillan JB, Kauffman CA, Jensen PR, Dipasquale AG, Rheingold AL, et al. Lodopyridone, a structurally unprecedented alkaloid from a marine actinomycete. Org Lett. 2009;11(23):5422-4.

51. Hughes CC, MacMillan JB, Gaudêncio SP, Fenical W, La Clair JJ. Ammosamides A and B target myosin. Angew Chem Int Ed Engl. 2009;48(4):728-32.

52. Hughes CC, MacMillan JB, Gaudêncio SP, Jensen PR, Fenical W. The ammosamides: structures of cell cycle modulators from a marine-derived *Streptomyces* species. Angew Chem Int Ed Engl. 2009;48(4):725-7.

53. Pan E, Jamison M, Yousufuddin M, MacMillan JB. Ammosamide D, an oxidatively ring opened ammosamide analog from a marine-derived *Streptomyces variabilis*. Org Lett. 2012;14(9):2390-3.

54. Kwon HC, Espindola APDM, Park J-S, Prieto-Davó A, Rose M, Jensen PR, et al. Nitropyrrolins A-E, cytotoxic farnesyl-α-nitropyrroles from a marine-derived bacterium within the actinomycete family Streptomycetaceae. Journal of natural products. 2010;73(12):2047-52.

55. Alvarez-Mico X, Jensen PR, Fenical W, Hughes CC. Chlorizidine, a cytotoxic 5H-pyrrolo[2,1-a]isoindol-5-one-containing alkaloid from a marine *Streptomyces* sp. Org Lett. 2013;15(5):988-91.

56. Asolkar RN, Singh A, Jensen PR, Aalbersberg W, Carté BK, Feussner KD, et al. Marinocyanins, cytotoxic bromo-phenazinone meroterpenoids from a marine bacterium from the *Streptomycete* clade MAR4. Tetrahedron. 2017;73(16):2234-41.

57. Song Y, Yang J, Yu J, Li J, Yuan J, Wong NK, et al. Chlorinated bis-indole alkaloids from deep-sea derived *Streptomyces* sp. SCSIO 11791 with antibacterial and cytotoxic activities. J Antibiot (Tokyo). 2020;73(8):542-7.

58. Cheng YB, Jensen PR, Fenical W. Cytotoxic and antimicrobial napyradiomycins from two marine-derived, MAR 4 *Streptomyces* Strains. European J Org Chem. 2013;2013(18).

59. Farnaes L, Coufal NG, Kauffman CA, Rheingold AL, DiPasquale AG, Jensen PR, et al. Napyradiomycin derivatives, produced by a marine-derived actinomycete, illustrate cytotoxicity by induction of apoptosis. J Nat Prod. 2014;77(1):15-21.

60. Moon K, Ahn CH, Shin Y, Won TH, Ko K, Lee SK, et al. New benzoxazine secondary metabolites from an arctic actinomycete. Mar Drugs. 2014;12(5):2526-38.

61. Sousa Tda S, Jimenez PC, Ferreira EG, Silveira ER, Braz-Filho R, Pessoa OD, et al. Anthracyclinones from *Micromonospora* sp. J Nat Prod. 2012;75(3):489-93.

62. Furumai T, Igarashi Y, Higuchi H, Saito N, Oki T. Kosinostatin, a quinocycline antibiotic with antitumor activity from *Micromonospora* sp. TP-A0468. J Antibiot (Tokyo). 2002;55(2):128-33.

63. He H, Ding WD, Bernan VS, Richardson AD, Ireland CM, Greenstein M, et al. Lomaiviticins A and B, potent antitumor antibiotics from *Micromonospora lomaivitiensis*. J Am Chem Soc. 2001;123(22):5362-3.

64. Wang RJ, Zhang SY, Ye YH, Yu Z, Qi H, Zhang H, et al. Three new isoflavonoid glycosides from the mangrove-derived actinomycete *Micromonospora aurantiaca* 110B. Mar Drugs. 2019;17(5).

65. Nam SJ, Kauffman CA, Paul LA, Jensen PR, Fenical W. Actinoranone, a cytotoxic meroterpenoid of unprecedented structure from a marine adapted *Streptomyces* sp. Org Lett. 2013;15(21):5400-3.

66. Choi BK, Lee HS, Kang JS, Shin HJ. Dokdolipids a-c, hydroxylated rhamnolipids from the marine-derived actinomycete *actinoalloteichus hymeniacidonis*. Mar Drugs. 2019;17(4).

67. Wang Z, Wen Z, Liu L, Zhu X, Shen B, Yan X, et al. Yangpumicins F and G, Enediyne Congeners from *Micromonospora yangpuensis* DSM 45577. J Nat Prod. 2019;82(9):2483-8.

68. El-Hawary SS, Sayed AM, Mohammed R, Khanfar MA, Rateb ME, Mohammed TA, et al. New Pim-1 Kinase Inhibitor From the Co-culture of Two Sponge-Associated Actinomycetes. Front Chem. 2018;6:538.

69. Jiang YJ, Li JQ, Zhang HJ, Ding WJ, Ma ZJ. Cyclizidine-type alkaloids from *Streptomyces* sp. HNA39. J Nat Prod. 2018;81(2):394-9.

70. Asolkar RN, Kirkland TN, Jensen PR, Fenical W. Arenimycin, an antibiotic effective against rifampin- and methicillin-resistant *Staphylococcus aureus* from the marine actinomycete *Salinispora arenicola*. J Antibiot (Tokyo). 2010;63(1):37-9.

71. Jiang YJ, Zhang DS, Zhang HJ, Li JQ, Ding WJ, Xu CD, et al. Medermycin-type naphthoquinones from the marine-derived *Streptomyces* sp. XMA39. J Nat Prod. 2018;81(9):2120-4.

72. Zhang S, Xie Q, Sun C, Tian XP, Gui C, Qin X, et al. Cytotoxic kendomycins containing the carbacylic ansa scaffold from the marine-derived *Verrucosispora* sp. SCSIO 07399. J Nat Prod. 2019;82(12):3366-71.

73. Chen C, Ye Y, Wang R, Zhang Y, Wu C, Debnath SC, et al. *Streptomyces nigra sp. nov.* Is a Novel Actinobacterium Isolated From Mangrove Soil and Exerts a Potent Antitumor Activity in Vitro. Front Microbiol. 2018;9:1587.

74. Nguyen HT, Pokhrel AR, Nguyen CT, Pham VTT, Dhakal D, Lim HN, et al. *Streptomyces sp.* VN1, a producer of diverse metabolites including non-natural furan-type anticancer compound. Sci Rep. 2020;10(1):1756.

75. Cheng C, Othman EM, Stopper H, Edrada-Ebel R, Hentschel U, Abdelmohsen UR. Isolation of Petrocidin A, a New Cytotoxic Cyclic Dipeptide from the Marine Sponge-Derived Bacterium Streptomyces sp. SBT348. Mar Drugs. 2017;15(12).

76. Khan N, Yılmaz S, Aksoy S, Uzel A, Tosun Ç, Kirmizibayrak PB, et al. Polyethers isolated from the marine actinobacterium Streptomyces cacaoi inhibit autophagy and induce apoptosis in cancer cells. Chem Biol Interact. 2019;307:167-78.

77. Wang Q, Zhang Y, Wang M, Tan Y, Hu X, He H, et al. Neo-actinomycins A and B, natural actinomycins bearing the 5H-oxazolo[4,5-b]phenoxazine chromophore, from the marine-derived Streptomyces sp. IMB094. Sci Rep. 2017;7(1):3591.

78. Byun WS, Kim S, Shin YH, Kim WK, Oh DC, Lee SK. Antitumor Activity of Ohmyungsamycin A through the Regulation of the Skp2-p27 Axis and MCM4 in Human Colorectal Cancer Cells. J Nat Prod. 2020;83(1):118-26.

79. Um S, Choi TJ, Kim H, Kim BY, Kim SH, Lee SK, et al. Ohmyungsamycins A and B: cytotoxic and antimicrobial cyclic peptides produced by Streptomyces sp. from a volcanic island. J Org Chem. 2013;78(24):12321-9.

80. Lee J, Gamage CDB, Kim GJ, Hillman PF, Lee C, Lee EY, et al. Androsamide, a cyclic tetrapeptide from a marine *Nocardiopsis* sp., suppresses motility of colorectal cancer cells. Journal of Natural Products. 2020;83(10):3166-72.

81. Cui CB, Liu HB, Gu JY, Gu QQ, Cai B, Zhang DY, et al. Echinosporins as new cell cycle inhibitors and apoptosis inducers from marine-derived *Streptomyces albogriseolus*. Fitoterapia. 2007;78(3):238-40.

82. Hara M, Akasaka K, Akinaga S, Okabe M, Nakano H, Gomez R, et al. Identification of Ras farnesyltransferase inhibitors by microbial screening. Proc Natl Acad Sci U S A. 1993;90(6):2281-5.

83. Sattler I, Thiericke R, Zeeck A. The manumycin-group metabolites. Nat Prod Rep. 1998;15(3):221-40.

84. Di Paolo A, Danesi R, Nardini D, Bocci G, Innocenti F, Fogli S, et al. Manumycin inhibits ras signal transduction pathway and induces apoptosis in COLO320-DM human colon tumour cells. Br J Cancer. 2000;82(4):905-12.

85. Tuladhar A, Hondal RJ, Colon R, Hernandez EL, Rein KS. Effectors of thioredoxin reductase: Brevetoxins and manumycin-A. Comp Biochem Physiol C Toxicol Pharmacol. 2019;217:76-86.

86. Kim YH, Shin HC, Song DW, Lee SH, Furumai T, Park JW, et al. Arisostatins A induces apoptosis through the activation of caspase-3 and reactive oxygen species generation in AMC-HN-4 cells. Biochem Biophys Res Commun. 2003;309(2):449-56.

87. Trzoss L, Fukuda T, Costa-Lotufo LV, Jimenez P, La Clair JJ, Fenical W. Seriniquinone, a selective anticancer agent, induces cell death by autophagocytosis, targeting the cancer-protective protein dermcidin. Proc Natl Acad Sci U S A. 2014;111(41):14687-92.

88. Lu J, Ma Y, Liang J, Xing Y, Xi T, Lu Y. Aureolic acids from a marine-derived *Streptomyces* sp. WBF16. Microbiol Res. 2012;167(10):590-5.

89. Ratovitski EA. Tumor Protein (TP)-p53 Members as Regulators of Autophagy in Tumor Cells upon Marine Drug Exposure. Mar Drugs. 2016;14(8).

90. Chen L, Zhao W, Jiang H-L, Zhou J, Chen X-M, Lian Y-Y, et al. Rakicidins G - I, cyclic depsipeptides from marine *Micromonospora chalcea* FIM 02-523. Tetrahedron. 2018;74(30):4151-4.
